# Disordered development of gut microbiome interferes with the establishment of the gut ecosystem during early childhood with atopic dermatitis

**DOI:** 10.1080/19490976.2022.2068366

**Published:** 2022-04-29

**Authors:** Min-Jung Lee, Yoon Mee Park, Byunghyun Kim, in Hwan Tae, Nam-Eun Kim, Marina Pranata, Taewon Kim, Sungho Won, Nam Joo Kang, Yun Kyung Lee, Dong-Woo Lee, Myung Hee Nam, Soo-Jong Hong, Bong-Soo Kim

**Affiliations:** a Department of Life Science, Multidisciplinary Genome Institute, Hallym University, Chuncheon, Republic of Korea; b Asan Institute for Life Sciences, Asan Medical Center, Seoul, Republic of Korea; c Seoul Center, Korea Basic Science Institute, Seoul, Republic of Korea; d Department of Public Health Sciences, Graduate School of Public Health, Seoul National University, Seoul, Republic of Korea; e Department of Integrated Biomedical Science, Soonchunhyang Institute of Medi-Bioscience, Soonchunhyang University, Cheonan, Republic of Korea; f School of Food Science and Biotechnology, Kyungpook National University, Daegu, Republic of Korea; g Department of Integrative Biology, Kyungpook National University, Daegu, Republic of Korea; h Department of Biotechnology, Yonsei University, Seoul, Republic of Korea; i Department of Pediatrics, Childhood Asthma Atopy Center, Humidifier Disinfectant Health Center, University of Ulsan College of Medicine, Seoul, Republic of Korea; j The Korean Institute of Nutrition, Hallym University, Chuncheon, Republic of Korea

**Keywords:** Gut, microbiome, atopic dermatitis, development, homeostasis

## Abstract

The gut microbiome influences the development of allergic diseases during early childhood. However, there is a lack of comprehensive understanding of microbiome-host crosstalk. Here, we analyzed the influence of gut microbiome dynamics in early childhood on atopic dermatitis (AD) and the potential interactions between host and microbiome that control this homeostasis. We analyzed the gut microbiome in 346 fecal samples (6–36 months; 112 non-AD, 110 mild AD, and 124 moderate to severe AD) from the Longitudinal Cohort for Childhood Origin of Asthma and Allergic Disease birth cohort. The microbiome-host interactions were analyzed in animal and *in vitro* cell assays. Although the gut microbiome maturated with age in both AD and non-AD groups, its development was disordered in the AD group. Disordered colonization of short-chain fatty acids (SCFA) producers along with age led to abnormal SCFA production and increased IgE levels. A butyrate deficiency and downregulation of GPR109A and PPAR-γ genes were detected in AD-induced mice. Insufficient butyrate decreases the oxygen consumption rate of host cells, which can release oxygen to the gut and perturb the gut microbiome. The disordered gut microbiome development could aggravate balanced microbiome-host interactions, including immune responses during early childhood with AD.

## Introduction

The gut microbiome during early life influences immune system development that affects health in later life.^1,2^ Gut microbiome perturbations during early infancy increase the risk of developing allergic diseases in children.^3,4^ Several studies support the notion that the gut microbiome plays a critical role in manifesting allergic diseases.^5–8^ Gut microbiota, and the short-chain fatty acids (SCFA) they produce, can induce T_reg_ cells, which control mucosal Th_2_ inflammation.^9,10^ The Th_1_/Th_2_ balance is essential for immune regulation, and an imbalance Th_1_/Th_2_ can lead to chronic inflammation and allergic diseases.^11^

Atopic dermatitis (AD) is the most common chronic inflammatory skin disease, and its course is affected by the microbiome.^8,12–15^ The gut microbiome in infants with AD has low bacterial diversity, is deficient in *Bifidobacterium* and *Bacteroides*,^8,16,17^ and has perturbed functional genes related to host immune development.^13^ These perturbations result in imbalanced SCFA production, which dysregulates host-microbiome communication through immune and metabolic processes.^18^ Previous studies have compared the gut microbiome between infants with and without AD; however, these studies did not consider the changes in the microbiome with aging. Microbes colonize the gut since birth,^2,19^ and the microbiome dynamically changes until 36 months of age.^2^ The changes in the complex and mutual relationships between the gut microbiome and hosts with AD with aging remain unclear.

A recent study investigated the longitudinal changes in the gut microbiota in children with AD at early (5, 13, 21, and 31 weeks) and school ages (6–11 years) using 16S rRNA amplicon sequences.^20^ Although changes in microbiota associated with AD were observed, there was a lack of understanding of the microbiome functions as the study was solely based on 16S rRNA amplicon sequencing.^21^ Since the gut microbiome has highly balanced symbiotic interactions with the host,^22^ particularly in early life, a comprehensive analysis of the gut microbiome’s role in early childhood is a prerequisite to identify the mechanism underlying the host-microbiome symbiotic ecosystem in AD.

We investigated the relationship between gut microbiome dynamics in early childhood in children with AD and without AD and the potential interactions that control this relationship.

## Results

### Gut microbiota during early childhood exhibited two age-specific types (based on human study)

**Figure 1.**
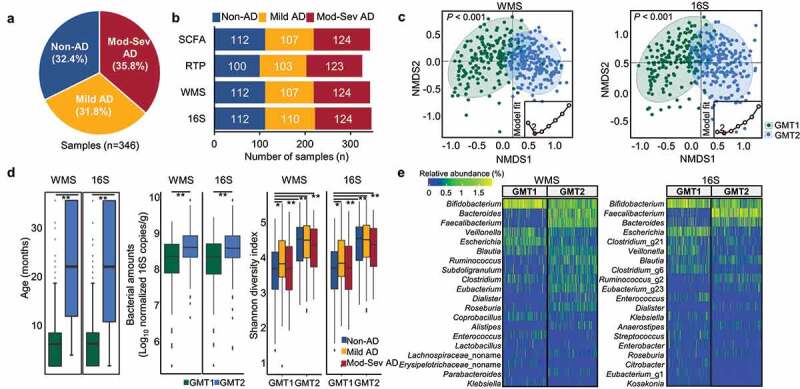
Summary of the analyzed data and comparison of the gut microbiota between clusters determined by Dirichlet multinomial mixture (DMM) modeling. (a) Overview of the studied samples. (b) Number of samples analyzed for each dataset. We analyzed 346 16S rRNA gene amplicon sequencing (16S), 343 whole metagenome sequencing (WMS), 326 quantitative real-time PCR (RTP), and 343 short-chain fatty acids (SCFA) profiles. (c) Microbiota clusters determined by DMM modeling in WMS and 16S dataset. The lowest Laplace approximation indicated that the number of clusters was two for gut microbiota data (inner graph). Two gut microbiota types (GMT1 and GMT2) were clearly distinguished in the NMDS plot (*P* < .001). (d) Comparison of age, bacterial amounts, and diversity between GMT1 and GMT2. (e) Heatmap of the relative abundance of the top 20 genera within two clusters. Mod-Sev AD: moderate to severe AD.*P < .05, **P < .01, ***P < .001.

We analyzed 346 fecal samples (6–36 months; non-AD = 112, mild AD = 110, and moderate to severe AD = 124) from the Longitudinal Cohort for Childhood Origin of Asthma and Allergic Disease birth cohort (Figure 1a and Figure S1). The sample size had a discriminatory power of 90%, with a significance of 0.05 (Table S1). Of these, 58.0% were boys, and 25.6% were born by cesarean delivery (Table S2). The scoring of atopic dermatitis (SCORAD) index, parental history of allergic diseases, serum eosinophils, total IgE, egg-, and milk-IgE were significantly different between non-AD and AD groups (*p* < .001). Solid food was introduced at a mean age of 5.6 months in the subjects of this study.

We analyzed 346 16S rRNA amplicon sequences (16S), 343 whole metagenome sequences (WMS), 326 quantitative real-time PCR (RTP) tests, and 343 SCFAs profiles in this study (Figure 1b and Table S3). Moreover, negative controls for the sampling tube, DNA extraction kit, and library preparation were sequenced and subsequently compared to the microbiota in samples (Figure S2). The microbiota detected in the negative controls differed from that in the samples, indicating that our sequence data was not influenced by potential contaminations.

Gut microbiota variation in all subjects (non-AD and AD groups) was analyzed using the Dirichlet multinomial mixture (DMM) model based on WMS and 16S datasets (Figure 1c). The microbiota was clustered within two gut microbiota types, GMT1 and GMT2, based on the lowest Laplace approximation (*p* < .001). Age was the significant factor distinguishing the two GMTs (Figure 1d; the median age of 10 months in GMT1 and 24 months in GMT2, *p* < .001), but AD diagnosis was not significantly different between GMTs (*p* > .05; Table S4). There were no significant differences in the odds ratio (OR) for covariates between GMT1 and GMT2 (*p* > .05) except age. The relative amounts and diversity of bacteria were higher in GMT2 than GMT1 (*p* < .01).

*Bifidobacterium, Veillonella*, and *Escherichia* were dominant genera in GMT1, whereas *Bacteroides, Bifidobacterium*, and *Faecalibacterium* were dominant in GMT2 (*q* < 0.001; Figure 1e, Table S5). These coincided with the DMM modeling results in each phenotypic group (Figure S3). Although WMS and 16S datasets showed similar results for DMM clustering, the relative abundances of each genus were different. Therefore, we compared the overall concordance of relative abundances between WMS and 16S at phylum and genus levels (Figure S4). Pearson correlation R values of WMS and 16S were over 0.52 in all subjects (*p* < .001) at the phylum level and decreased (R ≤ 0.31; *p* < .001) at the genus level. Moreover, detected genera in negative controls for WMS were clearly different from those in samples, and detected numbers were lower (2 genera) than in 16S (19 genera) (Figure S2). Therefore, we primarily used the WMS dataset for further analyses.

### Age-dependent assembly of gut microbiota was perturbed in childhood with AD (based on human study)

**Figure 2.**
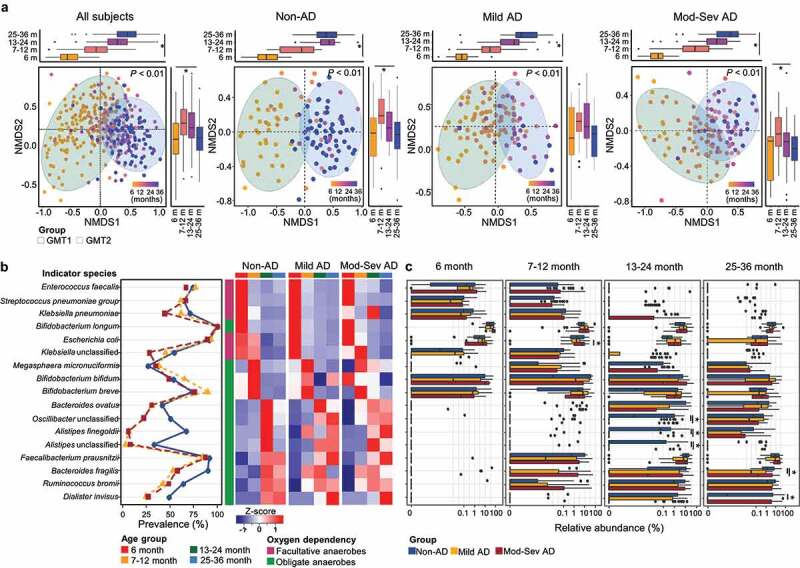
Age-dependent shifts of the gut microbiota in each group. (a) Variation in the gut microbiota according to age in all subjects, non-AD, mild AD, and moderate to severe AD groups were analyzed using NMDS plots based on the species-level Bray–Curtis dissimilarity. The boxplot shows the variation of Bray–Curtis dissimilarity among age groups for each axis. (b) Shifts of indicator species for each age throughout early childhood. Heatmap shows the comparison of the indicator’s relative abundance according to age. Color codes for the age groups and the characteristics of oxygen dependency are listed below the figure. (c) Comparison of the relative abundance of each indicator among groups according to age. Mod-Sev AD: moderate to severe AD. *q < 0.05.

Although the early childhood gut microbiota was clustered within two types, previous studies reported that the gut microbiota dynamically changes with age during early life.^2,23^ Furthermore, solid food intake starting at a mean age of 5.6 months in subjects of this study could influence their gut microbiome (after 6 months). Therefore, we analyzed the gut microbiota using the Bray–Curtis dissimilarity according to age range (6, 7–12, 13–24, 25–36 months) in non-metric multidimensional scaling (NMDS) plots (Figure 2a). The microbiota varied with age within GMT1 and GMT2 for all subjects and each phenotype (*p* < .01).

We analyzed the indicator species for each age to study the shifts in the gut microbiota with age in each phenotypic group (Figure 2b, Table S6). Indicator species gradually shifted with age in the non-AD group, while they showed abrupt changes in AD groups. In the non-AD group, facultative anaerobes were indicators until 12 months, and strict anaerobes were indicators after 12 months. However, their relative abundances in the AD groups were different from those in the non-AD group with age. The relative abundance of facultative anaerobes in AD microbiomes was higher than in non-AD microbiomes during early life. In addition, they differed between mild and moderate to severe AD groups (*p* < .05; Figure S5a).

At 6 months, *Bifidobacterium longum* was a predominant indicator in all groups, and there were no significantly different species according to AD severity (*q* > 0.05; Figure 2c). *B. bifidum* and *B. breve* were dominant indicators at 7–12 months, and the relative abundance of *Escherichia coli* was higher in the moderate to severe AD than mild AD group at this age (*q* < 0.05). The higher proportions of *Alistipes finegoldii*, unclassified (UC)_*Oscillibacter*, and UC_*Alistipes* were detected in the non-AD than in AD groups at 13–24 months (*q* < 0.05). *Faecalibacterium prausnitzii, Bacteroides fragilis, Ruminococcus bromii*, and *Dialister invisus* were indicators at 13–36 months, and the relative abundances of *B. fragilis* and *D. invisus* were higher in the non-AD than in AD groups at 25–36 months (*q* < 0.05). Indicators for each age were evaluated using the multivariate association with linear models (MaAsLin2) after adjusting for covariates (Table S7). Indicators were significantly associated with age after adjustments except for *B. bifidum* and *A. finegoldii*.

We analyzed the significantly associated covariates with microbiota at each age using the EnvFit model (Table S8). Breastfeeding and AD severity were significantly associated with gut microbiota at 6–24 months. In particular, exclusive breastfeeding (EBF) was the most significant and common factor (r^2^ = 0.306, *p* < .001 in WMS) for the gut microbiota differences in both non-AD and AD groups at 6 months. The relative abundance of *B. longum* was higher in EBF infants than in non-EBF infants, whereas *B. breve* and UC_*Coprobacillus* were higher in non-EBF infants (*p* < .05; Figure S5b).

### Estimated microbiota age and network analyses revealed disordered gut microbiome development in AD groups (based on human study)

**Figure 3.**
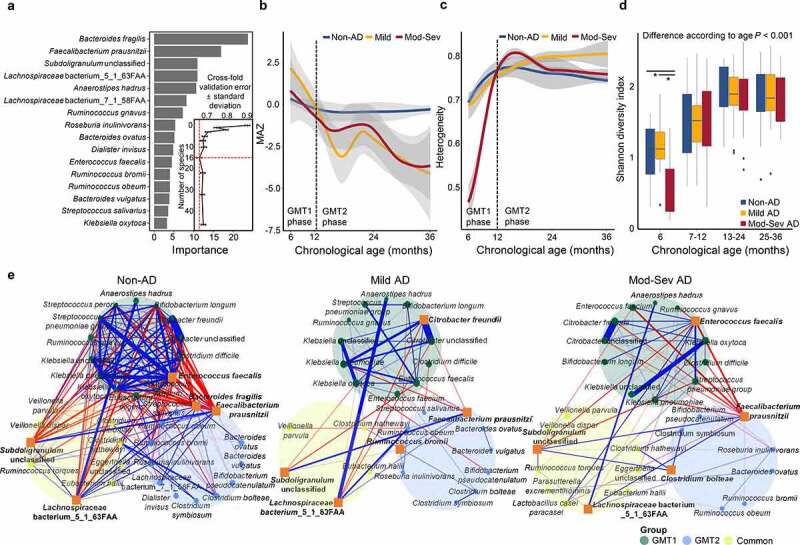
Disordered gut microbiota development in AD groups. (a) Species importance in the prediction model of EMA in 112 non-AD samples. The 16 species with the most discriminating power were selected by the lowest cross-validation error (inner graph). (b) Comparison of EMA through early childhood among groups by MAZ score. (c) Heterogeneity of gut microbiota within each group throughout early life. The gray area represents the 95% confidence intervals (CIs). The black dashed line indicates the time point at which the change in the MAZ score of AD groups occurs. (d) Difference of Shannon diversity index among groups in each age. (e) Network analysis of the top 10 species in each microbiota type for each phenotype determined by random forest modeling. The common group included commonly detected species in GMT1 and GMT2. Positive correlations are marked by blue edges and negative correlations by red edges. Edge thickness denotes FastSpar correlation, ranging from value −0.4 to 0.4. Node sizes were scaled on the eigenvector centrality measure. Only significant correlations with *P* < .05 are shown. The top 5 network hubs determined by the PageRank algorithm are marked in bold with an Orange square symbol. Mod-Sev AD: moderate to severe AD. *P < .05.

We compared the species profiles by age to analyze the gut microbiome development during early life according to AD severity via random forest modeling. *Bacteroides fragilis* and *F. prausnitzii* were the most influential species in the prediction model (Figure 3a). Gut microbiota in the AD group was highly mature compared to that in the non-AD group at 6 months, whereas delayed gut microbiota maturation was observed after 12 months in the microbial-by-age z-score (MAZ) analysis (Figure 3b). *Bacteroides fragilis,* UC*_Subdoligranulum, Anaerostipes hadrus*, and *Roseburia inulinivorans* were significantly correlated to low MAZ in the AD group after 12 months (*p* < .05; Figure S6a).

The microbiota heterogeneity was the lowest in the moderate to severe AD group at 6 months (Figure 3c) but increased to the level of other groups after 12 months. Heterogeneity of microbiota in the mild AD group was higher than in other groups after 24 months. The Shannon diversity increased with age in all groups (*p* < .001), and the diversity was significantly lower in the moderate to severe AD than in non-AD at 6 months (*p* < .05; Figure 3d). Differences in diversity between groups according to AD severity were observed at 6 months and 25–36 months in the 16S dataset, and 7–12 months and 25–36 months in the functional gene dataset (*p* < .05; Figure S6b).

Different microbiota development in AD groups could be partly driven by interactions between species in the gut microbiota. Thus, we analyzed the correlations between species in GMT1 and GMT2 of each phenotype group because gut microbiota was clearly distinguished into two age-specific types throughout early childhood (Figure 1). Commonly detected bacteria in both GMTs could be intermediate members during gut microbiome development.

More complex interspecies correlations in the non-AD group than in the AD groups indicated that dynamic drift and diversification occurred through interactions during gut microbiome development in the non-AD group (Figure 3e). Positive correlations were more abundant in GMT1 than in GMT2 and the common groups. *Enterococcus faecalis* was a hub with positive correlations with bacteria in GMT1. UC_*Subdoligranulum* and *Lachnospiraceae* bacterium_5_1_63FAA were keystones during gut microbiome development. *Faecalibacterium prausnitzii* and *B. fragilis* were hubs in the gut microbiota of GMT2 with negative correlations with GMT1 members and positive correlations with bacteria in GMT2.

*Citrobacter freundii* and *E. faecalis* were keystones in GMT1 of mild and moderate to severe AD, respectively. Although UC_*Subdoligranulum* and *Lachnospiraceae* were also keystones during microbiome development in AD groups, there were fewer correlated bacteria than in the non-AD group, and their correlations differed. *Faecalibacterium prausnitzii* was also a hub in GMT2 of AD groups. Therefore, the same bacteria could be critical to gut microbiome development in all groups. However, their influences on the microbiota were different in AD groups according to severity. This could be related to the disordered gut microbiome development with lower relative abundances of *B. fragilis* and UC_*Subdoligranulum* in AD groups (Figure S6a). Network analysis for all subject and combined AD groups revealed similar results as that of the distinguished phenotypes (Figure S7).

### Disordered microbiota development in AD groups altered SCFA profiles with age (based on human study)

**Figure 4.**
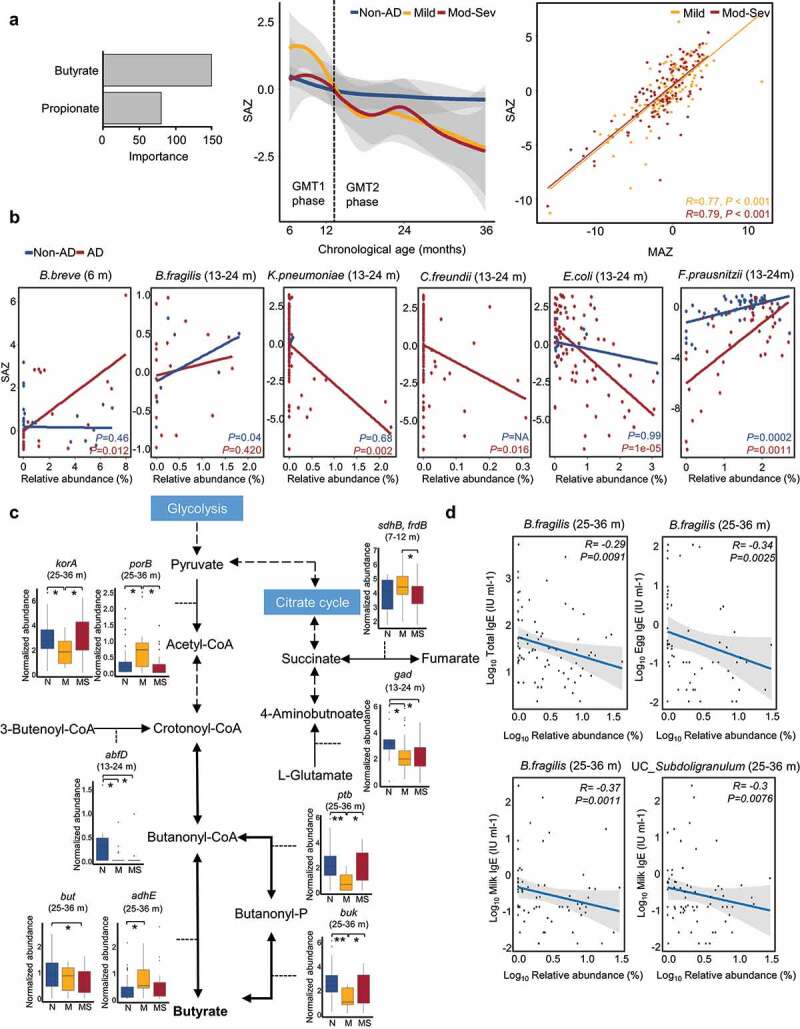
Dysbiosis of short-chain fatty acids (SCFAs) profiles in AD groups throughout early childhood. (a) Dysbiosis of SCFAs profiles in AD groups according to age. The alteration of SCFAs in AD groups was analyzed using the prediction model of SCFAs changes according to age in 112 non-AD samples. The importance of butyrate and propionate for the prediction model was determined using subjects without AD. The SCFAs-by-age z-score (SAZ) was calculated to identify dysbiosis of SCFA profiles in AD groups along with age. The gray area represents the 95% confidence intervals (CIs). The correlation between SAZ and MAZ was determined by linear regression models and the Pearson correlation test. (b) Species that are significantly associated with the dysbiosis of SCFAs (SAZ score) in AD groups at each age. (c) Comparison of gene families involved in butyrate metabolism among groups. The boxplot shows the significantly different gene families between the non-AD and AD groups. (d) Correlation between the relative abundances of significantly associated species with abnormal MAZ or SAZ in AD groups and IgE levels. *Bacteroides fragilis* and unclassified (UC)_*Subdoligranulum* were detected as associated species with *P*-value < 0.01. Mod-Sev AD: moderate to severe AD. N: non-AD, M: mild AD, and MS: moderate to severe AD. *q < 0.05, **q < 0.01.

Differences in AD gut microbiome development can cause alterations in SCFA profiles. Therefore, we compared SCFA profiles among groups by training a machine-learning algorithm. SCFA-by-age z-score (SAZ) was calculated to analyze the dysbiosis of SCFA profiles in AD groups along with age (Figure 4a). Butyrate contributed more significantly to the prediction model than propionate. The SCFA profiles in AD groups were highly matured compared to those in non-AD before 12 months. However, they delayed maturation after 12 months, consistent with gut microbiota development (correlation R value between SAZ and MAZ ≥ 0.77 with *p* < .001). These results indicated that disordered gut microbiota development was associated with dysregulated SCFA production.

The relative abundances of *B. breve* in AD groups were significantly associated with high SAZ at 6 months (*p* = .012; Figure 4b). Conversely, the relative abundances of facultative anaerobes (*K. pneumoniae, C. freundii*, and *E. coli), B. fragilis*, and *F. prausnitzii* were related to low SAZ in AD groups after 12 months (*p* < .05).

Gene families involved in butyrate metabolism were compared according to AD severity (Table S9 and Figure S8), and the significantly different gene families between non-AD and AD groups (*q* < 0.05 in Dunn’s test and *q* < 0.25 in MaAsLin2 after adjustments) were analyzed (Figure 4c). L-glutamate usage for butyrate production through glutamate decarboxylase (*gad*) was lower in AD groups than in non-AD group after 12 months. The conversion between fumarate and succinate by succinate dehydrogenase/fumarate reductase (*sdhB*/*frdB*) was lower in moderate to severe AD at 7–12 months. Different genes were used to convert pyruvate to acetyl-CoA in mild AD (*porB*) compared to other groups (*korA*) at 25–36 months. Crotonoyl-CoA production from 3-butenoyl-CoA by 4-hydroxybutanoyl-CoA dehydrase (*abfD*) was reduced in AD groups at 13–24 months. Butyryl-CoA: acetate-CoA transferase (*but*) levels at 25–36 months were lower in AD groups than non-AD group, and acetaldehyde dehydrogenase (*adhE*) levels were higher in the mild AD group than in other groups. Phosphate butyryltransferase (*ptb*) and butyrate kinase (*buk*) levels were the lowest in the mild AD group at 25–36 months. Ten species (*A. findgoldii, A. hadrus, B. fragilis, B. ovatus, Coprococcus eutactus, Eggerthella lenta, Lachnospiraceae* bacterium, *Megamonas hypermegale, Odoribacter splanchnicus*, and *Prevotella buccae*) were significantly different between non- and AD groups, as corresponding bacteria (*q* < 0.05; Figure S9). These differences could cause deficient butyrate production and low SAZ in AD groups. Differences according to severity were also found. Gene families involved in propionate metabolism were also different among groups according to severity (Table S10 and Figure S10).

### Potential influence of disordered microbiome development on host immune responses in AD groups was revealed by functional features (based on human study)

The associated species with abnormal MAZ and SAZ in AD groups were correlated with clinical features (Figure 4d). The relative abundances of *B. fragilis* and UC_*Subdoligranulum* were negatively correlated with total IgE, egg-, and milk-IgE at 25–36 months (*p* < .01). In particular, *B. fragilis* was related to both abnormal MAZ and SAZ in AD groups, suggesting that the disordered microbiome development and SCFA production by limited colonization of *B. fragilis* was related to IgE levels and sensitization to egg or milk in children with AD.

**Figure 5.**
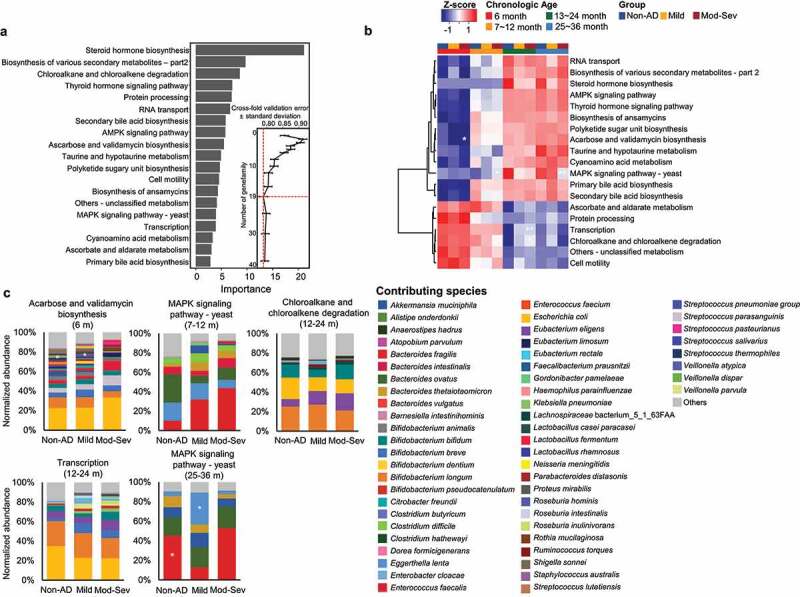
Normalized abundances of KEGG Orthology (KO) among groups according to age. (a) The top 19 KOs with the most discriminative power were selected. Functional gene features in the gut microbiome of the non-AD group were used to determine significantly changed KOs according to age by MaAsLin2 after adjustment with other covariates (*q* < 0.05). Among the age-dependent KOs, 19 KOs were selected by the lowest cross-fold validation error. (b) Selected KOs were compared among groups at each age in the heatmap. The colors of the heatmap correspond with the z-score for normalized abundances of functional genes. (c) Comparison of contributing species to significantly different KOs at each age. Statistical significance was calculated using the Kruskal–Wallis test and Dunn’s test. *P*-value was adjusted using the Benjamini–Hochberg method to calculate the *q* value. The gradually changing KOs according to AD severity are indicated by significance (*P* < .05 in the Kruskal test and *q* < 0.05 in Dunn’s test). Mod-Sev: moderate to severe AD. *q < 0.05, **q < 0.01.

The gut microbiome may play different roles by disordered microbiota development in AD groups. We analyzed the alteration of functional features in the gut microbiome along with age based on KEGG Orthology (KO). The differential KOs by age in the non-AD group were selected by MaAsLin2 after adjustment for covariates. A total of 327 KOs significantly altered with age (*q* < 0.05). We selected the top 19 most discriminatory features by random forest model with the lowest cross-fold validation error (Figure 5 and Table S11). Differences in functional features were distinguished by two clusters consistent with DMM clustering. Relative abundances of 6 KOs were higher in the GMT1 dominant phase (6–12 months), and those of 13 KOs were higher in the GMT2 dominant phase (13–36 months).

Four KOs gradually changed according to AD severity at each age (*p* < .05 in Kruskal–Wallis test and *q* < 0.05 in Dunn’s test). The relative abundance of acarbose and validamycin biosynthesis was higher in non-AD than in AD groups at 6 months. The MAPK signaling pathway showed the highest levels in moderate to severe AD at 7–12 months. Chloroalkane and chloroalkene degradation and transcription were higher in moderate to severe AD at 13–24 months. MAPK signaling pathway showed higher levels in non-AD than in AD groups at 25–36 months. Species contributed to these KOs differed among the AD severity groups.

### Interactions between gut microbiome and host cell were perturbed in the AD group (based on animal and *in* *vitro* cell studies)

**Figure 6.**
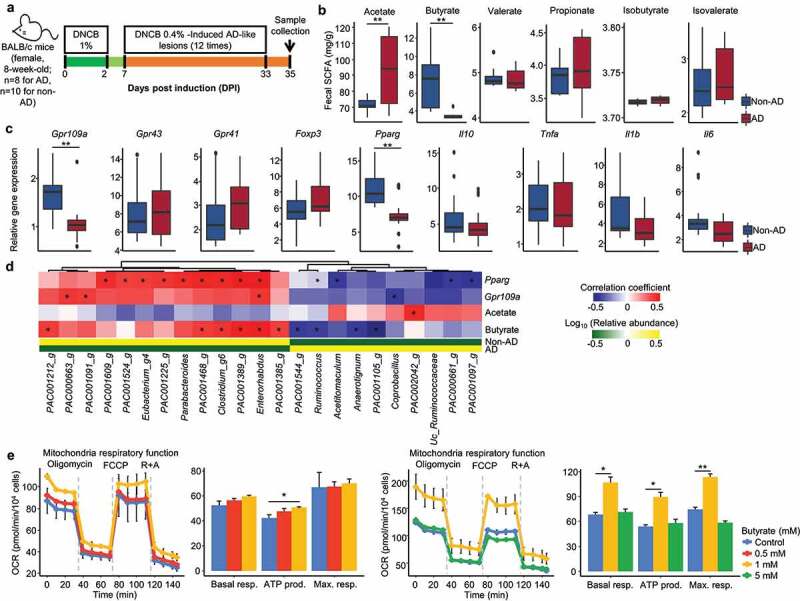
The interactions between gut microbiome and host cell in animal and *in vitro* cell assays. (a) Design for the mice experiment. Female BALB/c mice were used, 1% DNCB was applied to the shaved dorsal skin three times for one week. 0.4% DNCB was applied three times a week for four weeks. Mice were sacrificed at day 35, and the middle part of the colon and fecal samples were collected. (b) The concentration of SCFAs was compared between non-AD and AD groups. SCFAs were measured by GC-MS from fecal samples. (c) The expression of target genes in the middle part of the colon was compared between non-AD and AD groups. The expression levels of SCFA receptor (*Gpr109a, Gpr43*, and *Gpr41*), transcription factors (*Foxp3* and *Pparg*), anti-inflammatory cytokine (*Il10*), and pro-inflammatory cytokines (*Tnfa, Il1b*, and *Il6*) were analyzed. (d) Significantly correlated genera in fecal samples of mice with *Pparg, Gpr109a*, acetate, and butyrate. (e) Effects of butyrate on oxygen consumption rate (OCR) of Caco-2 cells. The OCR of each treated cell was investigated using a Seahorse Bioscience XF24 extracellular flux analyzer. Basal respiration (Basal resp.), ATP-linked respiration (ATP prod.), and maximal respiration (Max. resp.) were compared among treated butyrate concentrations. Results are presented as the mean ± SD. *P < .05, **P < .01.

The interactions between the perturbed gut microbiome and host cell were analyzed using animal and *in vitro* cell assays. The gut microbiota, SCFA production, and host gene expression were compared between non-AD and AD-induced mice (Figure 6a). The concentration of butyrate was lower in fecal samples of the AD group than the non-AD group, whereas acetate was higher in the AD group (*p* < .01; Figure 6b). The expression level of genes for G-protein coupled receptor 109A (*Gpr109a*) and peroxisome proliferator-activated receptor-γ (*Pparg*) were downregulated in the colon of the AD group compared to the non-AD group (*p* < .01; Figure 6c). Positively correlated gut microbes with *Pparg, Gpr109a*, and butyrate were more abundant in the non-AD group than in the AD group (*p* < .05; Figure 6d).

Our results clearly show that butyrate production by the gut microbiome in the AD group was disturbed in both human and animal studies; hence, the influence of butyrate on colon epithelial cells was analyzed by the oxygen consumption rate (OCR) in Caco-2 cells (Figure 6e). Butyrate enhanced OCR and mitochondrial respiration processes. However, this effect was only detected at 1 mM concentration of butyrate, which showed that an optimal concentration of butyrate is required to maintain homeostasis of host cell metabolism.

## Discussion

We analyzed the gut microbiome of children with AD during early childhood (6–36 months) according to severity. The ecological drift of the gut microbiome during early life, from facultative anaerobes to strict anaerobes, was irregular in AD groups. The disordered microbiome development in AD was contributed to butyrate deficiency, different microbiome’s functional genes, and increased IgE levels. Perturbed gut microbiota and deficient butyrate interacted with gene expressions and OCR in host cells. The perturbed microbiome-host crosstalk could contribute to AD during early childhood.

The gut microbiota in early childhood was clustered into two age-specific types, GMT1 and GMT2, regardless of AD phenotype. Age-dependent clustering is consistent with previous findings,^2^ resulting from synergistic influences, such as immune system development and the transition to solid food during early life.^19,24^

The age-dependent gut microbiome development during early childhood is critical for understanding their influences on AD, since microbial diversity and specific taxa could be linked to AD. Some studies have reported that the gut microbiota of AD patients is less diverse than non-AD patients.^16,25^ In contrast, other studies have found no significant differences in microbiota diversity between the groups.^13,26^ *Bifidobacteria* are suggested to be beneficial,^27,28^ but are overrepresented in children with allergies at a later age.^7^ *Faecalibacterium, Lachnospira, Veillonella*, and *Rothia* in the microbiota of children aged 3 months are risk factors for developing atopic wheeze, but not at 12 months.^29^ We found that the diversity of gut microbiota in moderate to severe AD decreased compared to the non-AD group at only 6 months through WMS. *Bifidobacterium* was not associated with AD, as previously reported.^13,20,30,31^ Therefore, the disturbed gut ecosystem could be more critical in AD progression during early childhood than single taxa.

Although the gut microbiota developed with age in both non-AD and AD groups, the development in the AD groups was disordered, as determined by the MAZ analysis. We found that the microbiota of children with AD over-matured before 12 months compared to those of non-AD children and had delayed development at 13–36 months. Shifts of indicator species at each age and interspecies interactions indicated the disordered gut microbiome development in AD groups. The order and timing of microbe colonization affect interspecies interactions during gut microbiome development.^32^

Early colonizers can affect the subsequent colonization of gut microbial populations. Interspecies interactions in AD microbiomes were reduced compared to the non-AD microbiome, particularly in GMT1, and these interactions varied according to AD severity. Also, the reduced interactions in GMT1 persisted in GMT2. This provides evidence for the influence of priority effects on the gut microbiome during early development. The low heterogeneity and diversity of moderate to severe AD gut microbiota at 6 months partially supports this assertion. Reduced interspecies interactions at early phases caused reduced interactions at intermediate and later phase (GMT2). This can cause disordered microbiome development and abruptly shift indicator species along ages in the AD groups.

The SCFA dysbiosis with age in AD groups (SAZ analysis) was consistent with MAZ results. Lower SCFA levels and butyrate producers in early life are associated with atopic disease development.^14,33,34^ The maturation of the gut microbiome and its metabolome with age are a synchronized process that regulates gut maturation and immune development.^35^ Therefore, SCFA dysbiosis resulting from disordered gut microbiome development during early childhood can be related to AD.

The relative abundance of facultative anaerobes throughout early childhood was higher in AD groups than in non-AD group in the present study. Early colonizing facultative anaerobes gradually shift to strict anaerobes by utilizing oxygen to create an anaerobic gut environment.^19,36^ The higher abundance of facultative anaerobes in AD groups than in non-AD group could indicate that the redox balance of the gut environment throughout early childhood is disturbed for those with AD. Low SAZ score in AD groups was related to the relative abundances of facultative anaerobes and strict anaerobes including *B. fragilis*.

Lower *B. fragilis* proportions were also related to low MAZ scores, reduced interspecies interactions, and increased IgE levels in AD groups compared to non-AD. In particular, IgE and specific IgE to food allergen play a critical role in the pathogenesis of AD and food allergy.^37–39^ A reduced abundance of *B. fragilis* in the AD group, which prevents inflammation through its polysaccharide A by restoring Th_1_/Th_2_ balance,^40^ was also found in previous studies.^16,27^ These results suggest that the redox-dysregulated gut environment of children with AD during early development could cause gut microbiome perturbation and SCFA dysbiosis in later life by limiting the colonization of *B. fragilis*. This could cause increasing IgE and egg/milk-IgE levels in AD.

The potential influence of disordered gut microbiome development on host immune responses in AD groups was found in functional features analysis. Functional genes in the microbiome related to acarbose and validamycin biosynthesis and MAPK signaling pathway were gradually changed according to AD severity. The anti-inflammatory effect of acarbose has been previously reported in the mouse model of inflammation.^41^ MAPK signaling pathway was higher in AD groups at the earlier phase, whereas this pathway was higher in non-AD group at the later phase. This pathway is important for cell growth, differentiation, and inflammation,^42,43^ and therefore may play different roles in the gut through different contributing species at early and later phases of early childhood.

**Figure 7.**
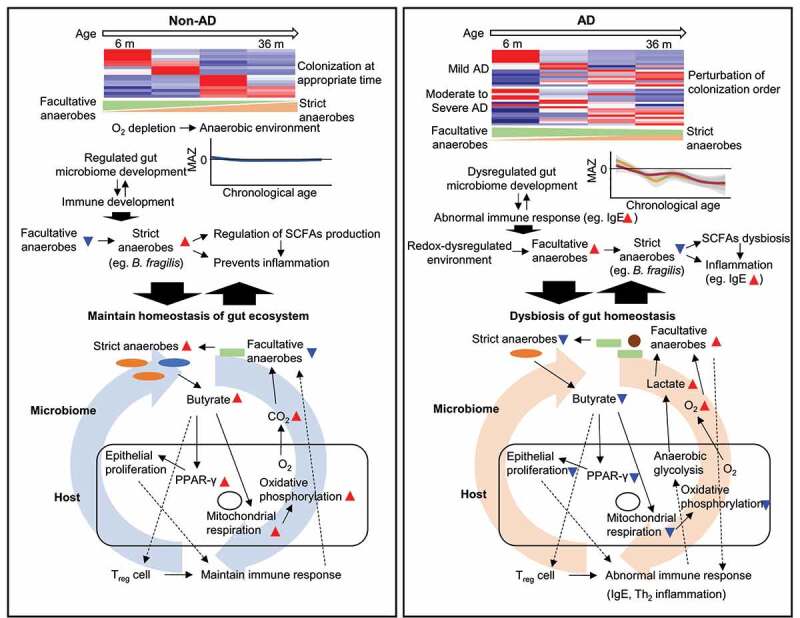
Hypothetical model for microbiome-host interactions in the gut ecosystem of AD during early life. In the non-AD group, the gut microbiome develops during early childhood by colonizing microbes at the appropriate time. Early colonizing facultative anaerobes gradually shift to strict anaerobes by utilizing oxygen to create an anaerobic gut environment. Adequate maturation of the gut microbiome regulates SCFAs production and immune development throughout early childhood. Butyrate produced by strict anaerobes activates PPAR-γ, mitochondrial respiration, and increases oxygen consumption through oxidative phosphorylation. Thus, it maintains an anaerobic environment by reducing oxygen emanation from the mucosa. Moreover, the butyrate induces T_reg_ cells, which control Th_2_ inflammation. In the AD group, this cyclic interaction is imbalanced, exacerbating the homeostasis of the gut ecosystem. An abnormal oxygen environment in the gut maintains facultative anaerobes during childhood; it limits *B. fragilis* colonization throughout the lifespan. Disordered gut microbiome development is related to decreased butyrate production and abnormal immune responses. PPAR-γ and T_reg_ cells cannot be activated with a decrease in butyrate. The epithelial proliferation and mitochondrial respiration decrease, and the emanating oxygen and lactate from mucosa increase by anaerobic glycolysis of the host cell. Facultative anaerobes use lactate produced by the host cell. Abnormal T_reg_ and epithelial cell proliferation and the increased numbers of facultative anaerobes induce abnormal immune responses.

The interaction between the perturbed gut microbiome and host cell in the AD group was evaluated in animal studies and *in vitro* cell assays. Deficient butyrate was caused by perturbed gut microbiota in the AD-induced mice, resulting in the downregulated expression of *Gpr109a* and *Pparg* in the colon. GPR109A is a receptor for butyrate,^44^ and butyrate activates PPAR-γ signaling, which induces epithelial cell proliferation, oxidative phosphorylation, and immune development.^45–47^ In addition, butyrate enhanced the OCR of the host cell, which corresponds to an increase in mitochondrial oxidative phosphorylation and a decrease in anaerobic glycolysis.^48,49^ When the host’s metabolism shifts toward anaerobic glycolysis, it decreases oxygen consumption and increases glucose consumption and lactate release,^50,51^ driving the proliferation of facultative anaerobes.^52^ These influence the differentiation of T_reg_ cells and immune responses.^10,53^ This circular crosstalk can accelerate gut ecosystem dysbiosis. Therefore, the disordered gut microbiome development and SCFA dysbiosis in children with AD affect gut homeostasis, including the immune system. Our results advance the understanding of the gut microbiome-host interactions in AD during early childhood (Figure 7).

Since it is challenging to evaluate human gut microbiome development using animal models,^54,55^ we could not apply microbiome development states in mice. This can be improved with advancements in omics technologies, *in vitro*, and humanized *in vivo* models in the future. Although differences of microbiome’s functional gene among phenotypic groups were found by whole metagenome in this study, further studies will be necessary to clarify these findings by other techniques including metatranscriptomic and metaproteomic analyses. Furthermore, we could not identify the causative covariates of gut microbiome development in this study. This could be due to the limited covariates obtained or complex interactions in the gut ecosystem. Nevertheless, our study is significant because it advances the hypothesis regarding the influence of the gut microbiome on the pathogenesis of AD through host-microbiome interactions during early life. This can help develop novel prediction and treatment strategies for AD in early life.

In conclusion, regulated gut microbiome development is essential to maintain the gut ecosystem during early childhood. Disordered microbiome development in AD is characterized by persisting facultative anaerobes and limited *B. fragilis* colonization with age, which reduces SCFA production and induces abnormal immune responses by increasing IgE. This relationship is dynamic and harmonious crosstalk of a symbiotic human-microbiome system. Thus, the early stages of microbiome development may be an appropriate target for modulating the microbiota and establishing a healthy microbiome. Understanding these symbiotic relationships is critical for developing preventive and therapeutic strategies to treat AD in early childhood.

## Material and methods

### Study subjects and sample collection

The statistical power of the sample number per group was estimated using the micropower R-package based on permutational multivariate analysis of variance (PERMANOVA).^56^ The PERMANOVA powers were calculated based on the weighted UniFrac distance of infant gut microbiota data in our previous study.^13^ The effect sizes (ω^2^) were calculated using the simulated matrixes of 80% and 90% powers for varying sample numbers per group (Table S1). One hundred bootstrap iterations were performed using a significance level of 0.05 to estimate the power and effect size. The effect size was smaller than 0.0001 in ≥ 100 samples per group for a discriminatory power of 90%. Therefore, we aimed to collect more than 100 samples per group in this study.

Subjects in this study were recruited from the Longitudinal Cohort for Childhood Origin of Asthma and Allergic Disease (COCOA) birth cohort (over 3,000 infants enrolled) and Childhood Asthma Atopy Asan Medical Center.^57^ The collection of data and biological samples in the COCOA study is conducted every year for all enrolled children from 6 months to 20 years old, regardless of allergic disease development. Pediatric allergists diagnosed AD according to Hanifin and Rajka’s criteria.^58^ AD severity was simultaneously assessed at fecal collection using the SCORAD index (mild < 25 and moderate to severe ≥ 25).^59^ Total IgE and egg- and milk-specific serum IgE (IU/mL) levels were measured using fluorescent enzyme immunoassay (AutoCAP system, Phadia AB, Uppsala, Sweden) after 12 months of age for all subjects. Subjects without any visible sign of skin eczema indicative of AD from 6 to 36 months and without food sensitization (< 0.35 of specific IgE to egg and milk) were recruited into the non-AD group. Subjects who received antibiotics during 6 months preceding collection and had health complications, including gestational age < 37 weeks, smoking exposure during pregnancy, any major congenital anomalies, and birth asphyxia requiring oxygen supplementation, were excluded from the study. Collected clinical data and biological samples in this study were summarized in the sampling strategy (Figure S1).

A total of 346 samples (112 non-AD, 110 mild AD, and 124 moderate to severe AD) from 6 to 36 months old were collected from the COCOA cohort. Fecal samples were collected from five university hospitals in Seoul, and each center transported samples in iceboxes to the laboratory within 4 hours after sample collection. Samples were immediately stored at −80°C before being processed for DNA extraction.

This study was approved by the Institutional Review Boards (IRBs) of Asan Medical Center (IRB no. 2008–0616 and 2015–1031), Samsung Medical Center (IRB no. 2009–02-021), Severance Hospital (IRB no. 4–2008-0588), CHA Medical Center (IRB no. 2010–010), and Seoul National University Hospital (IRB no. H-1401-086-550). Written informed consent was obtained from the parents of each infant.

### 16S rRNA gene amplicon sequencing

Metagenomic DNA was extracted from fecal samples using the RNeasy PowerMicrobiome Kit (Cat #26000-50, Qiagen, Valencia, CA, USA) following the manufacturer’s instructions. The extracted DNA was purified using the DNeasy PowerClean Pro Cleanup Kit (Cat #12997-50, Qiagen) and quantified using a BioPhotometer D30 with a μCuvette G1.0 (Eppendorf, Hamburg, Germany). The V1–V3 region of the 16S rRNA gene was amplified using a C1000 thermal cycler (Bio-Rad, Hercules, CA, USA) per the MiSeq system protocol for preparing a 16S metagenomics sequencing library (Illumina, Inc., San Diego, CA, USA), as described previously.^60,61^ Equimolar concentrations of each sample were pooled and sequenced using the Illumina MiSeq system (300-bp paired ends) according to the manufacturer’s instructions. Previous studies reported the influence of potential contamination of reagents on sequence-based microbiome analyses.^62,63^ Therefore, we sequenced and analyzed negative controls for quality control of sequencing. Negative controls were included at every step to check contamination, and three negative controls were sequenced with samples. Sequenced negative controls included unused stool box (sampling blank), DNA-free water added to the RNeasy PowerMicrobiome Kit (negative extraction controls), and the library preparation instead of DNA (library negative controls).

Amplicon sequences were analyzed using the QIIME2 pipeline.^64^ Raw sequences were quality filtered, and denoised using DADA2, and the taxonomic position of representative sequences were assigned with the EzTaxon-e database.^65^ Diversity indices were calculated after rarefied without replacement. A total of 28,804,166 reads (median 68,045 reads per sample) in human fecal samples were obtained from sequence analyses.

### Whole metagenome shotgun sequencing

Extracted DNA from 346 samples was fragmented using a NEBNext dsDNA Fragmentase (Cat #0348 L, New England Biolabs, Ipswich, MA, USA). Then, metagenomic libraries were prepared using the ACCEL-NGS 2S PLUS DNA Library Kits (Cat #21096, Swift Biosciences, Ann Arbor, MI, USA) according to the manufacturer’s instructions.

The size of the libraries was confirmed using a Bioanalyzer 2100 (Agilent Technologies, Santa Clara, CA, USA). The concentration of the library was measured using a PicoGreen dsDNA Assay kit (Cat #P11496, Invitrogen, Carlsbad, CA, USA). Equimolar concentrations of each library were calculated by qPCR using a TaKaRa PCR Thermal Cycler Dice Real Time System III (TaKaRa Bio, Inc., Shiga, Japan) with the GenNext NGS Library Quantification Kit (Cat #NLQ-101, Toyobo, Osaka, Japan).

Libraries were pooled and sequenced using the Illumina HiSeq 2500 system (250-bp paired ends). Three samples were excluded due to failed sequencing library preparation. Thus, a total of 343 samples were analyzed. For quality control of shotgun sequencing, negative controls were processed following the same procedures to check contamination, and two kinds of negative control were sequenced with samples. The two negative controls were DNA-free water added to the RNeasy PowerMicrobiome Kit (negative extraction controls) and the library preparation instead of DNA (library negative controls).

WMS were analyzed as described previously.^13,66^ Briefly, adapter removal and quality filtering were performed using Trimmomatic with default options (leading and trailing: 3, sliding window size: 4, quality: 15, min length: 150).^67^ Paired-end sequences were merged using PEAR v.0.9.11.^68^ Contaminated human genes were removed using the BBMap (http://sourceforge.net/project/bbmap) with a reference human genome. Taxonomic features were obtained by using MetaPhlAn2 v.2.7,^69^ and functional features were obtained by using HUMAnN2 v.2.8.0.^70^ Finally, the resultant UniRef90 IDs were converted to the KOs. A total of 2,635,653,581 reads (median 6,837,236 reads per sample) were obtained from WMS analysis.

### Quantification of total bacterial amounts

The relative amounts of bacteria in fecal samples were estimated by quantitative real-time PCR based on the 16S rRNA gene. The 16S rRNA gene was amplified with the primer 340 F (5′-TCC TAC GGG AGG CAG CAG-3′) and 518 R (5′-ATT ACC GCG GCT GCT GG-3′) using a Thermal Cycle Dice Real-Time System III (TaKaRa Bio, Inc.).

Each sample was measured in triplicates in a 25-μL reaction containing 12.5 μL of TB Green Premix Ex Taq (Tli RNaseH Plus) (Cat #RR820B, TaKaRa Bio, Inc.), 2 μM of each primer, and 1 μL of DNA template (a 10-fold dilution series of sample DNA) with following amplification conditions: 95°C for 30s, followed by 40 cycles of denaturation at 95°C for 5 s and annealing at 60°C for 30s. We quantified the bacterial amount by comparing threshold cycles (Ct) values to a standard curve generated from parallel reactions of serial dilutions (1 × 10^1^–1 × 10^7^) of the 16S rRNA gene from the *Escherichia coli* K12 w3110 strain. Regression coefficients (r^2^) for all standard curves were ≥ 0.98.

### SCFAs analysis

Fecal SCFAs were extracted with modification of previously described methods.^71,72^ Acetic acid (Cat #338826, Sigma-Aldrich, St. Louis, MO, USA), propionic acid (Cat #81910, Sigma), iso-butyric acid (Cat #58360, Sigma), butyric acid (Cat #W222100, Sigma), iso-valeric acid (Cat #129542, Sigma), and valeric acid (Cat # 240370, Sigma) were used as standard compounds. 4-methlyvaleric acid (Cat #277827, Sigma) was used as an internal standard (IS) for final adjustment on the quantity of each compound in samples. Briefly, 20 mg of fecal sample was suspended in extraction solution (450 μL of methylene chloride, 5 μL of IS (10 ppm), and 25 μL of 0.6 M HCl). The suspension was derivatized for 10 min after vortexing and centrifuging (14,000 × *g*) for 10 min at 4°C. Finally, the methylene chloride layer was transferred into the test vial and briefly vortexed before injecting into the gas chromatography-mass spectrometry (GC-MS).

The GC-MS analysis was performed on an Agilent 7890A gas chromatograph coupled to an Agilent 7000 triple quad mass spectrometric, detector (MSD, Cat #G7000-90038, Agilent Technologies). Derivatives were separated using a VF-WAXms capillary column (30 m × 0.25 mm, 0.25 μm film thickness, Agilent J & W Scientific, Folsom, CA, USA) with a carrier gas (helium) at a flow rate of 2.5 mL/min. One microliter of the sample was injected into the system. The oven program was set at 80°C for 2 min, raised to 160°C at a rate of 20°C/min, to 180°C at a rate of 3°C/min, to a final temperature of 230°C at a rate of 10°C/min and maintained for 5 min. The transfer line temperature was set to 200°C. The MS source was held at 230°C and the quadrupole at 150°C. The energy of electron ionization was set to 70 eV. The mass spectral data were collected in a selected ion monitoring mode.

Quantitative analysis software (MSD, Agilent Technologies) was used to process the GC-MS data for peak picking, standard curve construction, and SCFAs quantification. The concentration of each SCFA in samples was calculated using the calibration curve constructed from the GC-MS data of corresponding SCFA standards.

### Predictive SCFAs metabolite profiling

We used the Model-based Genomically Informed High-dimensional Predictor of Microbial Community Metabolic Profiles (MelonnPan v.0.99.0)^73^ to predict SCFAs profiles based on measured SCFAs in fecal samples and functional gene features of gut microbiomes obtained from HUMAnN2 (UniRef90 gene families).

After normalizing the raw measures into relative abundances, we filtered features (gene families and SCFAs) by both relative abundance (> 0.01%) and prevalence (> 10% of the samples). We measured SCFAs and functional gene features from 41 samples to construct a training set. SCFAs were predicted based on the functional gene features of 302 samples using elastic net regularization and cross-validation. The predictability of each metabolite was evaluated using the Spearman correlation coefficient (r) between the measured and predicted metabolites concentration across samples.

The same labels were repeatedly shuffled in both metabolite and gene family tables to test the significance of well-predicted metabolites (r > 0.3),^73^ the MelonnPan model was applied to the randomized data by shuffling to link genes to metabolites, and the predicted metabolites in the original data and randomized data were compared.

Butyrate and propionate were successfully predicted after evaluating 10-fold cross-validation of predicted metabolisms in the present study. The accuracy of predicted metabolites was evaluated by the representative training sample index (RTSI) scores based on principal component analysis (PCA).

### Animal studies

Female C57BL/6 mice were purchased from ORIENT Bio Korea. The mice (8-week-old) were divided into AD (n = 8) and non-AD (n = 10) groups. AD was induced for five weeks. Briefly, 200 μL of 1% 1-Chloro-2,4-dinitrobenzene (DNCB) in an acetone:olive oil mixture (3:1 vol/vol) was applied to the shaved dorsal skin, and then the skin was covered with a transparent film dressing. 1% DNCB was applied three times a week for one week. Then, 0.4% DNCB was applied three times a week for four weeks. At 35 days, mice were sacrificed, and the middle part of the colon was collected for gene expression analysis, and fecal samples were collected for microbiota and SCFA analyses. All procedures were performed in accordance with the guideline of the Institutional Animal Care and Usage Committee at Soonchunhyang University.

#### Quantitative reverse transcriptase-polymerase chain reaction analysis

Total RNA was extracted using TRIzol reagent (Cat #15596018, Invitrogen) from the collected middle part of the colon. RNA was converted to cDNA using reverse transcription reagents (Cat #FSQ-201, Toyobo). PCR was performed using SYBR Green Real time PCR Master Mix Kit (Toyobo) with specific primers for target genes (Table S12). The reaction was performed with QuantStudio5 Real-Time PCR System (Applied Biosystems, Foster City, CA, USA). The expression levels of target genes were calculated by comparing the relative expression levels after normalization to *β-actin*.

#### Microbiota analysis in mice fecal samples

Metagenomic DNA was extracted from fecal samples of mice using the RNeasy PowerMicrobiome Kit (Qiagen). The 16S rRNA gene amplicon sequencing and analyses were performed as described above. A total of 442,006 reads (median 20,543 reads per sample) in mouse fecal samples were obtained from sequence analyses.

#### SCFAs analysis

SCFAs in mice fecal samples were extracted and analyzed using GC-MS as described previously.

### *In vitro* cell experiment

Caco-2 cells (ATCC® CRL-1790^TM^) were cultured in Eagle’s minimum essential medium (EMEM) supplemented with 20% fetal bovine serum (FBS) and 1% penicillin-streptomycin by incubation at 37°C in a humidified atmosphere containing 5% CO_2_. Cultured Caco-2 cells at passages 20–27 were used for experiments.

#### Cell viability

Caco-2 cells were seeded (0.5 ×10^4^ cells/well) into 96-well plates and incubated at 37°C for 24 h to allow the cells to attach. Caco-2 cells were treated with butyrate (concentrations of 0.5, 1, and 5 mM). Afterward, 20 μL of MTT solution (final concentration of 1 mg/mL) was added to each well, and cells were incubated for 2 h. The cell culture medium was subsequently removed, and each well was treated with 200 μL DMSO to dissolve formazan crystals. Dissolved formazan absorbance was measured at 570 nm using a microplate reader (Sunrise-Basic Tecan, Tecan Austria GmbH, Grödig, Austria). The cell viability was expressed as the percentage of MTT reduction calculated relative to the absorbance of control cells.

#### Mitochondrial Function

The effect of butyrate on mitochondrial function was measured by seeding Caco-2 cells in Seahorse XFp mini-plate (Cat #103025-100, Agilent Technologies) at 0.7 ×10^4^ cells/well. Cells were cultured as described above. The cells were washed with extracellular flux (XF) DMEM medium (Agilent Technologies) supplemented with 5.55 mM glucose, 2 mM glutamine, and 1 mM sodium pyruvate followed by incubation in this medium for 60 min at 37°C in a non-CO_2_ incubator. Plates were transferred to a Seahorse XFp analyzer (Agilent Technologies) and subjected to an equilibration period. After measuring the basal oxygen consumption rate (OCR) for four cycles, oligomycin (1.5 µM, Agilent Technologies) was added to determine the proportion of respiration used to generate ATP. Then, carbonyl cyanide-4-(trifluoro methoxy) phenyl hydrazone (FCCP, 0.5 µM, Agilent Technologies) was added to determine the maximal respiration by mitochondria. After that, rotenone (0.5 µM, Agilent Technologies) and antimycin A (0.5 µM, Agilent Technologies) were added to measure the non-mitochondrial respiratory rate.

### Statistical analysis

Statistical analysis was performed with the R software v.4.0.2. All statistical tests for microbiome data were two-sided.

#### Clinical data

The Kruskal–Wallis test was used to compare continuous variables among groups, and the Chi-square test was used to compare categorical variables between groups. The ORs and corresponding 95% confidence intervals (CI) were calculated using unconditional logistic regression models.

#### Microbiota analysis

The difference in Shannon diversity index between non-AD and AD groups was analyzed using the Mann–Whitney–Wilcox test. Differences in beta-diversity were visualized using NMDS plots and tested for inference by PERMANOVA (Adonis from the package vegan with 999 permutations) based on the Bray–Curtis distance.

Differences in relative abundances in microbiota were analyzed using the Kruskal–Wallis tests for three-group comparisons, and Dunn’s test was used to identify pairwise differences between groups. Dunn’s test was performed using the ‘dunn.test’ package in R, and an approximately normal distribution was used to calculate *p-*values. *P-*values were adjusted using Benjamini–Hochberg false discovery rate (FDR) multiple testing, correction. Results with *q* (adjusted *p-*value) < 0.05 were considered statistically significant. WMS data was normalized using the cumulative sum scaling (CSS) method. Differences in normalized abundance by CSS among groups were analyzed using multivariate association with linear models (MaAsLin2).^74^

The inter-individual gut microbiota heterogeneity within the same group according to age was determined by the locally weighted scatterplot smoothing (LOESS) regression based on the Bray–Curtis dissimilarity. The heterogeneity was evaluated using the Kruskal–Wallis test and Mann–Whitney U test. The significance of quantitative real-time PCR results among groups was calculated using the Mann–Whitney U test and Kruskal–Wallis test.

The correlations between significantly associated genera with *Pparg, Gpr109a*, acetate, and butyrate in mice experiments were analyzed using the Spearman correlation in R software.

#### DMM clustering

The variance of the gut microbiota in all samples was analyzed using DMM modeling^75^ with the R package DirichletMultinomial. DMM clustering was conducted at the genus level to compare results between 16S and WMS. The lowest Laplace approximation score determined the number of clusters.

#### Comparison of taxonomic profiles between 16S and WMS

The overall concordance of microbiota compositions between 16S and WMS analyses was compared at the phylum and genus levels. Relative abundances of taxa were visualized using bar plots. Linear regression models and a Pearson correlation test were used to investigate the coefficient inference associations between 16S and WMS datasets. The correlation and regression of two datasets were visualized using scatterplots.

#### Indicator species analysis

The indicator species at each age were determined using the ‘*multipatt*’ function of the indicspecies package in R software. We selected species with > 10% prevalence and > 0.01% mean relative abundance in at least one group to identify indicators. This procedure is a statistical method determining whether a particular species is significantly more abundant in predefined groups than when the same species are randomly assigned to the groups. The non-AD microbiota determined the indicator species at each age. The significance of the association of the indicator with age was tested using 10,000 permutations. Results with *p* < .05 were considered indicators at each age. Resultant indicators were evaluated by multivariate analysis of MaAsLin2 after adjustment for covariates.

#### EnvFit analysis

Covariates (sex, delivery mode, feeding type, allergic family history, AD diagnosis, total IgE, egg-IgE, and milk-IgE) could be related to differences in the gut microbiome and allergic disease. The effect size and significance of each covariate in the variation of microbiota were determined using the ‘*envfit*’ function in R package vegan (v.2.5–7), which compared the difference in centroids of each group relative to the total variation. The significance was determined using 999 permutations. A PERMANOVA was used to determine the covariates with the strongest effects on taxonomic and functional features. Results with *p* < .05 were considered statistically significant.

#### Random forest model

Random forest regression was performed to model the gut microbiota age based on the relative abundance of bacterial species obtained from WMS of 112 non-AD samples (6–36 months) using the R package Ranger. The chronological age of the microbiota in children without AD was used as a training set to predict the estimated microbiota age (EMA). The resulting prediction model, defined by alterations in bacterial species, was subsequently applied to all subjects with Ranger’s ‘predict’ function. These estimates were used as a proxy for gut microbiota maturation.^76^ Next, sensitivity analyses were performed by restricting the models to samples not included in model building to confirm the independence of the training sets.

To estimate the microbiota age, the N top discriminatory species were identified by the ‘*rfcv*’ function in the randomForest package. We calculated the MAZ to compare the maturation of the gut microbiota among groups as described previously.^20,76^ A lower MAZ is indicative of a delay in microbiota development.

We used a similar process to estimate the SAZ for analyzing the dysbiosis of SCFAs profiles according to gut microbiota development variations between non-AD and AD groups, and predicted the SCFA age score using the training set. Linear regression models and a Pearson correlation test were used to analyze the associations between the SAZ and MAZ. In addition, Spearman correlation was used to identify the species associated with SAZ in each phenotypic group.

The correlations between significantly associated species with abnormal MAZ or SAZ in AD groups and clinical features (SCORAD index, Total IgE, egg-IgE, milk-IgE, and eosinophil) were analyzed using the Spearman correlation in R software.

#### Network analysis

Interspecies correlations were estimated using FastSpar^77^ based on Pearson correlation with 1,000 bootstraps. Pseudo *p*-values were calculated as the proportion of simulated bootstrapped datasets with a correlation at least as extreme as the one computed for the original data set. The corresponding correlation network was visualized using the R package qgraph. The top 10 species in each microbiota (GMT) group were selected for each phenotype by random forest, and they were used for network analysis. Significant correlations (with *p* < .05) were shown in the network.

Node sizes were scaled on the eigenvector centrality measure, which was determined using the R package igraph. Edge thickness denoted a FastSpar correlation ranging from values −0.4 to 0.4. Hubs were identified using the PageRank algorithm, a link analysis with the underlying assumption that hubs are more connected to other nodes than non-hub nodes.^78^ The top five species with the highest PageRank in each phenotypic group were selected as hubs in the networks. This process was performed in R using the igraph package.

#### Functional features analysis

Functional features of the gut microbiome were analyzed using the KO category. The significantly different features, according to age, were selected by MaAsLin2 after adjustment for covariates (*q* < 0.05). The N top discriminatory KOs were selected by ‘*rfcv*’ function in the randomForest package.

Gene families involved in butyrate and propionate metabolism were compared between phenotypic groups at each age. MaAsLin2 evaluated the difference in gene families between phenotypic groups at each age after adjusting for covariates. We evaluated the significance of different KOs and gene families between phenotypic groups at each age using the Kruskal–Wallis test and Dunn’s test. *P*-values were adjusted using the Benjamini–Hochberg method. Results with *q* < 0.05 were considered statistically significant.

## Supplementary Material

Supplemental Material

## Data Availability

All sequence data (16S and WMS) obtained from this study are available in the EMBL SRA database under the study numbers PRJEB41351 (http://www.ebi.ac.uk/ena/data/view/PRJEB41351), PRJEB45443 (http://www.ebi.ac.uk/ena/data/view/PRJEB45443) and PRJEB47988 (http://www.ebi.ac.uk/ena/data/view/PRJEB47988). Measured concentrations of SCFA data are deposited on Zenodo at https://doi.org/10.5281/zenodo.5602428.

## Abbreviations

AD, atopic dermatitis; SCFA, short-chain fatty acid; SCORAD, scoring of atopic dermatitis; COCOA, the Longitudinal Cohort for Childhood Origin of Asthma and Allergic Disease birth cohort; 16S, 16S rRNA amplicon sequences; WMS, whole metagenome sequences; RTP, quantitative real-time PCR; DMM, Dirichlet multinomial mixture; GMT, gut microbiota type; OR, odds ratio; NMDS, non-metric multidimensional scaling; UC, unclassified; EBF, exclusive breastfeeding; MAZ, microbial-by-age z-score; SAZ, SCFA-by-age z-score; KO, KEGG Orthology; OCR, oxygen consumption rate; GC-MS, gas chromatography-mass spectrometry

## References

[1] Zhou H, Sun L, Zhang S, Zhao X, Gang X, Wang G. The crucial role of early-life gut microbiota in the development of type 1 diabetes. Acta Diabetol. 2021;58(3):249–23. doi:10.1007/s00592-020-01563-z. 32712802

[2] Stewart CJ, Ajami NJ, O’Brien JL, Hutchinson DS, Smith DP, Wong MC, Ross MC, Lloyd RE, Doddapaneni H, Metcalf GA, et al. Temporal development of the gut microbiome in early childhood from the TEDDY study. Nature. 2018;562(7728):583–588. doi:10.1038/s41586-018-0617-x. 30356187 PMC6415775

[3] Vatanen T, Plichta DR, Somani J, Münch PC, Arthur TD, Hall AB, Rudolf S, Oakeley EJ, Ke X, Young RA, et al. Genomic variation and strain-specific functional adaptation in the human gut microbiome during early life. Nat Microbiol. 2018;4(3):470–479. doi:10.1038/s41564-018-0321-5. 30559407 PMC6384140

[4] Niu J, Xu L, Qian Y, Sun Z, Yu D, Huang J, Zhou X, Wang Y, Zhang T, Ren R, et al. Evolution of the gut microbiome in early childhood: a cross-sectional study of Chinese children. Front Microbiol. 2020;11:439. doi:10.3389/fmicb.2020.00439. 32346375 PMC7169428

[5] Stefka AT, Feehley T, Tripathi P, Qiu J, McCoy K, Mazmanian SK, Tjota MY, Seo GY, Cao S, Theriault BR, et al. Commensal bacteria protect against food allergen sensitization. Proc Natl Acad Sci U S A. 2014;111(36):13145–13150. doi:10.1073/pnas.1412008111. 25157157 PMC4246970

[6] Thorburn AN, McKenzie CI, Shen S, Stanley D, Macia L, Mason LJ, Roberts LK, Wong CH, Shim R, Robert R, et al. Evidence that asthma is a developmental origin disease influenced by maternal diet and bacterial metabolites. Nat Commun. 2015;6(1):7320. doi:10.1038/ncomms8320. 26102221

[7] Simonyte Sjodin K, Hammarstrom ML, Ryden P, Sjodin A, Hernell O, Engstrand L, West CE. Temporal and long-term gut microbiota variation in allergic disease: a prospective study from infancy to school age. Allergy. 2019;74(1):176–185. doi:10.1111/all.13485. 29786876

[8] Fujimura KE, Sitarik AR, Havstad S, Lin DL, Levan S, Fadrosh D, Panzer AR, LaMere B, Rackaityte E, Lukacs NW, et al. Neonatal gut microbiota associates with childhood multisensitized atopy and T cell differentiation. Nat Med. 2016;22(10):1187–1191. doi:10.1038/nm.4176. 27618652 PMC5053876

[9] Geuking MB, Cahenzli J, Lawson MA, Ng DC, Slack E, Hapfelmeier S, McCoy KD, Macpherson AJ. Intestinal bacterial colonization induces mutualistic regulatory T cell responses. Immunity. 2011;34(5):794–806. doi:10.1016/j.immuni.2011.03.021. 21596591

[10] Furusawa Y, Obata Y, Fukuda S, Endo TA, Nakato G, Takahashi D, Nakanishi Y, Uetake C, Kato K, Kato T, et al. Commensal microbe-derived butyrate induces the differentiation of colonic regulatory T cells. Nature. 2013;504(7480):446–450. doi:10.1038/nature12721. 24226770

[11] Tanaka M, Nakayama J. Development of the gut microbiota in infancy and its impact on health in later life. Allergol Int. 2017;66(4):515–522. doi:10.1016/j.alit.2017.07.010. 28826938

[12] Penders J, Gerhold K, Stobberingh EE, Thijs C, Zimmermann K, Lau S, Hamelmann E. Establishment of the intestinal microbiota and its role for atopic dermatitis in early childhood. J Allergy Clin Immunol. 2013;132(3):601–607. doi:10.1016/j.jaci.2013.05.043. 23900058

[13] Lee MJ, Kang MJ, Lee SY, Lee E, Kim K, Won S, Suh DI, Kim KW, Sheen YH, Ahn K, et al. Perturbations of gut microbiome genes in infants with atopic dermatitis according to feeding type. J Allergy Clin Immunol. 2018;141(4):1310–1319. doi:10.1016/j.jaci.2017.11.045. 29339259

[14] Song H, Yoo Y, Hwang J, Na YC, Kim HS. *Faecalibacterium prausnitzii* subspecies–level dysbiosis in the human gut microbiome underlying atopic dermatitis. J Allergy Clin Immunol. 2016;137(3):852–860. doi:10.1016/j.jaci.2015.08.021. 26431583

[15] Paller AS, Kong HH, Seed P, Naik S, Scharschmidt TC, Gallo RL, Luger T, Irvine AD. The microbiome in patients with atopic dermatitis. J Allergy Clin Immunol. 2019;143(1):26–35. doi:10.1016/j.jaci.2018.11.015. 30476499 PMC7163929

[16] Abrahamsson TR, Jakobsson HE, Andersson AF, Bjorksten B, Engstrand L, Jenmalm MC. Low diversity of the gut microbiota in infants with atopic eczema. J Allergy Clin Immunol. 2012;129(2):434–440. doi:10.1016/j.jaci.2011.10.025. 22153774

[17] Nylund L, Nermes M, Isolauri E, Salminen S, de Vos Wm, Satokari R, de Vos WM. Severity of atopic disease inversely correlates with intestinal microbiota diversity and butyrate-producing bacteria. Allergy. 2015;70(2):241–244. doi:10.1111/all.12549. 25413686

[18] Morrison DJ, Preston T. Formation of short chain fatty acids by the gut microbiota and their impact on human metabolism. Gut Microbes. 2016;7(3):189–200. doi:10.1080/19490976.2015.1134082. 26963409 PMC4939913

[19] Bokulich NA, Chung J, Battaglia T, Henderson N, Jay M, Li H, DL A, Wu F, Perez-Perez GI, Chen Y, et al. Antibiotics, birth mode, and diet shape microbiome maturation during early life. Sci Transl Med. 2016;8(343):343ra82. doi:10.1126/scitranslmed.aad7121. PMC5308924 27306664

[20] Galazzo G, van Best N, Bervoets L, Dapaah IO, Savelkoul PH, Hornef MW, Lau, Consortium G-m E, Lau S, Hamelmann E, et al. Development of the microbiota and associations with birth mode, diet, and atopic disorders in a longitudinal analysis of stool samples, collected from infancy through early childhood. Gastroenterology. 2020;158(6):1584–1596. doi:10.1053/j.gastro.2020.01.024. 31958431

[21] Franzosa EA, Hsu T, Sirota-Madi A, Shafquat A, Abu-Ali G, Morgan XC, Huttenhower C. Sequencing and beyond: integrating molecular ‘omics’ for microbial community profiling. Nat Rev Microbiol. 2015;13(6):360–372. doi:10.1038/nrmicro3451. 25915636 PMC4800835

[22] McFall-Ngai M, Hadfield MG, Bosch TC, Carey HV, Domazet-Loso T, Douglas AE, Dubilier N, Eberl G, Fukami T, Gilbert SF, et al. Animals in a bacterial world, a new imperative for the life sciences. Proc Natl Acad Sci U S A. 2013;110(9):3229–3236. doi:10.1073/pnas.1218525110. 23391737 PMC3587249

[23] Lynch SV, Pedersen O, Phimister EG. The human intestinal microbiome in health and disease. N Engl J Med. 2016;375(24):2369–2379. doi:10.1056/NEJMra1600266. 27974040

[24] Thompson AL, Monteagudo-Mera A, Cadenas MB, Lampl ML, Azcarate-Peril MA. Milk- and solid-feeding practices and daycare attendance are associated with differences in bacterial diversity, predominant communities, and metabolic and immune function of the infant gut microbiome. Front Cell Infect Microbiol. 2015;5:3. doi:10.3389/fcimb.2015.00003. 25705611 PMC4318912

[25] Ismail IH, Oppedisano F, Joseph SJ, Boyle RJ, Licciardi PV, Robins-Browne RM, Tang ML. Reduced gut microbial diversity in early life is associated with later development of eczema but not atopy in high-risk infants. Pediatr Allergy Immunol. 2012;23(7):674–681. doi:10.1053/j.gastro.2020.01.024. 22831283

[26] Zimmermann P, Messina N, Mohn WW, Finlay BB, Curtis N. Association between the intestinal microbiota and allergic sensitization, eczema, and asthma: a systematic review. J Allergy Clin Immunol. 2019;143(2):467–485. doi:10.1016/j.jaci.2018.09.025. 30600099

[27] Zheng H, Liang H, Wang Y, Miao M, Shi T, Yang F, Liu E, Yuan W, Ji ZS, Li DK. Altered gut microbiota composition associated with eczema in infants. PLoS One. 2016;11(11):e0166026. doi:10.1371/journal.pone.0166026. 27812181 PMC5094743

[28] Reddel S, Del Chierico F, Quagliariello A, Giancristoforo S, Vernocchi P, Russo A, Fiocchi A, Rossi P, Putignani L, El Hachem M. Gut microbiota profile in children affected by atopic dermatitis and evaluation of intestinal persistence of a probiotic mixture. Sci Rep. 2019;9:4996. doi:10.1038/s41598-019-41149-6. 30899033 PMC6428866

[29] Arrieta MC, Stiemsma LT, Dimitriu PA, Thorson L, Russell S, Yurist-Doutsch S, Kuzeljevic B, Gold MJ, Britton HM, Lefebvre DL, et al. Early infancy microbial and metabolic alterations affect risk of childhood asthma. Sci Transl Med. 2015;7(307):307ra152. doi:10.1126/scitranslmed.aab2271. 26424567

[30] Depner M, Taft DH, Kirjavainen PV, Kalanetra KM, Karvonen AM, Peschel S, Schmausser-Hechfellner E, Roduit C, Frei R, Lauener R, et al. Maturation of the gut microbiome during the first year of life contributes to the protective farm effect on childhood asthma. Nat Med. 2020;26(11):1766–1775. doi:10.1038/s41591-020-1095-x. 33139948

[31] LDH T, Chan JCY, Yap GC, Purbojati RW, Drautz-Moses DI, Koh YM, Tay CJX, Huang CH, Kioh DYQ, Woon JY, et al. A compromised developmental trajectory of the infant gut microbiome and metabolome in atopic eczema. Gut Microbes. 2020;12(1):1–22. doi:10.1080/19490976.2020.1801964. PMC7553750 33023370

[32] Fukami T. Historical contigency in community assembly: integrating niches, species pools, and priority effects. Annu Rev Ecol Evol Syst. 2015;46(1):1–23. doi:10.1146/annurev-ecolsys-110411-160340.

[33] Roduit C, Frei R, Ferstl R, Loeliger S, Westermann P, Rhyner C, Schiavi E, Barcik W, Rodriguez-Perez N, Wawrzyniak M, et al. High levels of butyrate and propionate in early life are associated with protection against atopy. Allergy. 2019;74(4):799–809. doi:10.1111/all.13660. 30390309

[34] Wopereis H, Sim K, Shaw A, Warner JO, Knol J, Kroll JS. Intestinal microbiota in infants at high risk for allergy: effects of prebiotics and role in eczema development. J Allergy Clin Immunol. 2018;141(4):1334–1342. doi:10.1016/j.jaci.2017.05.054. 28866384

[35] Yatsunenko T, Rey FE, Manary MJ, Trehan I, Dominguez-Bello MG, Contreras M, Magris M, Hidalgo G, Baldassano RN, Anokhin AP, et al. Human gut microbiome viewed across age and geography. Nature. 2012;486(7402):222–227. doi:10.1038/nature11053. 22699611 PMC3376388

[36] Penders J, Thijs C, Vink C, Stelma FF, Snijders B, Kummeling I, van den Brandt Pa, Stobberingh EE, van den Brandt PA. Factors influencing the composition of the intestinal microbiota in early infancy. Pediatrics. 2006;118(2):511–521. doi:10.1542/peds.2005-2824. 16882802

[37] Campana R, Dzoro S, Mittermann I, Fedenko E, Elisyutina O, Khaitov M, Karaulov A, Valenta R. Molecular aspects of allergens in atopic dermatitis. Curr Opin Allergy Clin Immunol. 2017;17(4):269–277. doi:10.1097/ACI.0000000000000378. 28622169 PMC6392175

[38] Wollenberg A, Thomsen SF, Lacour JP, Jaumont X, Lazarewicz S. Targeting immunoglobulin E in atopic dermatitis: a review of the existing evidence. World Allergy Organ J. 2021;14(3):100519. doi:10.1016/j.waojou.2021.100519. 33815652 PMC8005850

[39] Los-Rycharska E, Golebiewski M, Sikora M, Grzybowski T, Gorzkiewicz M, Popielarz M, Gawryjolek J, Krogulska A. A combined analysis of gut and skin microbiota in infants with food allergy and atopic dermatitis: a pilot study. Nutrients. 2021;13(5):1682. doi:10.3390/nu13051682. 34063398 PMC8156695

[40] Troy EB. Beneficial effects of *Bacteroides fragilis* polysaccharides on the immune system. Front Biosci (Landmark Ed). 2010;15(1):25–34. doi:10.2741/3603. 20036803 PMC2995369

[41] Chen HH, Chen DY, Chao YH, Chen YM, Wu CL, Lai KL, Lin CH, Lin CC. Acarbose decreases the rheumatoid arthritis risk of diabetic patients and attenuates the incidence and severity of collagen-induced arthritis in mice. Sci Rep. 2015;5(1):18288. doi:10.1038/srep18288. 26678745 PMC4683371

[42] Docena G, Rovedatti L, Kruidenier L, Fanning A, Leakey NA, Knowles CH, Lee K, Shanahan F, Nally K, McLean PG, et al. Down-regulation of p38 mitogen-activated protein kinase activation and proinflammatory cytokine production by mitogen-activated protein kinase inhibitors in inflammatory bowel disease. Clin Exp Immunol. 2010;162(1):108–115. doi:10.1111/j.1365-2249.2010.04203.x. 20731675 PMC2990936

[43] Raza A, Crothers JW, McGill MM, Mawe GM, Teuscher C, Krementsov DN. Anti-inflammatory roles of p38alpha MAPK in macrophages are context dependent and require IL-10. J Leukoc Biol. 2017;102(5):1219–1227. doi:10.1189/jlb.2AB0116-009RR. 28877953 PMC6608039

[44] Thangaraju M, Cresci GA, Liu K, Ananth S, Gnanaprakasam JP, Browning DD, Mellinger JD, Smith SB, Digby GJ, Lambert NA, et al. GPR109A is a G-protein-coupled receptor for the bacterial fermentation product butyrate and functions as a tumor suppressor in colon. Cancer Res. 2009;69(7):2826–2832. doi:10.1158/0008-5472.CAN-08-4466. 19276343 PMC3747834

[45] Tylichova Z, Strakova N, Vondracek J, Vaculova AH, Kozubik A, Hofmanova J. Activation of autophagy and PPARgamma protect colon cancer cells against apoptosis induced by interactive effects of butyrate and DHA in a cell type-dependent manner: the role of cell differentiation. J Nutr Biochem. 2017;39:145–155. doi:10.1016/j.jnutbio.2016.09.006. 27840291

[46] Alex S, Lange K, Amolo T, Grinstead JS, Haakonsson AK, Szalowska E, Koppen A, Mudde K, Haenen D, Al-Lahham S, et al. Short-chain fatty acids stimulate angiopoietin-like 4 synthesis in human colon adenocarcinoma cells by activating peroxisome proliferator-activated receptor gamma. Mol Cell Biol. 2013;33(7):1303–1316. doi:10.1128/MCB.00858-12. 23339868 PMC3624264

[47] Hernandez-Quiles M, Broekema MF, Kalkhoven E. PPARgamma in metabolism, immunity, and cancer: unified and diverse mechanisms of action. Front Endocrinol (Lausanne). 2021;12:624112. doi:10.3389/fendo.2021.624112. 33716977 PMC7953066

[48] Mookerjee SA, Gerencser AA, Nicholls DG, Brand MD. Quantifying intracellular rates of glycolytic and oxidative ATP production and consumption using extracellular flux measurements. J Biol Chem. 2017;292(17):7189–7207. doi:10.1074/jbc.M116.774471. 28270511 PMC5409486

[49] Mookerjee SA, Goncalves RLS, Gerencser AA, Nicholls DG, Brand MD. The contributions of respiration and glycolysis to extracellular acid production. Biochim Biophys Acta. 2015;1847(2):171–181. doi:10.1016/j.bbabio.2014.10.005. 25449966

[50] Gillis CC, Hughes ER, Spiga L, Winter MG, Zhu W, Furtado de Carvalho T, Chanin RB, Behrendt CL, Hooper LV, Santos RL, et al. Dysbiosis-associated change in host metabolism generates lactate to support *Salmonella* growth. Cell Host Microbe. 2018;23(1):54–64. doi:10.1016/j.chom.2017.11.006. 29276172 PMC5764812

[51] Geeraerts X, Bolli E, Fendt SM, Van Ginderachter JA. Macrophage metabolism as therapeutic target for cancer, atherosclerosis, and obesity. Front Immunol. 2017;8:289. doi:10.3389/fimmu.2017.00289. 28360914 PMC5350105

[52] Reese AT, Cho EH, Klitzman B, Nichols SP, Wisniewski NA, Villa MM, Durand HK, Jiang S, Midani FS, Nimmagadda SN, et al. Antibiotic-induced changes in the microbiota disrupt redox dynamics in the gut. Elife. 2018;7:e35987. doi:10.7554/eLife.35987. 29916366 PMC6008055

[53] Litvak Y, Byndloss MX, Tsolis RM, Baumler AJ. Dysbiotic Proteobacteria expansion: a microbial signature of epithelial dysfunction. Curr Opin Microbiol. 2017;39:1–6. doi:10.1016/j.mib.2017.07.003. 28783509

[54] Nguyen TL, Vieira-Silva S, Liston A, Raes J. How informative is the mouse for human gut microbiota research? Dis Model Mech. 2015;8(1):1–16. doi:10.1242/dmm.017400. 25561744 PMC4283646

[55] Park JC, Im SH. Of men in mice: the development and application of a humanized gnotobiotic mouse model for microbiome therapeutics. Exp Mol Med. 2020;52(9):1383–1396. doi:10.1038/s12276-020-0473-2. 32908211 PMC8080820

[56] Kelly BJ, Gross R, Bittinger K, Sherrill-Mix S, Lewis JD, Collman RG, Bushman FD, Li H. Power and sample-size estimation for microbiome studies using pairwise distances and PERMANOVA. Bioinformatics. 2015;31(15):2461–2468. doi:10.1093/bioinformatics/btv183. 25819674 PMC4514928

[57] Yang HJ, Lee SY, Suh DI, Shin YH, Kim BJ, Seo JH, Chang HY, Kim KW, Ahn K, Shin YJ, et al. The Cohort for Childhood Origin of Asthma and allergic diseases (COCOA) study design, rationale and methods. BMC Pulm Med. 2014;14(1):109. doi:10.1186/1471-2466-14-109. 24990471 PMC4099383

[58] Hanifin JM, Rajka G. Diagnostic features of atopic dermatitis. Acta Derm Venereol Suppl (Stockh). 1980;92:44–47. doi:10.2340/00015555924447.

[59] Wollenberg A, Barbarot S, Bieber T, Christen-Zaech S, Deleuran M, Fink-Wagner A, Gieler U, Girolomoni G, Lau S, Muraro A, et al. Consensus-based European guidelines for treatment of atopic eczema (atopic dermatitis) in adults and children: part II. J Eur Acad Dermatol Venereol. 2018;32(6):850–878. doi:10.1111/jdv.14891. 29878606

[60] Park JU, Oh B, Lee JP, Choi MH, Lee MJ, Kim BS. Influence of microbiota on diabetic foot wound in comparison with adjacent normal skin based on the clinical features. Biomed Res Int. 2019;2019:7459236. doi:10.1155/2019/7459236. 31531366 PMC6720033

[61] Park JI, Kim TY, Oh B, Cho H, Kim JE, Yoo SH, Lee JP, Kim YS, Chun J, Kim BS, et al. Comparative analysis of the tonsillar microbiota in IgA nephropathy and other glomerular diseases. Sci Rep. 2020;10(1):16206. doi:10.1038/s41598-020-73035-x. 33004860 PMC7530979

[62] Salter SJ, Cox MJ, Turek EM, Calus ST, Cookson WO, Moffatt MF, Turner P, Parkhill J, Loman NJ, Walker AW. Reagent and laboratory contamination can critically impact sequence-based microbiome analyses. BMC Biol. 2014;12(1):87. doi:10.1186/s12915-014-0087-z. 25387460 PMC4228153

[63] Eisenhofer R, Minich JJ, Marotz C, Cooper A, Knight R, Weyrich LS. Contamination in low microbial biomass microbiome studies: issues and recommendations. Trends Microbiol. 2019;27(2):105–117. doi:10.1016/j.tim.2018.11.003. 30497919

[64] Bolyen E, Rideout JR, Dillon MR, Bokulich NA, Abnet CC, Al-Ghalith GA, Alexander H, Alm EJ, Arumugam M, Asnicar F, et al. Reproducible, interactive, scalable and extensible microbiome data science using QIIME 2. Nat Biotechnol. 2019;37(8):852–857. doi:10.1038/s41587-019-0209-9. 31341288 PMC7015180

[65] Kim OS, Cho YJ, Lee K, Yoon SH, Kim M, Na H, Park SC, Jeon YS, Lee JH, Yi H, et al. Introducing EzTaxon-e: a prokaryotic 16S rRNA gene sequence database with phylotypes that represent uncultured species. Int J Syst Evol Microbiol. 2012;62(Pt_3):716–721. doi:10.1099/ijs.0.038075-0. 22140171

[66] Lee JJ, Kim SH, Lee MJ, Kim BK, Song WJ, Park HW, Cho SH, Hong SJ, Chang YS, Kim BS. Different upper airway microbiome and their functional genes associated with asthma in young adults and elderly individuals. Allergy. 2019;74(4):709–719. doi:10.1111/all.13608. 30242844

[67] Bolger AM, Lohse M, Usadel B. Trimmomatic: a flexible trimmer for Illumina sequence data. Bioinformatics. 2014;30(15):2114–2120. doi:10.1093/bioinformatics/btu170. 24695404 PMC4103590

[68] Zhang J, Kobert K, Flouri T, Stamatakis A. PEAR: a fast and accurate Illumina Paired-End reAd mergeR. Bioinformatics. 2014;30(5):614–620. doi:10.1093/bioinformatics/btt593. 24142950 PMC3933873

[69] Segata N, Waldron L, Ballarini A, Narasimhan V, Jousson O, Huttenhower C. Metagenomic microbial community profiling using unique clade-specific marker genes. Nat Methods. 2012;9(8):811–814. doi:10.1038/nmeth.2066. 22688413 PMC3443552

[70] Franzosa EA, McIver LJ, Rahnavard G, Thompson LR, Schirmer M, Weingart G, Lipson KS, Knight R, Caporaso JG, Segata N, et al. Species-level functional profiling of metagenomes and metatranscriptomes. Nat Methods. 2018;15(11):962–968. doi:10.1038/s41592-018-0176-y. 30377376 PMC6235447

[71] Fava F, Gitau R, Griffin BA, Gibson GR, Tuohy KM, Lovegrove JA. The type and quantity of dietary fat and carbohydrate alter faecal microbiome and short-chain fatty acid excretion in a metabolic syndrome ‘at-risk’ population. Int J Obes (Lond). 2013;37(2):216–223. doi:10.1038/ijo.2012.33. 22410962

[72] Zhao G, Nyman M, Jonsson JA. Rapid determination of short-chain fatty acids in colonic contents and faeces of humans and rats by acidified water-extraction and direct-injection gas chromatography. Biomed Chromatogr. 2006;20(8):674–682. doi:10.1002/bmc.580. 16206138

[73] Mallick H, Franzosa EA, McLver LJ, Banerjee S, Sirota-Madi A, Kostic AD, Clish CB, Vlamakis H, Xavier RJ, Huttenhower C. Predictive metabolomic profiling of microbial communities using amplicon or metagenomic sequences. Nat Commun. 2019;10(1):3136. doi:10.1038/s41467-019-10927-1. 31316056 PMC6637180

[74] Mallick H, Rahnavard A, McLver LJ, Ma S, Zhang Y, Nguyen LH, Tickle TL, Weingart G, Ren B, Schwager EH, et al. Multivariable association discovery in population-scale meta-omics studies. PLoS Comput Biol. 2021;17(11):e1009442. doi:10.1371/journal.pcbi.1009442. 34784344 PMC8714082

[75] Holmes I, Harris K, Quince C, Gilbert JA. Dirichlet multinomial mixtures: generative models for microbial metagenomics. PLoS One. 2012;7(2):e30126. doi:10.1371/journal.pone.0030126. 22319561 PMC3272020

[76] Subramanian S, Huq S, Yatsunenko T, Haque R, Mahfuz M, Alam MA, Benezra A, DeStefano J, Meier MF, Muegge BD, et al. Persistent gut microbiota immaturity in malnourished Bangladeshi children. Nature. 2014;510(7505):417–421. doi:10.1038/nature13421. 24896187 PMC4189846

[77] Watts SC, Ritchie SC, Inouye M, Holt KE, Stegle O. FastSpar: rapid and scalable correlation estimation for compositional data. Bioinformatics. 2019;35(6):1064–1066. doi:10.1093/bioinformatics/bty734. 30169561 PMC6419895

[78] Tzartos SJ, Barkas T, Cung MT, Mamalaki A, Marraud M, Orlewski P, Papanastasiou D, Sakarellos C, Sakarellos-Daitsiotis M, Tsantili P, et al. Anatomy of the antigenic structure of a large membrane autoantigen, the muscle-type nicotinic acetylcholine receptor. Immunol Rev. 1998;163(1):89–120. doi:10.1111/j.1600-065x.1998.tb01190.x. 9700504

