# Supplementary material for: Disordered development of gut microbiome interferes with the establishment of the gut ecosystem during early childhood with atopic dermatitis
Source: Gut Microbes. 2022 Apr 29;14(1):2068366. doi: 10.1080/19490976.2022.2068366 (PMC9067516; doi:10.1080/19490976.2022.2068366)
Supplement: Supplemental Material [file KGMI_A_2068366_SM0654.pdf]

Supplementary materials

Supplementary Figure legends

Figure S1. Summary of sampling strategy in this study.

Figure S2. Microbiota detected in negative controls for 16S and WMS. For quality control of microbiome analyses, negative controls for the sampling tube, DNA extraction kit, and library preparation were sequenced and compared to the microbiota in fecal samples. (a) NMDS plots compare the microbiota between negative controls and fecal samples based on Bray–Curtis dissimilarity at the phylum and genus levels. (b) The relative abundances of dominant genera were obtained from the 16S dataset. The heatmap shows the difference in relative abundances among negative controls for the sampling tube (Blank tube), DNA extraction kit (Extraction kit), and amplicon library preparation (Library). The proportions of commonly detected genera in negative controls and fecal samples from non-AD were compared. (c) The comparison of relative abundances of dominant genera obtained from WMS. The heatmap shows the difference in relative abundances among fecal samples from non-AD (Samples), DNA extraction kit (Extraction kit), and whole metagenome library preparation (Library). *Propionibacterium* and *Burkholderia* were detected at high levels in the negative controls for WMS.

Figure S3. Comparison of the gut microbiota obtained from WMS between clusters determined by DMM modeling in each phenotypic group. The lowest Laplace approximation indicated the

number of clusters was two for the gut microbiota in all groups. Microbiota clusters in (a) non-AD, (b) mild AD, and (c) moderate to severe AD groups. The median age of GMT2 was higher than that of GMT1 in all groups. Differences of dominant genera between GMT1 and GMT2 were similar in all groups.

Figure S4. Comparison of taxonomic profiles between WMS and 16S datasets in each phenotypic group at the phylum and genus levels. The concordance of relative abundances between two datasets was determined by the linear regression model and Pearson correlation. (a) Phylum compositions in each phenotypic group were compared between two datasets. (b) Genus compositions in each phenotypic group were compared between two datasets. The correlation (R) values between WMS and 16S were higher at the phylum level than at the genus level.

Figure S5. Relative abundances of facultative anaerobes and strict anaerobes along with age and difference of species composition by significant covariates in Envfit model. (a) The relative abundances of facultative anaerobes and strict anaerobes were compared among groups. The relative abundances of facultative anaerobes were significantly different between phenotypic groups after 6 months. (b) Significantly different species between exclusive breastfeeding (EBF) infants and non-exclusive breastfeeding (Non-EBF) infants. Mod-Sev AD: moderate to severe AD. *$P < 0.05$, **$P < 0.01$.

Figure S6. Relative abundances of associated species with delayed MAZ score in AD groups and diversity obtained from 16S and functional gene datasets were compared among

phenotypic groups. (a) The relative abundances of associated species with delayed MAZ scores in AD groups were compared to those in non-AD group. The line plot indicated the shift of relative abundance (median value) for each species along with age. (b) Shannon diversity indices obtained from 16S and functional gene features were compared among phenotypic groups in each age. Mod-Sev AD: moderate to severe AD. *$P < 0.05$.

Figure S7. Network analysis of the top 10 species in each microbiota type (GMT group) for all subjects and AD groups. Positive correlations are marked by blue edges and negative correlations by red edges. Edge thickness denotes FastSpar correlation, ranging from value -0.4 to 0.4. Node sizes were scaled on the eigenvector centrality measure. Only significant correlations with $P < 0.05$ are shown. The top 5 network hubs determined by the PageRank algorithm are marked in bold with an orange square symbol.

Figure S8. Comparison of gene families involved in butyrate metabolism among phenotypic groups according to age. Statistical significance was calculated by the Kruskal–Wallis test and Dunn's test. *P*-value was adjusted by the Benjamini–Hochberg method. MaAsLin2 evaluated the difference in gene families between phenotypic groups after adjustment. Significantly different gene families were selected by the MaAsLin2 result and Dunn's test. Mod-Sev: moderate to severe AD. *$q < 0.05$, **$q < 0.01$, ***$q < 0.001$.

Figure S9. Comparison of species corresponding to significantly different gene families involved in butyrate metabolism between non-AD and AD groups. Statistical significance was calculated by the Kruskal-Wallis test and Dunn's test. *P*-value was adjusted by the Benjamini–

Hochberg method. Species with normalized abundance < 1% in each group were summed as others. Mod-Sev: moderate to severe AD. *$q < 0.05$, **$q < 0.01$.

Figure S10. Comparison of gene families involved in propionate metabolism among groups according to age. Statistical significance was calculated by the Kruskal–Wallis test and Dunn's test. *P*-value was adjusted by the Benjamini–Hochberg method. MaAsLin2 evaluated the differences in gene families between phenotypic groups after adjustment. Significantly different gene families were selected by the MaAsLin2 result and Dunn's test. Mod-Sev: moderate to severe AD. *$q < 0.05$, **$q < 0.01$.

Supplementary tables

Table S1. Simulated effect sizes (ω2) for PERMANOVA power of 80% and 90% with varied sample sizes. The micropower R-package was used to calculate the statistical power of sample sizes based on the weighted UniFrac distance of within-group distance variance with a significance level of 0.05.

Table S2. Clinical characteristics of the study subjects.

Table S3. Number of samples analyzed for each dataset at each age.

Table S4. Comparison of odds ratio (OR) for covariates between GMT1 and GMT2. CI: confidence interval.

Table S5. Mean relative abundance of top 20 genera between GMT1 and GMT2 determined by DMM modeling. We used the Benjamini–Hochberg method adjusted *p*-value to calculate the *q* value (false discovery rate adjusted *p*-value).

Table S6. Comparison of indicator species for each age among phenotypic groups. Indicator species were selected after prefiltering by > 10% of prevalence and > 0.01% relative abundance in at least one group.

Table S7. Indicator species at each time point were evaluated after adjusting sex, feeding type, AD phenotype, and family history using MaAsLin 2 ($q < 0.25$). The time point of 6 months was used as a reference for the analysis. The analyzed species were selected after prefiltering for > 10% of prevalence and > 0.01% relative abundance in at least one group. *P*-value was adjusted by the Benjamini–Hochberg method to calculate the *q* value (false discovery rate adjusted *p*-value).

Table S8. Correlation between covariates and microbiota variation. The correlations were determined using the EnvFit model based on Bray–Curtis dissimilarity in each dataset.

Table S9. Comparison of gene families involved in butyrate metabolism among phenotypic

groups at each age. Significantly different gene families were selected using the Dunn's post-hoc test and MaAsLin2 analysis. *P*-value was adjusted using the Benjamini–Hochberg method to calculate the *q* value (false discovery rate adjusted *p*-value). N: Non-AD, M: Mild AD, MS: Moderate to Severe AD.

Table S10. Comparison of gene families involved in propionate metabolism among phenotypic groups at each age. Significantly different gene families were selected using the Dunn's post-hoc test and MaAsLin2 analysis. *P*-value was adjusted using the Benjamini–Hochberg method to calculate the *q* value (false discovery rate adjusted *p*-value). N: Non-AD, M: Mild AD, MS: Moderate to Severe AD.

Table S11. Comparison of the shifted KEGG Orthology (KO) according to age among phenotypic groups. Significantly changed categories according to age were selected using MaAsLin2 ($q < 0.05$) and the random forest model (the lowest cross-validation error). KO features of the gut microbiome at 6 months were used as a reference for MaAsLin2 analysis. Kruskal–Wallis and Dunn's post-hoc tests were performed to calculate the significant differences between groups. *P*-value was adjusted using the Benjamini–Hochberg method. N: Non-AD, M: Mild AD, MS: Moderate to Severe AD.

Table S12. Primer list for gene expression of mice colon.

Fig. S1

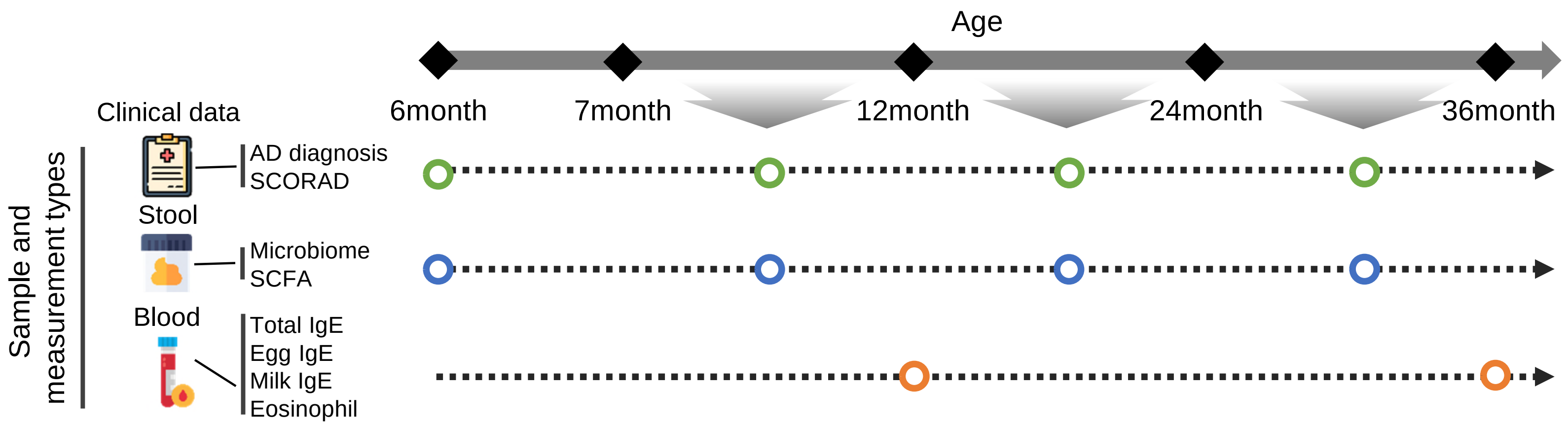

Age
6month
7month
12month
24month
36month
Clinical data
AD diagnosis
SCORAD
Stool
Microbiome
SCFA
Blood
Total IgE
Egg IgE
Milk IgE
Eosinophil
Sample and measurement types

Fig. S2

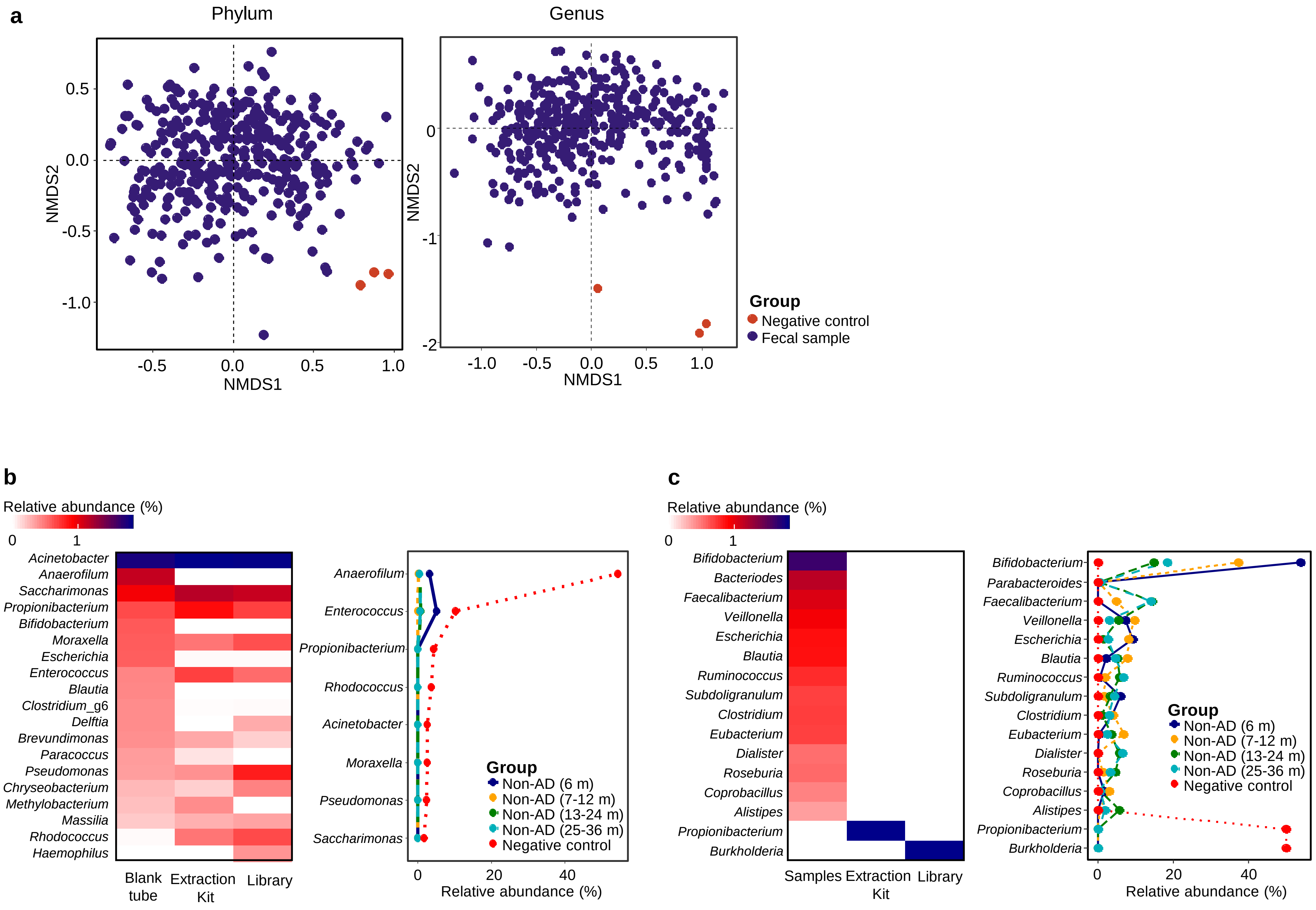
a
Phylum
Genus
NMDS1
NMDS2
0.5
0.0
-0.5
-1.0
1.0
0
-1
-2
Group
Negative control
Fecal sample
b
Relative abundance (%)
0
1
Acinetobacter
Anaerofilum
Saccharimonas
Propionibacterium
Bifidobacterium
Moraxella
Escherichia
Enterococcus
Blautia
Clostridium_g6
Delftia
Brevundimonas
Paracoccus
Pseudomonas
Chryseobacterium
Methylobacterium
Massilia
Rhodococcus
Haemophilus
Blank tube
Extraction Kit
Library
Anaerofilum
Enterococcus
Propionibacterium
Rhodococcus
Acinetobacter
Moraxella
Pseudomonas
Saccharimonas
Group
Non-AD (6 m)
Non-AD (7-12 m)
Non-AD (13-24 m)
Non-AD (25-36 m)
Negative control
0
20
40
Relative abundance (%)
c
Relative abundance (%)
0
1
Bifidobacterium
Bacteriodes
Faecalibacterium
Veillonella
Escherichia
Blautia
Ruminococcus
Subdoligranulum
Clostridium
Eubacterium
Dialister
Roseburia
Coprobacillus
Alistipes
Propionibacterium
Burkholderia
Samples
Extraction Kit
Library
Bifidobacterium
Parabacteroides
Faecalibacterium
Veillonella
Escherichia
Blautia
Ruminococcus
Subdoligranulum
Clostridium
Eubacterium
Dialister
Roseburia
Coprobacillus
Alistipes
Propionibacterium
Burkholderia
Group
Non-AD (6 m)
Non-AD (7-12 m)
Non-AD (13-24 m)
Non-AD (25-36 m)
Negative control
0
20
40
Relative abundance (%)

Fig. S3

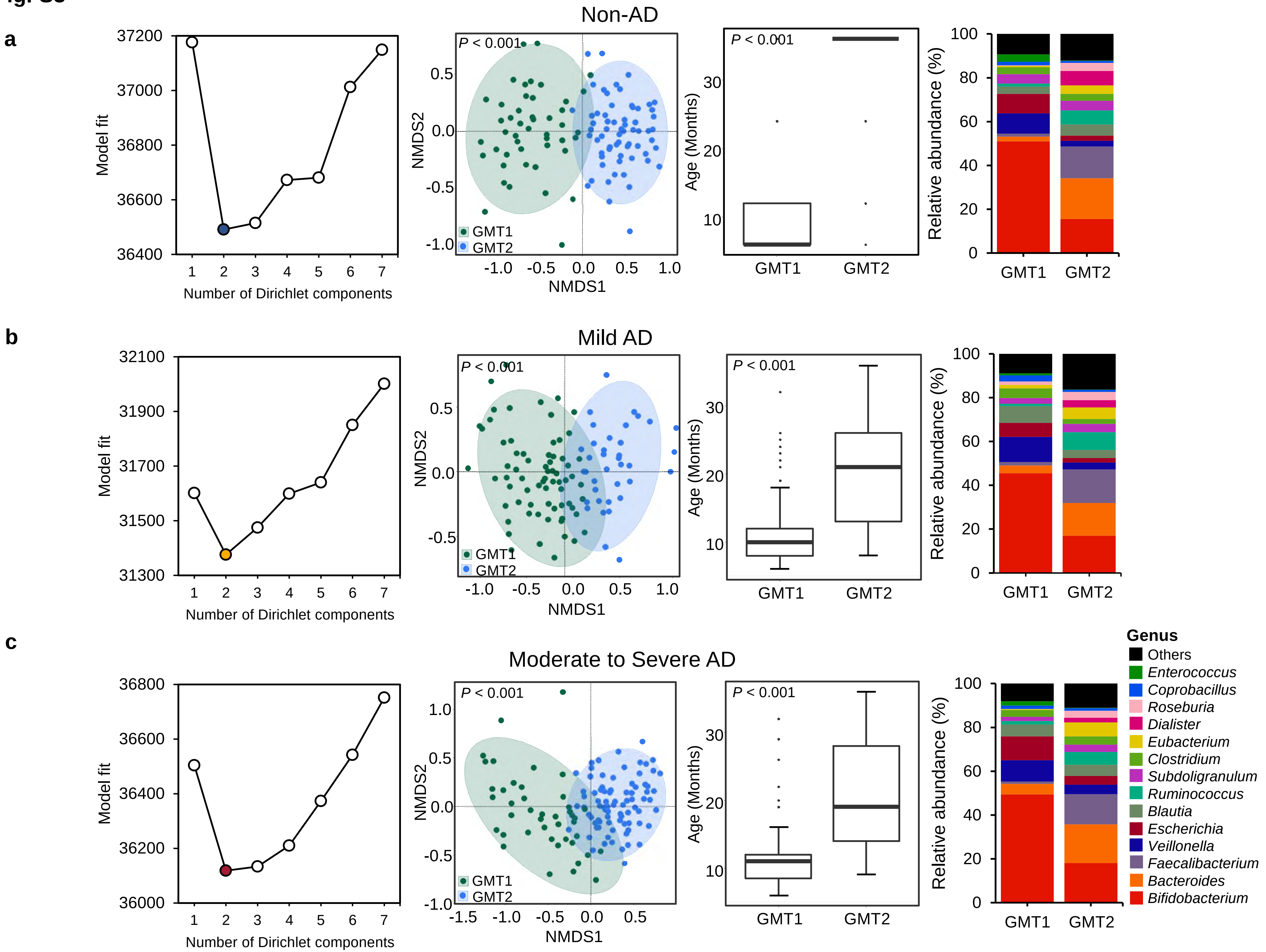

a
Non-AD
Model fit
Number of Dirichlet components
P < 0.001
GMT1
GMT2
NMDS1
NMDS2
Age (Months)
Relative abundance (%)
b
Mild AD
c
Moderate to Severe AD
Genus
Others
Enterococcus
Coprobacillus
Roseburia
Dialister
Eubacterium
Clostridium
Subdoligranulum
Ruminococcus
Blautia
Escherichia
Veillonella
Faecalibacterium
Bacteroides
Bifidobacterium

Fig. S4

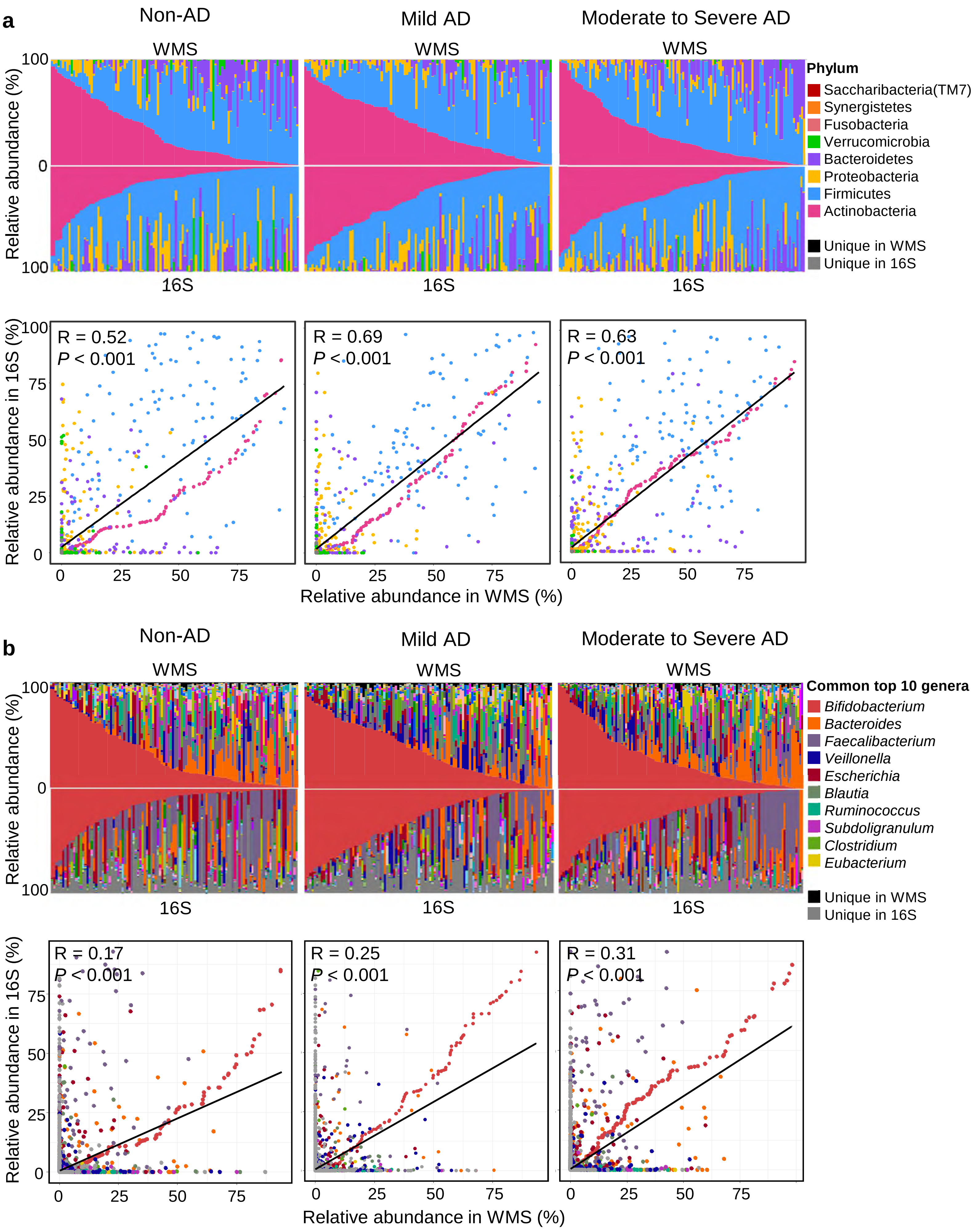
a
Non-AD
Mild AD
Moderate to Severe AD
WMS
16S
Relative abundance (%)
100
0
100
Phylum
Saccharibacteria(TM7)
Synergistetes
Fusobacteria
Verrucomicrobia
Bacteroidetes
Proteobacteria
Firmicutes
Actinobacteria
Unique in WMS
Unique in 16S
R = 0.52
P < 0.001
R = 0.69
P < 0.001
R = 0.63
P < 0.001
Relative abundance in 16S (%)
Relative abundance in WMS (%)
0
25
50
75
100
b
Non-AD
Mild AD
Moderate to Severe AD
WMS
16S
Relative abundance (%)
Common top 10 genera
Bifidobacterium
Bacteroides
Faecalibacterium
Veillonella
Escherichia
Blautia
Ruminococcus
Subdoligranulum
Clostridium
Eubacterium
Unique in WMS
Unique in 16S
R = 0.17
P < 0.001
R = 0.25
P < 0.001
R = 0.31
P < 0.001
Relative abundance in 16S (%)
Relative abundance in WMS (%)

Fig. S5

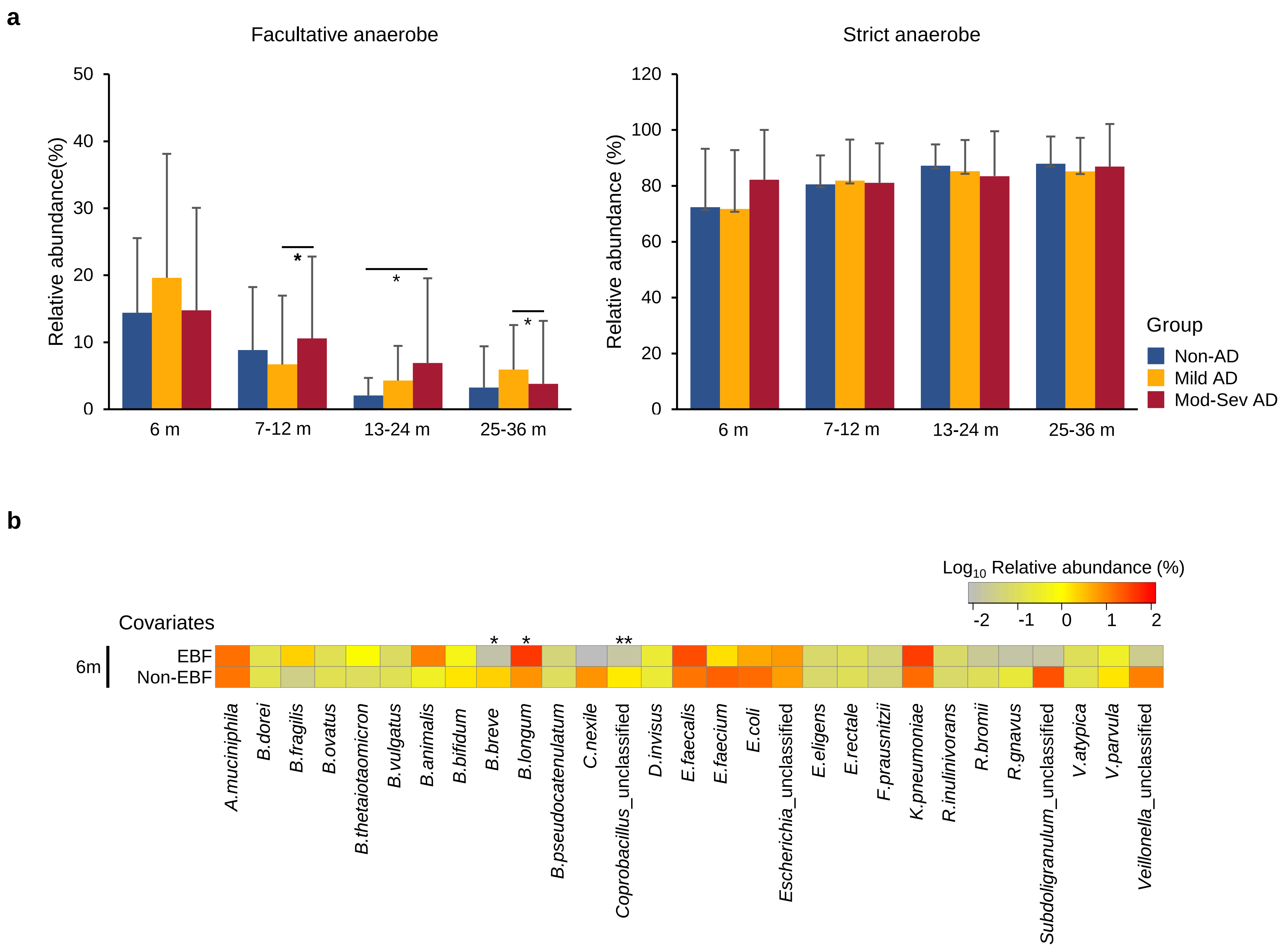
a
Facultative anaerobe
Strict anaerobe
Relative abundance(%)
Relative abundance (%)
6 m
7-12 m
13-24 m
25-36 m
Group
Non-AD
Mild AD
Mod-Sev AD
b
Log10 Relative abundance (%)
Covariates
6m
EBF
Non-EBF
A.muciniphila
B.dorei
B.fragilis
B.ovatus
B.thetaiotaomicron
B.vulgatus
B.animalis
B.bifidum
B.breve
B.longum
B.pseudocatenulatum
C.nexile
Coprobacillus_unclassified
D.invisus
E.faecalis
E.faecium
E.coli
Escherichia_unclassified
E.eligens
E.rectale
F.prausnitzii
K.pneumoniae
R.inulinivorans
R.bromii
R.gnavus
Subdoligranulum_unclassified
V.atypica
V.parvula
Veillonella_unclassified

Fig. S6

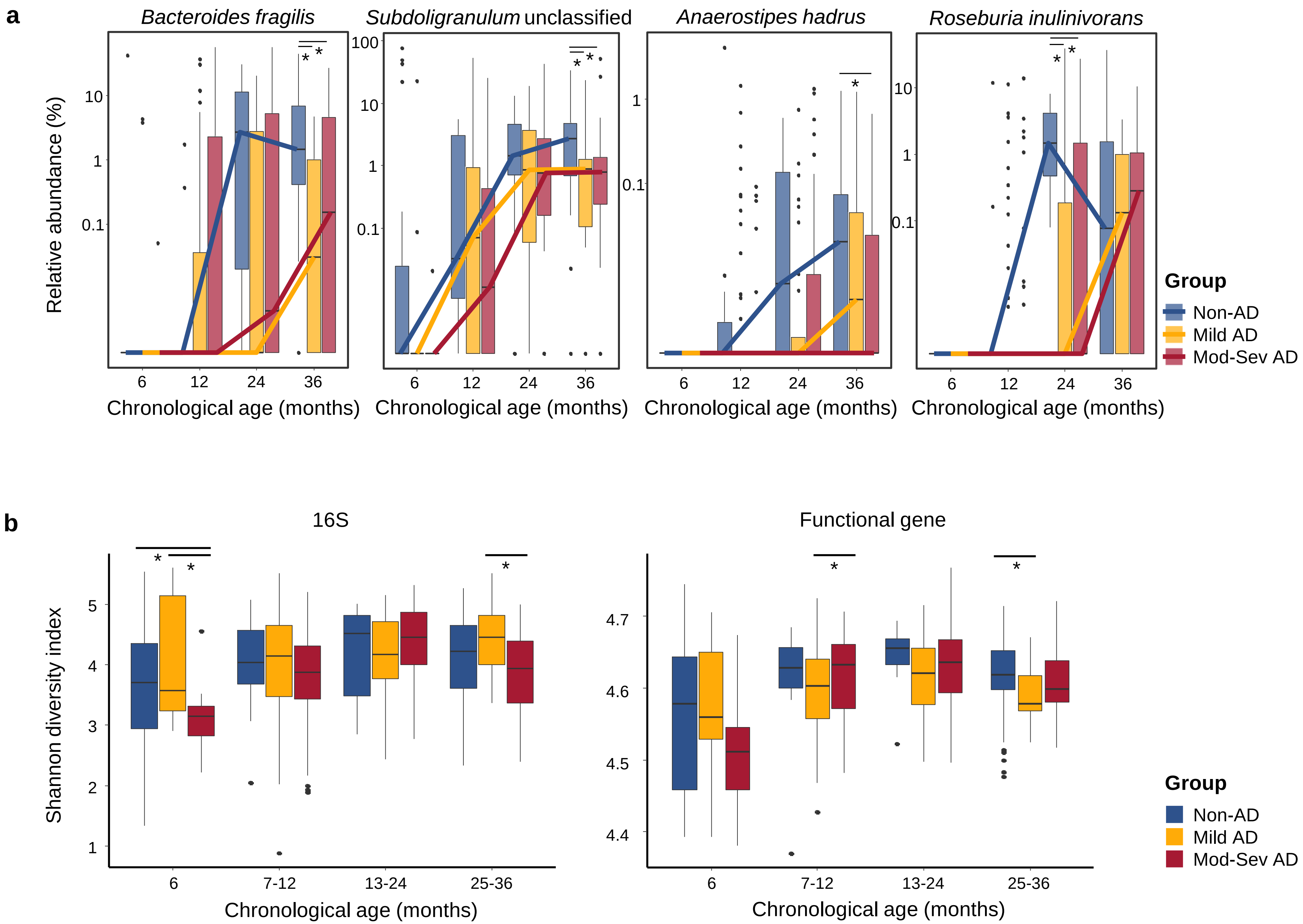

a
Bacteroides fragilis
Subdoligranulum unclassified
Anaerostipes hadrus
Roseburia inulinivorans
Relative abundance (%)
Chronological age (months)
6
12
24
36
Group
Non-AD
Mild AD
Mod-Sev AD
b
16S
Functional gene
Shannon diversity index
6
7-12
13-24
25-36
Chronological age (months)
Group
Non-AD
Mild AD
Mod-Sev AD

Fig. S7

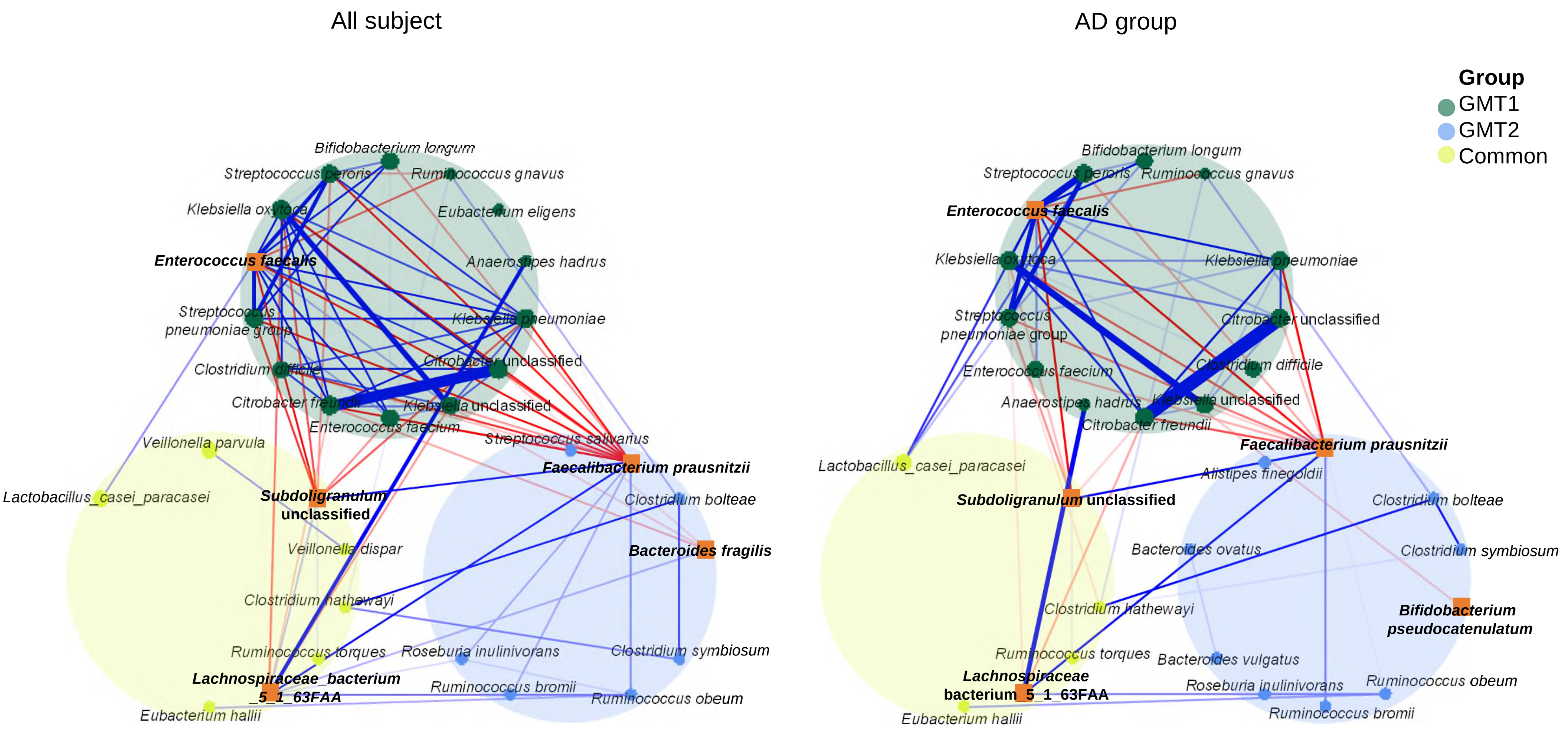
All subject
AD group
Group
GMT1
GMT2
Common
Bifidobacterium longum
Streptococcus peroris
Ruminococcus gnavus
Eubacterium eligens
Klebsiella oxytoca
Enterococcus faecalis
Anaerostipes hadrus
Streptococcus pneumoniae group
Klebsiella pneumoniae
Clostridium difficile
Citrobacter unclassified
Citrobacter freundii
Klebsiella unclassified
Enterococcus faecium
Streptococcus salivarius
Veillonella parvula
Faecalibacterium prausnitzii
Lactobacillus_casei_paracasei
Subdoligranulum unclassified
Clostridium bolteae
Veillonella dispar
Bacteroides fragilis
Clostridium hathewayi
Ruminococcus torques
Roseburia inulinivorans
Clostridium symbiosum
Lachnospiraceae_bacterium _5_1_63FAA
Ruminococcus bromii
Ruminococcus obeum
Eubacterium hallii
Alistipes finegoldii
Bacteroides ovatus
Bifidobacterium pseudocatenulatum
Bacteroides vulgatus
Lachnospiraceae bacterium_5_1_63FAA

Fig. S8

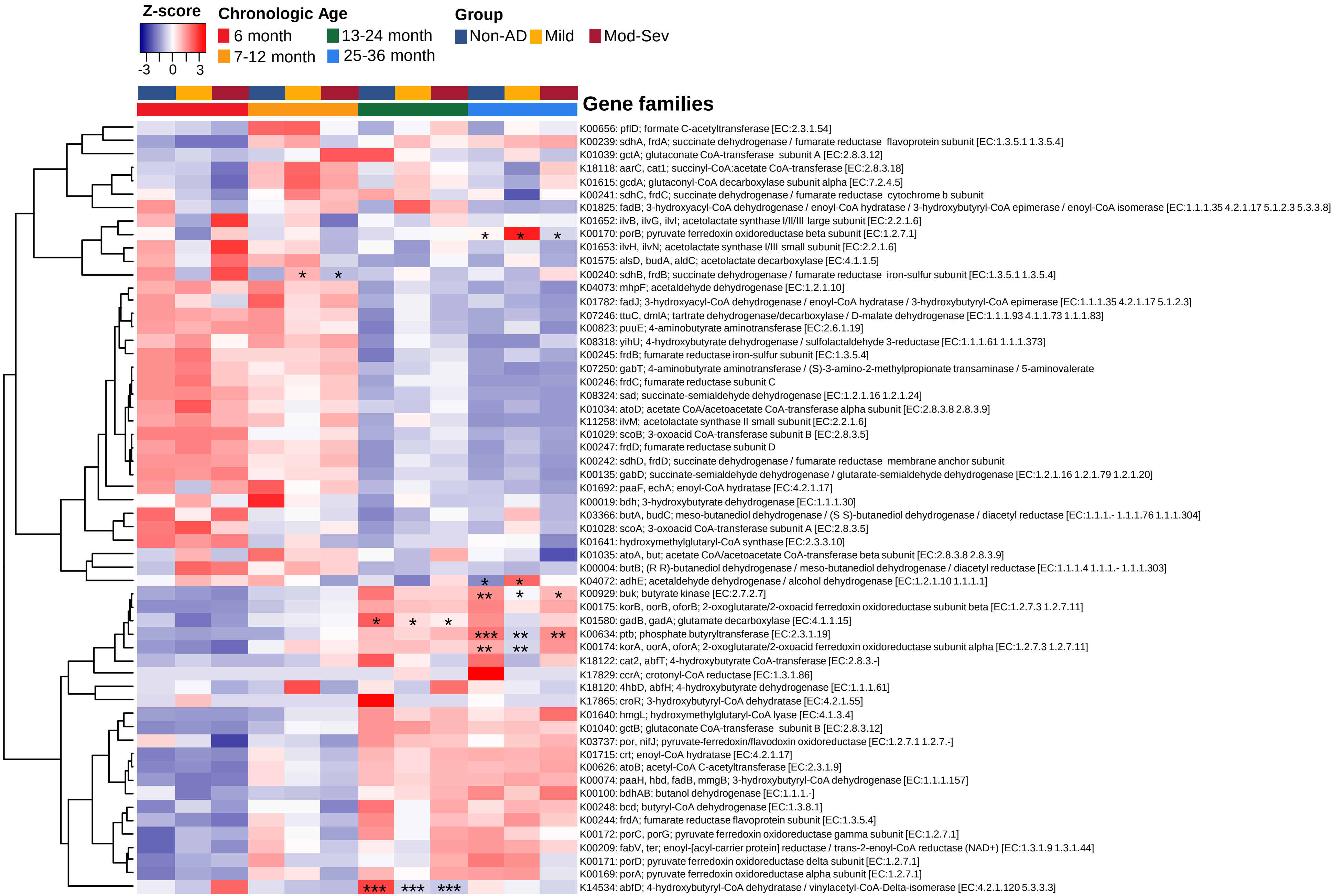

Z-score
-3 0 3
Chronologic Age
6 month
7-12 month
13-24 month
25-36 month
Group
Non-AD
Mild
Mod-Sev
Gene families
K00656: pflD; formate C-acetyltransferase [EC:2.3.1.54]
K00239: sdhA, frdA; succinate dehydrogenase / fumarate reductase flavoprotein subunit [EC:1.3.5.1 1.3.5.4]
K01039: gctA; glutaconate CoA-transferase subunit A [EC:2.8.3.12]
K18118: aarC, cat1; succinyl-CoA:acetate CoA-transferase [EC:2.8.3.18]
K01615: gcdA; glutaconyl-CoA decarboxylase subunit alpha [EC:7.2.4.5]
K00241: sdhC, frdC; succinate dehydrogenase / fumarate reductase cytochrome b subunit
K01825: fadB; 3-hydroxyacyl-CoA dehydrogenase / enoyl-CoA hydratase / 3-hydroxybutyryl-CoA epimerase / enoyl-CoA isomerase [EC:1.1.1.35 4.2.1.17 5.1.2.3 5.3.3.8]
K01652: ilvB, ilvG, ilvI; acetolactate synthase I/II/III large subunit [EC:2.2.1.6]
K00170: porB; pyruvate ferredoxin oxidoreductase beta subunit [EC:1.2.7.1]
K01653: ilvH, ilvN; acetolactate synthase I/III small subunit [EC:2.2.1.6]
K01575: alsD, budA, aldC; acetolactate decarboxylase [EC:4.1.1.5]
K00240: sdhB, frdB; succinate dehydrogenase / fumarate reductase iron-sulfur subunit [EC:1.3.5.1 1.3.5.4]
K04073: mhpF; acetaldehyde dehydrogenase [EC:1.2.1.10]
K01782: fadJ; 3-hydroxyacyl-CoA dehydrogenase / enoyl-CoA hydratase / 3-hydroxybutyryl-CoA epimerase [EC:1.1.1.35 4.2.1.17 5.1.2.3]
K07246: ttuC, dmlA; tartrate dehydrogenase/decarboxylase / D-malate dehydrogenase [EC:1.1.1.93 4.1.1.73 1.1.1.83]
K00823: puuE; 4-aminobutyrate aminotransferase [EC:2.6.1.19]
K08318: yihU; 4-hydroxybutyrate dehydrogenase / sulfolactaldehyde 3-reductase [EC:1.1.1.61 1.1.1.373]
K00245: frdB; fumarate reductase iron-sulfur subunit [EC:1.3.5.4]
K07250: gabT; 4-aminobutyrate aminotransferase / (S)-3-amino-2-methylpropionate transaminase / 5-aminovalerate
K00246: frdC; fumarate reductase subunit C
K08324: sad; succinate-semialdehyde dehydrogenase [EC:1.2.1.16 1.2.1.24]
K01034: atoD; acetate CoA/acetoacetate CoA-transferase alpha subunit [EC:2.8.3.8 2.8.3.9]
K11258: ilvM; acetolactate synthase II small subunit [EC:2.2.1.6]
K01029: scoB; 3-oxoacid CoA-transferase subunit B [EC:2.8.3.5]
K00247: frdD; fumarate reductase subunit D
K00242: sdhD, frdD; succinate dehydrogenase / fumarate reductase membrane anchor subunit
K00135: gabD; succinate-semialdehyde dehydrogenase / glutarate-semialdehyde dehydrogenase [EC:1.2.1.16 1.2.1.79 1.2.1.20]
K01692: paaF, echA; enoyl-CoA hydratase [EC:4.2.1.17]
K00019: bdh; 3-hydroxybutyrate dehydrogenase [EC:1.1.1.30]
K03366: butA, budC; meso-butanediol dehydrogenase / (S S)-butanediol dehydrogenase / diacetyl reductase [EC:1.1.1.- 1.1.1.76 1.1.1.304]
K01028: scoA; 3-oxoacid CoA-transferase subunit A [EC:2.8.3.5]
K01641: hydroxymethylglutaryl-CoA synthase [EC:2.3.3.10]
K01035: atoA, but; acetate CoA/acetoacetate CoA-transferase beta subunit [EC:2.8.3.8 2.8.3.9]
K00004: butB; (R R)-butanediol dehydrogenase / meso-butanediol dehydrogenase / diacetyl reductase [EC:1.1.1.4 1.1.1.- 1.1.1.303]
K04072: adhE; acetaldehyde dehydrogenase / alcohol dehydrogenase [EC:1.2.1.10 1.1.1.1]
K00929: buk; butyrate kinase [EC:2.7.2.7]
K00175: korB, oorB, oforB; 2-oxoglutarate/2-oxoacid ferredoxin oxidoreductase subunit beta [EC:1.2.7.3 1.2.7.11]
K01580: gadB, gadA; glutamate decarboxylase [EC:4.1.1.15]
K00634: ptb; phosphate butyryltransferase [EC:2.3.1.19]
K00174: korA, oorA, oforA; 2-oxoglutarate/2-oxoacid ferredoxin oxidoreductase subunit alpha [EC:1.2.7.3 1.2.7.11]
K18122: cat2, abfT; 4-hydroxybutyrate CoA-transferase [EC:2.8.3.-]
K17829: ccrA; crotonyl-CoA reductase [EC:1.3.1.86]
K18120: 4hbD, abfH; 4-hydroxybutyrate dehydrogenase [EC:1.1.1.61]
K17865: croR; 3-hydroxybutyryl-CoA dehydratase [EC:4.2.1.55]
K01640: hmgL; hydroxymethylglutaryl-CoA lyase [EC:4.1.3.4]
K01040: gctB; glutaconate CoA-transferase subunit B [EC:2.8.3.12]
K03737: por, nifJ; pyruvate-ferredoxin/flavodoxin oxidoreductase [EC:1.2.7.1 1.2.7.-]
K01715: crt; enoyl-CoA hydratase [EC:4.2.1.17]
K00626: atoB; acetyl-CoA C-acetyltransferase [EC:2.3.1.9]
K00074: paaH, hbd, fadB, mmgB; 3-hydroxybutyryl-CoA dehydrogenase [EC:1.1.1.157]
K00100: bdhAB; butanol dehydrogenase [EC:1.1.1.-]
K00248: bcd; butyryl-CoA dehydrogenase [EC:1.3.8.1]
K00244: frdA; fumarate reductase flavoprotein subunit [EC:1.3.5.4]
K00172: porC, porG; pyruvate ferredoxin oxidoreductase gamma subunit [EC:1.2.7.1]
K00209: fabV, ter; enoyl-[acyl-carrier protein] reductase / trans-2-enoyl-CoA reductase (NAD+) [EC:1.3.1.9 1.3.1.44]
K00171: porD; pyruvate ferredoxin oxidoreductase delta subunit [EC:1.2.7.1]
K00169: porA; pyruvate ferredoxin oxidoreductase alpha subunit [EC:1.2.7.1]
K14534: abfD; 4-hydroxybutyryl-CoA dehydratase / vinylacetyl-CoA-Delta-isomerase [EC:4.2.1.120 5.3.3.3]

Fig. S9

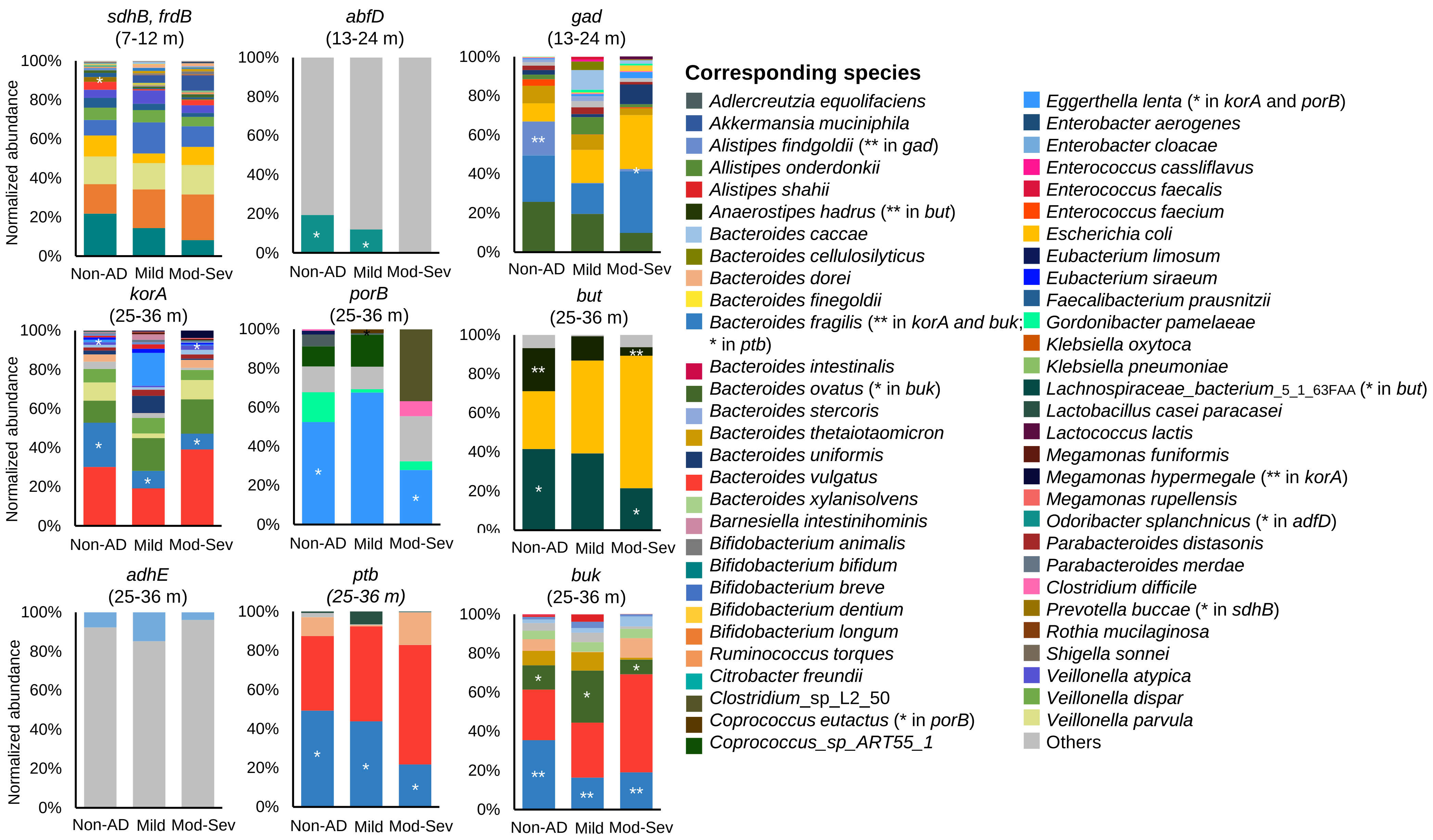

sdhB, frdB (7-12 m)
abfD (13-24 m)
gad (13-24 m)
korA (25-36 m)
porB (25-36 m)
but (25-36 m)
adhE (25-36 m)
ptb (25-36 m)
buk (25-36 m)
Normalized abundance
100%
80%
60%
40%
20%
0%
Non-AD
Mild
Mod-Sev
Corresponding species
Adlercreutzia equolifaciens
Akkermansia muciniphila
Alistipes findgoldii (** in gad)
Allistipes onderdonkii
Alistipes shahii
Anaerostipes hadrus (** in but)
Bacteroides caccae
Bacteroides cellulosilyticus
Bacteroides dorei
Bacteroides finegoldii
Bacteroides fragilis (** in korA and buk; * in ptb)
Bacteroides intestinalis
Bacteroides ovatus (* in buk)
Bacteroides stercoris
Bacteroides thetaiotaomicron
Bacteroides uniformis
Bacteroides vulgatus
Bacteroides xylanisolvens
Barnesiella intestinihominis
Bifidobacterium animalis
Bifidobacterium bifidum
Bifidobacterium breve
Bifidobacterium dentium
Bifidobacterium longum
Ruminococcus torques
Citrobacter freundii
Clostridium_sp_L2_50
Coprococcus eutactus (* in porB)
Coprococcus_sp_ART55_1
Eggerthella lenta (* in korA and porB)
Enterobacter aerogenes
Enterobacter cloacae
Enterococcus cassliflavus
Enterococcus faecalis
Enterococcus faecium
Escherichia coli
Eubacterium limosum
Eubacterium siraeum
Faecalibacterium prausnitzii
Gordonibacter pamelaeae
Klebsiella oxytoca
Klebsiella pneumoniae
Lachnospiraceae_bacterium_5_1_63FAA (* in but)
Lactobacillus casei paracasei
Lactococcus lactis
Megamonas funiformis
Megamonas hypermegale (** in korA)
Megamonas rupellensis
Odoribacter splanchnicus (* in adfD)
Parabacteroides distasonis
Parabacteroides merdae
Clostridium difficile
Prevotella buccae (* in sdhB)
Rothia mucilaginosa
Shigella sonnei
Veillonella atypica
Veillonella dispar
Veillonella parvula
Others

Fig. S10

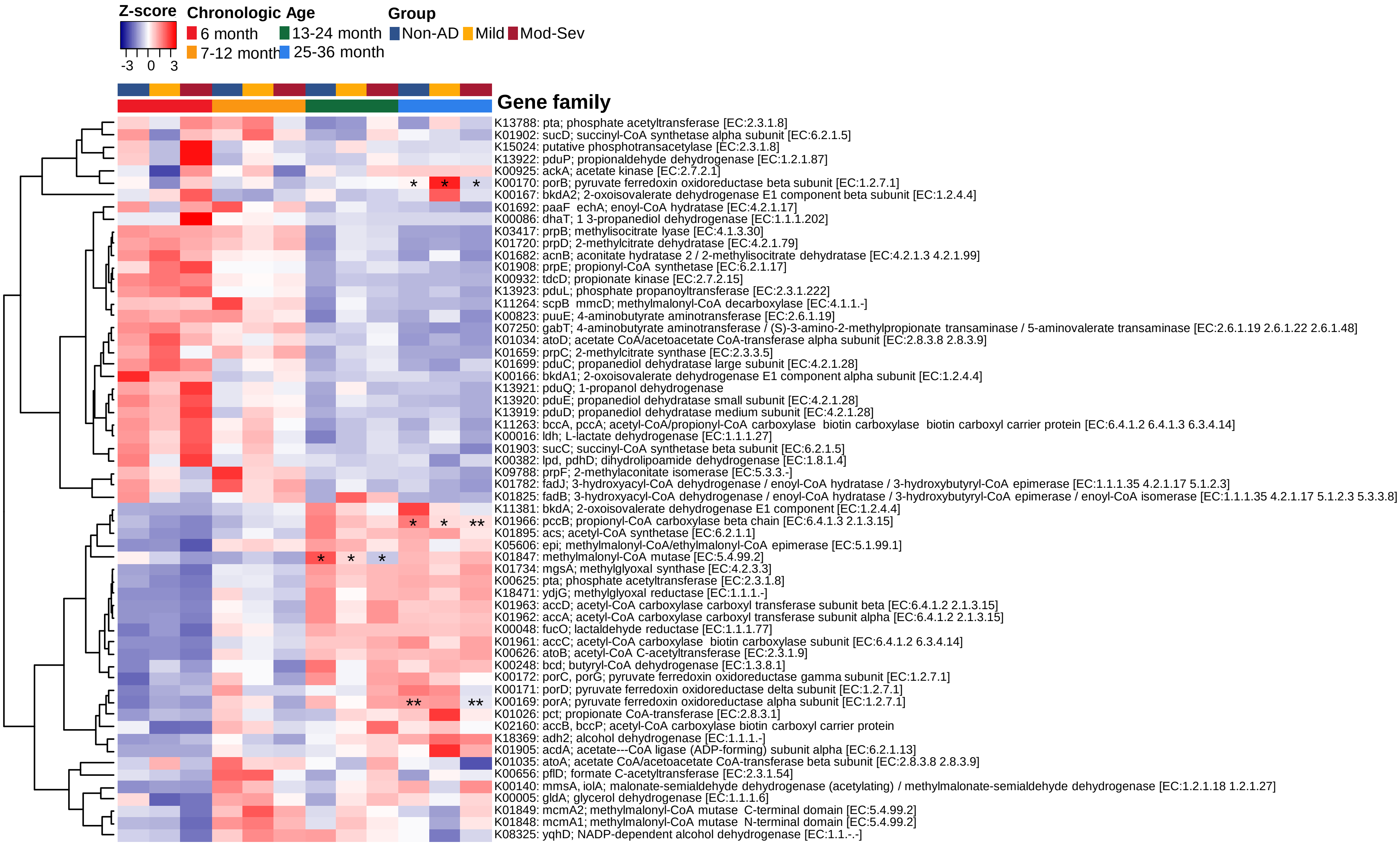

Z-score
-3
0
3
Chronologic Age
6 month
7-12 month
13-24 month
25-36 month
Group
Non-AD
Mild
Mod-Sev
Gene family
K13788: pta; phosphate acetyltransferase [EC:2.3.1.8]
K01902: sucD; succinyl-CoA synthetase alpha subunit [EC:6.2.1.5]
K15024: putative phosphotransacetylase [EC:2.3.1.8]
K13922: pduP; propionaldehyde dehydrogenase [EC:1.2.1.87]
K00925: ackA; acetate kinase [EC:2.7.2.1]
K00170: porB; pyruvate ferredoxin oxidoreductase beta subunit [EC:1.2.7.1]
K00167: bkdA2; 2-oxoisovalerate dehydrogenase E1 component beta subunit [EC:1.2.4.4]
K01692: paaF echA; enoyl-CoA hydratase [EC:4.2.1.17]
K00086: dhaT; 1 3-propanediol dehydrogenase [EC:1.1.1.202]
K03417: prpB; methylisocitrate lyase [EC:4.1.3.30]
K01720: prpD; 2-methylcitrate dehydratase [EC:4.2.1.79]
K01682: acnB; aconitate hydratase 2 / 2-methylisocitrate dehydratase [EC:4.2.1.3 4.2.1.99]
K01908: prpE; propionyl-CoA synthetase [EC:6.2.1.17]
K00932: tdcD; propionate kinase [EC:2.7.2.15]
K13923: pduL; phosphate propanoyltransferase [EC:2.3.1.222]
K11264: scpB mmcD; methylmalonyl-CoA decarboxylase [EC:4.1.1.-]
K00823: puuE; 4-aminobutyrate aminotransferase [EC:2.6.1.19]
K07250: gabT; 4-aminobutyrate aminotransferase / (S)-3-amino-2-methylpropionate transaminase / 5-aminovalerate transaminase [EC:2.6.1.19 2.6.1.22 2.6.1.48]
K01034: atoD; acetate CoA/acetoacetate CoA-transferase alpha subunit [EC:2.8.3.8 2.8.3.9]
K01659: prpC; 2-methylcitrate synthase [EC:2.3.3.5]
K01699: pduC; propanediol dehydratase large subunit [EC:4.2.1.28]
K00166: bkdA1; 2-oxoisovalerate dehydrogenase E1 component alpha subunit [EC:1.2.4.4]
K13921: pduQ; 1-propanol dehydrogenase
K13920: pduE; propanediol dehydratase small subunit [EC:4.2.1.28]
K13919: pduD; propanediol dehydratase medium subunit [EC:4.2.1.28]
K11263: bccA, pccA; acetyl-CoA/propionyl-CoA carboxylase biotin carboxylase biotin carboxyl carrier protein [EC:6.4.1.2 6.4.1.3 6.3.4.14]
K00016: ldh; L-lactate dehydrogenase [EC:1.1.1.27]
K01903: sucC; succinyl-CoA synthetase beta subunit [EC:6.2.1.5]
K00382: lpd, pdhD; dihydrolipoamide dehydrogenase [EC:1.8.1.4]
K09788: prpF; 2-methylaconitate isomerase [EC:5.3.3.-]
K01782: fadJ; 3-hydroxyacyl-CoA dehydrogenase / enoyl-CoA hydratase / 3-hydroxybutyryl-CoA epimerase [EC:1.1.1.35 4.2.1.17 5.1.2.3]
K01825: fadB; 3-hydroxyacyl-CoA dehydrogenase / enoyl-CoA hydratase / 3-hydroxybutyryl-CoA epimerase / enoyl-CoA isomerase [EC:1.1.1.35 4.2.1.17 5.1.2.3 5.3.3.8]
K11381: bkdA; 2-oxoisovalerate dehydrogenase E1 component [EC:1.2.4.4]
K01966: pccB; propionyl-CoA carboxylase beta chain [EC:6.4.1.3 2.1.3.15]
K01895: acs; acetyl-CoA synthetase [EC:6.2.1.1]
K05606: epi; methylmalonyl-CoA/ethylmalonyl-CoA epimerase [EC:5.1.99.1]
K01847: methylmalonyl-CoA mutase [EC:5.4.99.2]
K01734: mgsA; methylglyoxal synthase [EC:4.2.3.3]
K00625: pta; phosphate acetyltransferase [EC:2.3.1.8]
K18471: ydjG; methylglyoxal reductase [EC:1.1.1.-]
K01963: accD; acetyl-CoA carboxylase carboxyl transferase subunit beta [EC:6.4.1.2 2.1.3.15]
K01962: accA; acetyl-CoA carboxylase carboxyl transferase subunit alpha [EC:6.4.1.2 2.1.3.15]
K00048: fucO; lactaldehyde reductase [EC:1.1.1.77]
K01961: accC; acetyl-CoA carboxylase biotin carboxylase subunit [EC:6.4.1.2 6.3.4.14]
K00626: atoB; acetyl-CoA C-acetyltransferase [EC:2.3.1.9]
K00248: bcd; butyryl-CoA dehydrogenase [EC:1.3.8.1]
K00172: porC, porG; pyruvate ferredoxin oxidoreductase gamma subunit [EC:1.2.7.1]
K00171: porD; pyruvate ferredoxin oxidoreductase delta subunit [EC:1.2.7.1]
K00169: porA; pyruvate ferredoxin oxidoreductase alpha subunit [EC:1.2.7.1]
K01026: pct; propionate CoA-transferase [EC:2.8.3.1]
K02160: accB, bccP; acetyl-CoA carboxylase biotin carboxyl carrier protein
K18369: adh2; alcohol dehydrogenase [EC:1.1.1.-]
K01905: acdA; acetate---CoA ligase (ADP-forming) subunit alpha [EC:6.2.1.13]
K01035: atoA; acetate CoA/acetoacetate CoA-transferase beta subunit [EC:2.8.3.8 2.8.3.9]
K00656: pflD; formate C-acetyltransferase [EC:2.3.1.54]
K00140: mmsA, iolA; malonate-semialdehyde dehydrogenase (acetylating) / methylmalonate-semialdehyde dehydrogenase [EC:1.2.1.18 1.2.1.27]
K00005: gldA; glycerol dehydrogenase [EC:1.1.1.6]
K01849: mcmA2; methylmalonyl-CoA mutase C-terminal domain [EC:5.4.99.2]
K01848: mcmA1; methylmalonyl-CoA mutase N-terminal domain [EC:5.4.99.2]
K08325: yqhD; NADP-dependent alcohol dehydrogenase [EC:1.1.-.-]

Table S1. Simulated effect sizes (ω2) for PERMANOVA power of 80% and 90% with varied sample sizes. The micropower R-package was used to calculate the statistical power of sample sizes based on the weighted UniFrac distance of within-group distance variance with a significance level of 0.05.

Sample numbers in each group	Simulated omega2 (ω2)	
	power 80%	power 90%
20	0.3489	0.5773
40	0.0624	0.2551
60	0.0013	0.0409
80	< 0.0001	0.0010
100	< 0.0001	< 0.0001
120	< 0.0001	< 0.0001

Table S2. Clinical characteristics of the study subjects.

Variables	Non-AD	AD		*P* value
		Mild AD	Moderate to Severe AD	
Number of subjects at each age (6/ 7-12/ 13-24/ 25-36 months)	31/ 15/ 12/ 54	13/ 52/ 32/ 13	9/ 37/ 49/ 29	
Number of samples	112	110	124	0.474
Male (%)	52.7	54.5	66.9	0.052
SCORAD index	NA	10.6 ± 2.9	40.4 ± 14.2	<0.001
Delivery mode, vaginal delivery/ cesarean delivery/ unknown (%)	62.5/ 35.7/ 1.8	42.7/ 24.6/ 32.7	47.6/ 19.4/ 33.0	0.492
Parental history of allergic diseases, yes (%)	37.5	74.5	68.5	<0.001
Serum eosinophils (%)	3.1 ± 1.7	4.8 ± 2.9	9.7 ± 23.7	<0.001
Total IgE (IU/ml)	25.5 ± 31.3	115.8 ± 266.1	326.1 ± 791.4	<0.001
Egg IgE (IU/ml)	0.1 ± 0.4	7.3 ± 17.7	9.4 ± 17.9	<0.001
Milk IgE (IU/ml)	0.2 ± 0.7	0.9 ± 1.8	5.3 ± 16.8	<0.001
Feeding				0.1155
Exclusive breastfeeding (%)	33.9	48.2	50	
Non-exclusive breastfeeding (%)	19.6	14.5	14.5	
Unknown (%)	46.5	37.3	35.5	
Solid-food intake (starting month)	5.4 ± 0.68	5.7 ± 0.97	5.8 ± 0.95	0.0716

Table S3. Number of samples analyzed for each dataset at each age.

Data Type	Phenotype	6months	7-12 months	13-24 months	25-36 months
16S	Non-AD	31	15	12	54
	Mild AD	13	52	32	13
	Moderate-Severe AD	9	37	49	29
WMS	Non-AD	31	15	12	54
	Mild AD	13	49	32	13
	Moderate-Severe AD	9	37	49	29
RTP	Non-AD	19	15	12	54
	Mild AD	6	52	32	13
	Moderate-Severe AD	8	37	49	29
SCFA	Non-AD	31	15	12	54
	Mild AD	13	49	32	13
	Moderate-Severe AD	9	37	49	29

Table S4. Comparison of odds ratio (OR) for covariates between GMT1 and GMT2. CI: confidence interval.

Dataset	Variable	GMT1 (n=163)	GMT2 (n=180)	Odd ratio (95% CI), *P* value<br>GMT1 vs GMT2
WMS	Non-AD/ Mild AD/ Moderate to Severe AD	46/ 64/ 53	66/ 43/ 71	0.978 (0.757-1.263, *P* =0.866)
	Age	10.0 ± 7.64	24.0 ± 9.63	1.163 (0.081-1.163, *P* <2e-16)
	Vaginal delivery / C-section	85 (53.8) / 46 (29.1)	92 (48.9) / 43 (22.9)	0.864 (0.518-1.438, *P*= 0.573)
	Exclusive breastfeeding	81 (49.7)	70 (38.9)	1.549 (1.009-2.387, *P*= 0.051)
	Family history	100 (63.3)	107 (56.9)	0.949 (0.614-1.467, *P*= 0.815)
	Egg IgE (>0.35 IU/ml)	70 (44.3)	63 (33.5)	0.828 (0.524-1.305, *P* =0.416)
	Milk IgE (>0.35 IU/ml)	49 (31.0)	50 (26.6)	1.031 (0.635-1.674, *P* =0.902)
**Dataset**	**Variable**	**GMT1 (n=158)**	**GMT2 (n=188)**	**Odd ratio (95% CI), *P* value<br>GMT1 vs GMT2**
16S	Non-AD/ Mild AD/ Moderate to Severe AD	45/ 62/ 51	67/ 48/ 73	0.991 (0.767-1.281, *P* =0.946)
	Age	10.0 ± 7.74	24.0 ± 10.16	1.138 (1.107-1.175, *P* <2e-16)
	Vaginal delivery / C-section	81 (51.3) / 45 (28.5)	97 (51.6) / 45 (23.9)	0.835 (0.502-1.389, *P*= 0.486)
	Exclusive breastfeeding	81 (51.3)	72 (38.3)	1.479 (0.964-2.276, P=0.082)
	Family history	105 (66.5)	103 (54.8)	1.009 (0.542-1.920, *P*= 0.976)
	Egg IgE (>0.35 IU/ml)	68 (43.0)	68 (36.2)	0.814 (0.516-1.281, *P*= 0.374)
	Milk IgE (>0.35 IU/ml)	49 (31.0)	51 (27.1)	0.886 (0.546-1.436, *P* =0.622)

Table S5. Mean relative abundance of top 20 genera between GMT1 and GMT2 determined by DMM modeling. We used the Benjamini–Hochberg method adjusted *p*-value to calculate the *q* value (false discovery rate adjusted *p*-value).

Dataset	Genus	Prevalence (%)	Mean relative abundance (%)		*q* value (corrected *P*)
			GMT1 (n=163)	GMT2 (n=180)	
WMS	*Bifidobacterium*	99.1	47.73	16.83	<2.2e-16
	Faecalibacterium	63.8	1.34	14.58	<2.2e-16
	Bacteroides	62.1	3.53	17.63	<2.2e-16
	Escherichia	91.5	8.56	2.71	3.4.E-12
	Ruminococcus	46.1	1.25	6.64	<2.2e-16
	Eubacterium	66.5	1.33	5.13	<2.2e-16
	Veillonella	97.4	10.21	3.51	9.2.E-04
	Roseburia	59.8	0.52	3.71	<2.2e-16
	Subdoligranulum	71.7	2.54	4.15	<2.2e-16
	Coprobacillus	78.7	2.18	1.00	9.5.E-02
	Enterococcus	54.2	1.85	0.17	<2.2e-16
	Alistipes	28.9	0.05	2.04	9.0.E-20
	Dialister	21.3	0.05	3.89	2.9.E-14
	Blautia	81.0	6.59	4.11	4.2.E-04
	*Erysipelotrichaceae_* noname	77.8	0.89	0.30	3.3.E-04
	Klebsiella	45.5	1.13	0.07	<2.2e-16
	Clostridium	85.7	3.65	3.29	7.1.E-05
	Parabacteroides	27.4	0.10	0.83	6.2.E-12
	Lactobacillus	43.7	1.33	0.36	2.8.E-07
	*Lachnospiraceae_* noname	73.8	0.39	1.07	<2.2e-16

Dataset	Genus	Prevalence (%)	Mean relative abundance (%)		*q value* (corrected *P*)
			GMT1 (n=158)	GMT2 (n=188)	
	Faecalibacterium	66.8	1.62	36.62	<2.2e-16
	Bifidobacterium	98.0	31.44	16.39	4.E-09
	Escherichia	84.7	16.17	2.63	<2.2e-16
	Bacteroides	63.0	5.01	12.73	<2.2e-16
	*Clostridium_* g6	76.6	4.62	0.69	3.E-07

16S	*Veillonella*	74.0	5.79	1.34	1.E-11
	*Ruminococcus_* g2	40.8	0.91	3.22	<2.2e-16
	Klebsiella	52.9	2.25	0.04	<2.2e-16
	Enterococcus	37.9	2.37	0.05	<2.2e-16
	*Clostridium_* g21	69.1	5.69	1.67	9.E-01
	Citrobacter	64.2	1.16	0.04	<2.2e-16
	Streptococcus	68.2	1.85	0.30	8.E-05
	Enterobacter	71.1	1.35	0.36	<2.2e-16
	*Eubacterium_* g23	26.6	0.11	1.74	<2.2e-16
	Blautia	76.9	2.78	3.17	6.E-06
	Dialister	22.8	0.00	1.81	2.E-16
	Kosakonia	57.2	0.57	0.02	<2.2e-16
	*Eubacterium_* g1	46.0	0.76	0.07	2.E-08
	Anaerostipes	61.3	0.71	1.08	2.E-15
	Roseburia	53.5	0.46	0.83	<2.2e-16

Table S6. Comparison of indicator species for each age among phenotypic groups. Indicator species were selected after prefiltering by > 10% of prevalence and > 0.01% relative abundance in at least one group.

Indicator species	Indicator Age	R-squared	*P* value	Non-AD		Mild AD		Moderate to Severe AD	
				Mean relative abundance (%)	Prevalence (%)	Mean relative abundance (%)	Prevalence (%)	Mean relative abundance (%)	Prevalence (%)
Bifidobacterium longum	6 months	0.546	0.0002	39.22	100.0	40.49	100.0	62.11	100.0
Enterococcus faecalis	6 months	0.364	0.0066	1.24	74.2	0.93	76.9	2.14	66.7
Streptococcus mitis oralis pneumoniae	6 months	0.335	0.0034	0.14	64.5	0.06	61.5	0.07	66.7
Klebsiella pneumoniae	6 months	0.329	0.0118	1.20	71.0	1.56	61.5	0.60	44.4
Escherichia coli	6-12 months	0.369	0.0071	7.35	91.3	5.15	93.5	7.20	89.1
*Klebsiella_* unclassified	6-12 months	0.330	0.0120	0.10	54.3	0.09	45.2	0.06	28.3
Megasphaera micronuciformis	7-12 months	0.355	0.0048	1.41	26.7	0.34	38.8	0.23	35.1
Bifidobacterium bifidum	7-12 months	0.320	0.0213	18.36	53.3	11.77	65.3	8.24	48.6
Bifidobacterium breve	7-12 months	0.318	0.0180	6.72	73.3	13.06	89.8	8.53	75.7
Bacteroides ovatus	13-24 months	0.389	0.0018	5.36	41.7	2.70	31.3	1.27	30.6
*Oscillibacter_* unclassified	13-24 months	0.352	0.0065	1.49	50.0	0.25	21.9	0.15	22.4
Alistipes finegoldii	13-24 months	0.330	0.0033	3.08	66.7	0.03	6.3	0.12	6.1
Alistipes _unclassified	13-24 months	0.323	0.0020	1.98	33.3	0.07	3.1	0.09	8.2
Faecalibacterium prausnitzii	13-36 months	0.529	0.0001	14.15	90.9	11.24	84.4	13.37	87.2
Bacteroides fragilis	13-36 months	0.358	0.0075	6.26	89.4	2.00	53.3	4.65	57.7
Ruminococcus bromii	13-36 months	0.341	0.0150	4.95	63.6	4.26	42.2	4.53	48.7
Dialister invisus	13-36 months	0.303	0.0217	6.27	48.5	2.13	24.4	2.10	26.9

Table S7. Indicator species at each time point were evaluated after adjusting sex, feeding type, AD phenotype, and family history using MaAsLin 2 ($q < 0.25$). The time point of 6 months was used as a reference for the analysis. The analyzed species were selected after prefiltering for > 10% of prevalence and > 0.01% relative abundance in at least one group. *P*-value was adjusted by the Benjamini–Hochberg method to calculate the *q* value (false discovery rate adjusted *p*-value).

Species	Time point	Coefficient	Standard error	N	N not 0	*P* value	*q* value
Bifidobacterium longum	7-12 months	-0.2079	0.0246	343	331	8.72E-16	6.49E-13
Bifidobacterium longum	13-24 months	-0.2525	0.0259	343	331	5.93E-20	8.82E-17
Bifidobacterium longum	25-36 months	-0.2734	0.0239	343	331	8.76E-26	2.61E-22
Enterococcus faecalis	7-12 months	-0.0102	0.0033	343	80	0.0022524	0.1117579
Enterococcus faecalis	13-24 months	-0.0122	0.0035	343	80	0.0005296	0.0463668
Enterococcus faecalis	25-36 months	-0.0135	0.0032	343	80	3.63E-05	0.0060058
Streptococcus mitis oralis pneumoniae	7-12 months	-0.0009	0.0002	343	86	3.38E-06	0.0008394
Streptococcus mitis oralis pneumoniae	13-24 months	-0.0010	0.0002	343	86	1.40E-06	0.0003792
Streptococcus mitis oralis pneumoniae	25-36 months	-0.0011	0.0002	343	86	1.07E-08	4.56E-06
Klebsiella pneumoniae	25-36 months	-0.0119	0.0037	343	107	0.001543	0.0866725
Escherichia coli	7-12 months	-0.0408	0.0119	343	302	0.0006784	0.0557524
Escherichia coli	13-24 months	-0.0618	0.0125	343	302	1.25E-06	0.0003715
Escherichia coli	25-36 months	-0.0644	0.0116	343	302	5.32E-08	1.76E-05
*Klebsiella_* unclassified	13-24 months	-0.0009	0.0003	343	95	0.0078434	0.2398792
*Klebsiella_* unclassified	25-36 months	-0.0009	0.0003	343	95	0.0054211	0.1968131
Megasphaera micronuciformis	7-12 months	0.0046	0.0015	343	101	0.0019076	0.0979105
Bifidobacterium breve	7-12 months	0.0443	0.0148	343	256	0.0030533	0.1336729
Bacteroides ovatus	13-24 months	0.0242	0.0069	343	100	0.0005059	0.0456365
*Oscillibacter_* unclassified	13-24 months	0.0053	0.0015	343	84	0.0003709	0.035614
Alistipes _unclassified	13-24 months	0.0049	0.0018	343	23	0.0060509	0.213485
Faecalibacterium prausnitzii	13-24 months	0.0943	0.0166	343	219	3.06E-08	1.14E-05
Faecalibacterium prausnitzii	25-36 months	0.1374	0.0153	343	219	2.55E-17	2.53E-14
Bacteroides fragilis	13-24 months	0.0422	0.0139	343	160	0.0026301	0.1204564
Ruminococcus bromii	13-24 months	0.0417	0.0122	343	129	0.00075	0.0557524
Ruminococcus bromii	25-36 months	0.0414	0.0113	343	129	0.0002982	0.0317012
Dialister invisus	25-36 months	0.0443	0.0111	343	68	7.86E-05	0.0097501

Table S8. Correlation between covariates and microbiota variation. The correlations were determined using the EnvFit model based on Bray–Curtis dissimilarity in each dataset.

Dataset	Variable	6 months		7-12 months		13-24 months		25-36 months	
		R-squared	*P* value	R-squared	*P* value	R-squared	*P* value	R-squared	*P* value
WMS	Sex	0.029	0.574	0.013	0.686	0.007	0.865	0.028	0.557
	Mode of delivery	0.103	0.125	0.002	0.925	0.022	0.664	0.025	0.586
	Exclusive breastfeeding	0.306	<0.001	0.096	0.053	0.163	0.030	0.051	0.330
	Family history	0.038	0.447	0.035	0.338	0.019	0.661	0.010	0.815
	Non-AD/AD	0.049	0.117	0.036	0.117	0.031	0.286	0.042	0.167
	Non-AD/ Mild AD/ Moderate to Severe AD	0.091	0.095	0.097	0.017	0.047	0.454	0.080	0.129
	Egg IgE (IU/ml)	0.613	0.414	0.656	0.424	0.805	0.272	0.568	0.395
	Milk IgE (IU/ml)	0.483	0.861	0.621	0.632	0.624	0.668	0.793	0.061
Dataset	Variable	6 months		7-12 months		13-24 months		25-36 months	
		R-squared	*P* value	R-squared	*P* value	R-squared	*P* value	R-squared	*P* value
16S	Sex	0.021	0.630	0.003	0.932	0.003	0.932	0.029	0.539
	Mode of delivery	0.031	0.510	0.027	0.435	0.027	0.435	0.013	0.770
	Exclusive breastfeeding	0.173	0.015	0.033	0.345	0.033	0.345	0.048	0.345
	Family history	0.099	0.126	0.018	0.593	0.018	0.593	0.008	0.841
	Non-AD/AD	0.049	0.138	0.019	0.318	0.019	0.318	0.047	0.106
	Non-AD/ Mild AD/ Moderate to Severe AD	0.132	0.027	0.034	0.376	0.034	0.376	0.079	0.124
	Egg IgE (IU/ml)	0.590	0.466	0.683	0.371	0.683	0.371	0.625	0.160
	Milk IgE (IU/ml)	0.548	0.531	0.573	0.855	0.573	0.855	0.782	0.086
Dataset	Variable	6 months		7-12 months		13-24 months		25-36 months	
		R-squared	*P* value	R-squared	*P* value	R-squared	*P* value	R-squared	*P* value
Genefamily	Sex	0.042	0.430	0.001	0.959	0.022	0.664	0.040	0.428
	Mode of delivery	0.039	0.424	0.018	0.582	0.003	0.950	0.012	0.773
	Exclusive breastfeeding	0.269	0.043	0.081	0.084	0.089	0.154	0.045	0.395
	Family history	0.015	0.714	0.025	0.483	0.068	0.234	0.049	0.345
	Non-AD/AD	0.064	0.061	0.015	0.402	0.098	0.023	0.033	0.237
	Non-AD/ Mild AD/ Moderate to Severe AD	0.113	0.033	0.035	0.366	0.098	0.092	0.049	0.376
	Egg IgE (IU/ml)	0.565	0.594	0.691	0.168	0.827	0.164	0.578	0.399
	Milk IgE (IU/ml)	0.513	0.736	0.642	0.541	0.711	0.273	0.678	0.505

Table S9. Comparison of gene families involved in butyrate metabolism among phenotypic groups at each age. Significantly different gene families were selected using the Dunn's post-hoc test and MaAsLin2 analysis. *P*-value was adjuste9 using the Benjamini–Hochberg method to calculate the *q* value (false discovery rate adjusted *p*-value). N: Non-AD, M: Mild AD, MS: Moderate to Severe AD.

Gene family	6 months					
	Kruskal-Wallis test		Adjusted *P* value (Dunn's Post-hoc test)			Enrichment
	R-squared	*P* value	Non-AD vs. Mild	Non-AD vs. Moderate to Severe	Mild vs. Moderate to Severe	
K00004: butB; (R R)-butanediol dehydrogenase / meso-butanediol dehydrogenase / diacetyl reductase [EC:1.1.1.4 1.1.1.- 1.1.1.303]	2.5213	0.2835	0.2823	0.1996	0.2619	N=M=MS
K00019: bdh; 3-hydroxybutyrate dehydrogenase [EC:1.1.1.30]	0.2161	0.8976	0.5343	0.4291	0.9933	N=M=MS
K00074: paaH, hbd, fadB, mmgB; 3-hydroxybutyryl-CoA dehydrogenase [EC:1.1.1.157]	0.3409	0.8433	0.8489	0.6014	0.4316	M<N<MS
K00100: bdhAB; butanol dehydrogenase [EC:1.1.1.-]	1.1183	0.5717	0.4488	0.4829	0.3492	MS<M<N
K00135: gabD; succinate-semialdehyde dehydrogenase / glutarate-semialdehyde dehydrogenase [EC:1.2.1.16 1.2.1.79 1.2.1.20]	1.2438	0.5369	0.2551	0.3630	0.3986	M<N<MS
K00169: porA; pyruvate ferredoxin oxidoreductase alpha subunit [EC:1.2.7.1]	2.0440	0.3599	0.2372	0.3205	0.3736	MS=M<N
K00170: porB; pyruvate ferredoxin oxidoreductase beta subunit [EC:1.2.7.1]	2.7399	0.2541	0.1745	0.2718	0.3441	MS=M<N
K00171: porD; pyruvate ferredoxin oxidoreductase delta subunit [EC:1.2.7.1]	0.3109	0.8560	0.8674	0.4266	0.5949	N=M=MS
K00172: porC, porG; pyruvate ferredoxin oxidoreductase gamma subunit [EC:1.2.7.1]	1.2170	0.5442	0.2753	0.3516	0.4616	N=M=MS
K00174: korA, oorA, oforA; 2-oxoglutarate/2-oxoacid ferredoxin oxidoreductase subunit alpha [EC:1.2.7.3 1.2.7.11]	3.9628	0.1379	0.4984	0.0818	0.0695	MS<N<M
K00175: korB, oorB, oforB; 2-oxoglutarate/2-oxoacid ferredoxin oxidoreductase subunit beta [EC:1.2.7.3 1.2.7.11]	2.5877	0.2742	0.1873	0.2502	0.3707	MS<M<N
K00209: fabV, ter; enoyl-[acyl-carrier protein] reductase / trans-2-enoyl-CoA reductase (NAD+) [EC:1.3.1.9 1.3.1.44]	0.0634	0.9688	1.0000	0.4842	0.6568	N=M=MS
K00239: sdhA, frdA; succinate dehydrogenase / fumarate reductase flavoprotein subunit [EC:1.3.5.1 1.3.5.4]	0.2204	0.8957	1.0000	0.5434	0.4959	MS<M<N
K00240: sdhB, frdB; succinate dehydrogenase / fumarate reductase iron-sulfur subunit [EC:1.3.5.1 1.3.5.4]	1.8623	0.3941	0.2088	0.3010	0.3001	M<N<MS
K00241: sdhC, frdC; succinate dehydrogenase / fumarate reductase cytochrome b subunit	3.1722	0.2047	0.4503	0.1382	0.0880	MS<N<M
K00242: sdhD, frdD; succinate dehydrogenase / fumarate reductase membrane anchor subunit	0.2289	0.8919	0.9487	0.4555	0.5922	N<MS<M
K00244: frdA; fumarate reductase flavoprotein subunit [EC:1.3.5.4]	5.2919	0.0709	0.1279	0.0487	0.2052	MS<M<N
K00245: frdB; fumarate reductase iron-sulfur subunit [EC:1.3.5.4]	4.3840	0.1117	0.3144	0.0489	0.0718	MS<N<M
K00246: frdC; fumarate reductase subunit C	0.7918	0.6731	0.4288	0.3022	0.5657	MS<N<M
K00247: frdD; fumarate reductase subunit D	0.0543	0.9732	0.4761	1.0000	0.6560	M<N<MS
K00248: bcd; butyryl-CoA dehydrogenase [EC:1.3.8.1]	4.0284	0.1334	0.2839	0.0672	0.1410	MS=M<N
K00626: atoB; acetyl-CoA C-acetyltransferase [EC:2.3.1.9]	0.2686	0.8743	0.4422	0.4932	0.9280	MS<M<N
K00634: ptb; phosphate butyryltransferase [EC:2.3.1.19]	2.6546	0.2652	0.2180	0.1780	0.1561	MS<N<M
K00656: pflD; formate C-acetyltransferase [EC:2.3.1.54]	0.8864	0.6420	0.4026	0.5964	0.3943	MS<M<N
K00823: puuE; 4-aminobutyrate aminotransferase [EC:2.6.1.19]	0.2169	0.8972	0.5726	0.3953	0.9668	N<MS<M
K00929: buk; butyrate kinase [EC:2.7.2.7]	2.8737	0.2377	0.1569	0.2011	0.3335	MS<M<N
K01028: scoA; 3-oxoacid CoA-transferase subunit A [EC:2.8.3.5]	1.0773	0.5835	0.4647	0.4933	0.2682	MS<M<N
K01029: scoB; 3-oxoacid CoA-transferase subunit B [EC:2.8.3.5]	0.1290	0.9375	0.5998	0.4285	1.0000	MS=N<M
K01034: atoD; acetate CoA/acetoacetate CoA-transferase alpha subunit [EC:2.8.3.8 2.8.3.9]	1.0380	0.5951	0.2834	0.3953	0.5491	N<MS<M
K01035: atoA, but; acetate CoA/acetoacetate CoA-transferase beta subunit [EC:2.8.3.8 2.8.3.9]	1.4405	0.4866	0.3775	0.4809	0.2700	N<MS<M
K01039: gctA; glutaconate CoA-transferase subunit A [EC:2.8.3.12]	3.0769	0.2147	0.1352	0.5000	0.1474	N=M=MS
K01040: gctB; glutaconate CoA-transferase subunit B [EC:2.8.3.12]	0.8906	0.6406	0.4384	0.5213	0.3619	N=M=MS
K01575: alsD, budA, aldC; acetolactate decarboxylase [EC:4.1.1.5]	2.0793	0.3536	0.2188	0.2487	0.2441	M<N<MS
K01580: gadB, gadA; glutamate decarboxylase [EC:4.1.1.15]	3.4533	0.1779	0.0990	0.2305	0.3368	M<MS<N
K01615: gcdA; glutaconyl-CoA decarboxylase subunit alpha [EC:7.2.4.5]	6.6734	0.0356	0.4398	0.0171	0.0272	MS<M<N
K01640: hmgL; hydroxymethylglutaryl-CoA lyase [EC:4.1.3.4]	0.8660	0.6486	0.3771	0.3260	0.5486	N=M=MS

K01641: hydroxymethylglutaryl-CoA synthase [EC:2.3.3.10]	0.2187	0.8964	0.9607	0.4612	0.5899	MS<N<M
K01652: ilvB, ilvG, ilvI; acetolactate synthase I/II/III large subunit [EC:2.2.1.6]	1.0709	0.5854	0.3119	0.3416	0.4933	M<N<MS
K01653: ilvH, ilvN; acetolactate synthase I/III small subunit [EC:2.2.1.6]	1.9046	0.3858	0.2074	0.2901	0.2527	N<M<MS
K01692: paaF, echA; enoyl-CoA hydratase [EC:4.2.1.17]	3.0747	0.2149	0.1193	0.3165	0.2686	MS=M<N
K01715: crt; enoyl-CoA hydratase [EC:4.2.1.17]	1.1634	0.5589	0.2874	0.3488	0.4732	N=M=MS
K01782: fadJ; 3-hydroxyacyl-CoA dehydrogenase / enoyl-CoA hydratase / 3-hydroxybutyryl-CoA epimerase [EC:1.1.1.35 4.2.1.17 5.1.2.3]	6.9865	0.0304	0.2001	0.0125	0.0723	MS<M<N
K01825: fadB; 3-hydroxyacyl-CoA dehydrogenase / enoyl-CoA hydratase / 3-hydroxybutyryl-CoA epimerase / enoyl-CoA isomerase [EC:1.1.1.35 4.2.1.17 5.1.2.3 5.3.3.8]	3.2093	0.2010	0.1860	0.1576	0.2964	N=M=MS
K03366: butA, budC; meso-butanediol dehydrogenase / (S S)-butanediol dehydrogenase / diacetyl reductase [EC:1.1.1.- 1.1.1.76 1.1.1.304]	0.9509	0.6216	0.3074	0.3854	0.5664	M<N<MS
K03737: por, nifJ; pyruvate-ferredoxin/flavodoxin oxidoreductase [EC:1.2.7.1 1.2.7.-]	4.1925	0.1229	0.4187	0.0832	0.0506	MS<M<N
K04072: adhE; acetaldehyde dehydrogenase / alcohol dehydrogenase [EC:1.2.1.10 1.1.1.1]	0.1858	0.9113	0.5873	0.4015	1.0000	MS<N<M
K04073: mhpF; acetaldehyde dehydrogenase [EC:1.2.1.10]	0.7810	0.6767	0.4414	0.2961	0.5683	MS<N<M
K07246: ttuC, dmlA; tartrate dehydrogenase/decarboxylase / D-malate dehydrogenase [EC:1.1.1.93 4.1.1.73 1.1.1.83]	0.0543	0.9732	0.4760	1.0000	0.6560	MS<M<N
K07250: gabT; 4-aminobutyrate aminotransferase / (S)-3-amino-2-methylpropionate transaminase / 5-aminovalerate transaminase [EC:2.6.1.19 2.6.1.22 2.6.1.48]	2.0117	0.3657	0.3079	0.1869	0.2474	MS<N<M
K08318: yihU; 4-hydroxybutyrate dehydrogenase / sulfolactaldehyde 3-reductase [EC:1.1.1.61 1.1.1.373]	0.8333	0.6593	0.4218	0.3133	0.5865	MS<N<M
K08324: sad; succinate-semialdehyde dehydrogenase [EC:1.2.1.16 1.2.1.24]	0.0034	0.9983	1.0000	0.7344	0.4919	N<M<MS
K11258: ilvM; acetolactate synthase II small subunit [EC:2.2.1.6]	0.7895	0.6738	0.5614	0.4094	0.4750	N<MS<M
K14534: abfD; 4-hydroxybutyryl-CoA dehydratase / vinylacetyl-CoA-Delta-isomerase [EC:4.2.1.120 5.3.3.3]	0.2758	0.8712	0.4636	0.9012	0.5239	N=M=MS
K17829: ccrA; crotonyl-CoA reductase [EC:1.3.1.86]	NA	NA	NA	NA	NA	N=M=MS
K17865: croR; 3-hydroxybutyryl-CoA dehydratase [EC:4.2.1.55]	3.0769	0.2147	0.1352	0.5000	0.1474	N=M=MS
K18118: aarC, cat1; succinyl-CoA:acetate CoA-transferase [EC:2.8.3.18]	5.6848	0.0583	0.2545	0.0308	0.0333	MS<N<M
K18120: 4hbD, abfH; 4-hydroxybutyrate dehydrogenase [EC:1.1.1.61]	0.6873	0.7092	0.4340	0.3258	0.6205	N=M=MS
K18122: cat2, abfT; 4-hydroxybutyrate CoA-transferase [EC:2.8.3.-]	3.0769	0.2147	0.1352	0.5000	0.1474	N=M=MS
Gene family	7-12 months					Enrichment
	Kruskal-Wallis test		Adjusted *P* value (Dunn's Post-hoc test)			
	R-squared	*P* value	Non-AD vs. Mild	Non-AD vs. Moderate to Severe	Mild vs. Moderate to Severe	
K00004: butB; (R R)-butanediol dehydrogenase / meso-butanediol dehydrogenase / diacetyl reductase [EC:1.1.1.4 1.1.1.- 1.1.1.303]	0.3491	0.8398	0.8413	0.5398	0.3883	N=M=MS
K00019: bdh; 3-hydroxybutyrate dehydrogenase [EC:1.1.1.30]	6.8798	0.0321	0.0117	0.0202	0.4224	MS=M<N
K00074: paaH, hbd, fadB, mmgB; 3-hydroxybutyryl-CoA dehydrogenase [EC:1.1.1.157]	5.4070	0.0670	0.1648	0.0481	0.0680	MS<M<N
K00100: bdhAB; butanol dehydrogenase [EC:1.1.1.-]	0.3922	0.8219	0.8359	0.3875	0.5204	N<MS<M
K00135: gabD; succinate-semialdehyde dehydrogenase / glutarate-semialdehyde dehydrogenase [EC:1.2.1.16 1.2.1.79 1.2.1.20]	0.2862	0.8666	0.9129	0.4721	0.4938	M<N<MS
K00169: porA; pyruvate ferredoxin oxidoreductase alpha subunit [EC:1.2.7.1]	4.9861	0.0827	0.2004	0.3126	0.0428	N<MS<M
K00170: porB; pyruvate ferredoxin oxidoreductase beta subunit [EC:1.2.7.1]	3.7589	0.1527	0.3228	0.2699	0.0804	MS<N<M
K00171: porD; pyruvate ferredoxin oxidoreductase delta subunit [EC:1.2.7.1]	1.3945	0.4980	0.3602	0.4654	0.3566	N=M=MS
K00172: porC, porG; pyruvate ferredoxin oxidoreductase gamma subunit [EC:1.2.7.1]	3.2819	0.1938	0.2648	0.1610	0.1186	MS<M<N
K00174: korA, oorA, oforA; 2-oxoglutarate/2-oxoacid ferredoxin oxidoreductase subunit alpha [EC:1.2.7.3 1.2.7.11]	1.7474	0.4174	0.3035	0.2402	0.3464	N<MS<M
K00175: korB, oorB, oforB; 2-oxoglutarate/2-oxoacid ferredoxin oxidoreductase subunit beta [EC:1.2.7.3 1.2.7.11]	2.1990	0.3330	0.2099	0.1770	0.3691	N<M<MS
K00209: fabV, ter; enoyl-[acyl-carrier protein] reductase / trans-2-enoyl-CoA reductase (NAD+) [EC:1.3.1.9 1.3.1.44]	0.6064	0.7384	0.4428	0.5113	0.6607	MS<M<N
K00239: sdhA, frdA; succinate dehydrogenase / fumarate reductase flavoprotein subunit [EC:1.3.5.1 1.3.5.4]	9.5692	0.0084	0.2285	0.1047	0.0031	MS<N<M
K00240: sdhB, frdB; succinate dehydrogenase / fumarate reductase iron-sulfur subunit [EC:1.3.5.1 1.3.5.4]	7.0831	0.0290	0.0811	0.4030	0.0175	MS<N<M
K00241: sdhC, frdC; succinate dehydrogenase / fumarate reductase cytochrome b subunit	2.3360	0.3110	0.2163	0.2196	0.2795	N<MS<M
K00242: sdhD, frdD; succinate dehydrogenase / fumarate reductase membrane anchor subunit	3.3327	0.1889	0.2935	0.3288	0.1020	M<MS<N

K00244: frdA; fumarate reductase flavoprotein subunit [EC:1.3.5.4]	6.8696	0.0322	0.0802	0.0171	0.0738	MS<M<N
K00245: frdB; fumarate reductase iron-sulfur subunit [EC:1.3.5.4]	0.9882	0.6101	0.5400	0.3394	0.3828	M<MS<N
K00246: frdC; fumarate reductase subunit C	2.0659	0.3560	0.2463	0.4959	0.2855	M<MS<N
K00247: frdD; fumarate reductase subunit D	2.4648	0.2916	0.3423	0.3510	0.1828	M<MS<N
K00248: bcd; butyryl-CoA dehydrogenase [EC:1.3.8.1]	8.1677	0.0168	0.3023	0.0975	0.0070	MS<N<M
K00626: atoB; acetyl-CoA C-acetyltransferase [EC:2.3.1.9]	4.5623	0.1022	0.1472	0.0636	0.1142	MS<M<N
K00634: ptb; phosphate butyryltransferase [EC:2.3.1.19]	2.9023	0.2343	0.3209	0.2201	0.1197	N<M<MS
K00656: pflD; formate C-acetyltransferase [EC:2.3.1.54]	10.3860	0.0056	0.4603	0.0185	0.0038	MS<N<M
K00823: puuE; 4-aminobutyrate aminotransferase [EC:2.6.1.19]	3.0529	0.2173	0.1006	0.1302	0.3530	M<MS<N
K00929: buk; butyrate kinase [EC:2.7.2.7]	0.9728	0.6148	0.4508	0.4270	0.5002	N<M<MS
K01028: scoA; 3-oxoacid CoA-transferase subunit A [EC:2.8.3.5]	0.2311	0.8909	0.4963	0.9712	0.4801	N=M=MS
K01029: scoB; 3-oxoacid CoA-transferase subunit B [EC:2.8.3.5]	0.9229	0.6304	0.2957	0.5298	0.4401	N=M=MS
K01034: atoD; acetate CoA/acetoacetate CoA-transferase alpha subunit [EC:2.8.3.8 2.8.3.9]	0.5919	0.7438	0.5161	0.4417	0.6800	M<N<MS
K01035: atoA, but; acetate CoA/acetoacetate CoA-transferase beta subunit [EC:2.8.3.8 2.8.3.9]	0.6280	0.7305	0.4729	0.4721	0.6617	N<M<MS
K01039: gctA; glutaconate CoA-transferase subunit A [EC:2.8.3.12]	1.1384	0.5660	0.4715	0.2445	0.4932	N=M=MS
K01040: gctB; glutaconate CoA-transferase subunit B [EC:2.8.3.12]	0.4812	0.7862	0.4536	0.4719	0.8205	N=M=MS
K01575: alsD, budA, aldC; acetolactate decarboxylase [EC:4.1.1.5]	3.0633	0.2162	0.4722	0.1943	0.1393	MS<N<M
K01580: gadB, gadA; glutamate decarboxylase [EC:4.1.1.15]	0.8106	0.6668	0.6503	0.3903	0.3773	M<MS<N
K01615: gcdA; glutaconyl-CoA decarboxylase subunit alpha [EC:7.2.4.5]	1.9800	0.3716	0.3260	0.3249	0.2261	N<M<MS
K01640: hmgL; hydroxymethylglutaryl-CoA lyase [EC:4.1.3.4]	0.3276	0.8489	0.4279	0.6124	0.8506	N=M=MS
K01641: hydroxymethylglutaryl-CoA synthase [EC:2.3.3.10]	0.3675	0.8321	0.5157	0.8199	0.3812	MS<N<M
K01652: ilvB, ilvG, ilvI; acetolactate synthase I/II/III large subunit [EC:2.2.1.6]	7.8430	0.0198	0.1774	0.2034	0.0077	MS<N<M
K01653: ilvH, ilvN; acetolactate synthase I/III small subunit [EC:2.2.1.6]	7.2492	0.0267	0.3041	0.1200	0.0114	MS<N<M
K01692: paaF, echA; enoyl-CoA hydratase [EC:4.2.1.17]	5.0314	0.0808	0.0373	0.0749	0.2337	MS=M<N
K01715: crt; enoyl-CoA hydratase [EC:4.2.1.17]	5.9177	0.0519	0.1100	0.0303	0.0818	MS<M<N
K01782: fadJ; 3-hydroxyacyl-CoA dehydrogenase / enoyl-CoA hydratase / 3-hydroxybutyryl-CoA epimerase [EC:1.1.1.35 4.2.1.17 5.1.2.3]	4.4828	0.1063	0.0566	0.1557	0.1481	M<MS<N
K01825: fadB; 3-hydroxyacyl-CoA dehydrogenase / enoyl-CoA hydratase / 3-hydroxybutyryl-CoA epimerase / enoyl-CoA isomerase [EC:1.1.1.35 4.2.1.17 5.1.2.3 5.3.3.8]	4.6820	0.0962	0.0614	0.1515	0.1396	N=M=MS
K03366: butA, budC; meso-butanediol dehydrogenase / (S S)-butanediol dehydrogenase / diacetyl reductase [EC:1.1.1.- 1.1.1.76 1.1.1.304]	0.2229	0.8945	0.4030	0.9755	0.5707	N<M<MS
K03737: por, nifJ; pyruvate-ferredoxin/flavodoxin oxidoreductase [EC:1.2.7.1 1.2.7.-]	0.6155	0.7351	0.6572	0.4674	0.3595	N<M<MS
K04072: adhE; acetaldehyde dehydrogenase / alcohol dehydrogenase [EC:1.2.1.10 1.1.1.1]	7.0998	0.0287	0.1983	0.0297	0.0251	MS<M<N
K04073: mhpF; acetaldehyde dehydrogenase [EC:1.2.1.10]	2.2552	0.3238	0.2219	0.1354	0.4687	MS<M<N
K07246: ttuC, dmlA; tartrate dehydrogenase/decarboxylase / D-malate dehydrogenase [EC:1.1.1.93 4.1.1.73 1.1.1.83]	2.6100	0.2712	0.1684	0.1274	0.4119	M<MS<N
K07250: gabT; 4-aminobutyrate aminotransferase / (S)-3-amino-2-methylpropionate transaminase / 5-aminovalerate transaminase [EC:2.6.1.19 2.6.1.22 2.6.1.48]	3.0193	0.2210	0.3403	0.1118	0.2123	N<M<MS
K08318: yihU; 4-hydroxybutyrate dehydrogenase / sulfolactaldehyde 3-reductase [EC:1.1.1.61 1.1.1.373]	0.7388	0.6911	0.3843	0.4569	0.6929	MS<M<N
K08324: sad; succinate-semialdehyde dehydrogenase [EC:1.2.1.16 1.2.1.24]	3.4127	0.1815	0.2820	0.3298	0.1035	M<N<MS
K11258: ilvM; acetolactate synthase II small subunit [EC:2.2.1.6]	9.0598	0.0108	0.1004	0.2563	0.0048	M<N<MS
K14534: abfD; 4-hydroxybutyryl-CoA dehydratase / vinylacetyl-CoA-Delta-isomerase [EC:4.2.1.120 5.3.3.3]	7.8634	0.0196	0.0121	0.0075	0.4894	N=M=MS
K17829: ccrA; crotonyl-CoA reductase [EC:1.3.1.86]	NA	NA	NA	NA	NA	N=M=MS
K17865: croR; 3-hydroxybutyryl-CoA dehydratase [EC:4.2.1.55]	NA	NA	NA	NA	NA	N=M=MS
K18118: aarC, cat1; succinyl-CoA:acetate CoA-transferase [EC:2.8.3.18]	1.2444	0.5368	0.2858	0.4284	0.5261	N<M<MS
K18120: 4hbD, abfH; 4-hydroxybutyrate dehydrogenase [EC:1.1.1.61]	5.0774	0.0790	0.3228	0.1879	0.0379	N=M=MS
K18122: cat2, abfT; 4-hydroxybutyrate CoA-transferase [EC:2.8.3.-]	1.7627	0.4142	0.1851	0.2942	0.4022	N=M=MS
	13-24 months					

Gene family	Kruskal-Wallis test		Adjusted *P* value (Dunn's Post-hoc test)			Enrichment
	R-squared	*P* value	Non-AD vs. Mild	Non-AD vs. Moderate to Severe	Mild vs. Moderate to Severe	
K00004: butB; (R R)-butanediol dehydrogenase / meso-butanediol dehydrogenase / diacetyl reductase [EC:1.1.1.4 1.1.1.- 1.1.1.303]	0.7095	0.7013	0.3285	0.6265	0.4988	N=M=MS
K00019: bdh; 3-hydroxybutyrate dehydrogenase [EC:1.1.1.30]	2.7594	0.2517	0.1500	0.1152	0.3319	N=M=MS
K00074: paaH, hbd, fadB, mmgB; 3-hydroxybutyryl-CoA dehydrogenase [EC:1.1.1.157]	3.8383	0.1467	0.2757	0.3608	0.0767	M<N<MS
K00100: bdhAB; butanol dehydrogenase [EC:1.1.1.-]	0.2808	0.8690	0.4466	0.6215	0.9003	M<MS<N
K00135: gabD; succinate-semialdehyde dehydrogenase / glutarate-semialdehyde dehydrogenase [EC:1.2.1.16 1.2.1.79 1.2.1.20]	2.5321	0.2819	0.1125	0.1829	0.4774	N<MS<M
K00169: porA; pyruvate ferredoxin oxidoreductase alpha subunit [EC:1.2.7.1]	2.8178	0.2444	0.1858	0.4448	0.1733	M<N<MS
K00170: porB; pyruvate ferredoxin oxidoreductase beta subunit [EC:1.2.7.1]	2.3049	0.3159	0.3300	0.4280	0.1970	MS<N<M
K00171: porD; pyruvate ferredoxin oxidoreductase delta subunit [EC:1.2.7.1]	0.9501	0.6219	0.4357	0.4710	0.5088	MS=M<N
K00172: porC, porG; pyruvate ferredoxin oxidoreductase gamma subunit [EC:1.2.7.1]	3.8220	0.1479	0.1157	0.1963	0.1076	M<MS<N
K00174: korA, oorA, oforA; 2-oxoglutarate/2-oxoacid ferredoxin oxidoreductase subunit alpha [EC:1.2.7.3 1.2.7.11]	0.4217	0.8099	0.4513	0.5708	0.7886	MS<M<N
K00175: korB, oorB, oforB; 2-oxoglutarate/2-oxoacid ferredoxin oxidoreductase subunit beta [EC:1.2.7.3 1.2.7.11]	0.4307	0.8063	0.4123	0.7877	0.4948	M<MS<N
K00209: fabV, ter; enoyl-[acyl-carrier protein] reductase / trans-2-enoyl-CoA reductase (NAD+) [EC:1.3.1.9 1.3.1.44]	2.3908	0.3026	0.4475	0.3095	0.1839	M<N<MS
K00239: sdhA, frdA; succinate dehydrogenase / fumarate reductase flavoprotein subunit [EC:1.3.5.1 1.3.5.4]	1.4027	0.4959	0.2764	0.4136	0.4321	N<MS<M
K00240: sdhB, frdB; succinate dehydrogenase / fumarate reductase iron-sulfur subunit [EC:1.3.5.1 1.3.5.4]	0.4866	0.7841	0.5478	0.4557	0.7323	N<MS<M
K00241: sdhC, frdC; succinate dehydrogenase / fumarate reductase cytochrome b subunit	0.6008	0.7405	0.4525	0.6661	0.3728	M<MS<N
K00242: sdhD, frdD; succinate dehydrogenase / fumarate reductase membrane anchor subunit	3.5456	0.1699	0.0908	0.1283	0.1902	N<MS<M
K00244: frdA; fumarate reductase flavoprotein subunit [EC:1.3.5.4]	5.7107	0.0575	0.0479	0.1554	0.0525	M<MS<N
K00245: frdB; fumarate reductase iron-sulfur subunit [EC:1.3.5.4]	3.0361	0.2191	0.1083	0.1244	0.3818	N<MS<M
K00246: frdC; fumarate reductase subunit C	2.9950	0.2237	0.0835	0.1436	0.4989	N<MS<M
K00247: frdD; fumarate reductase subunit D	3.4712	0.1763	0.0876	0.0959	0.3792	N<MS<M
K00248: bcd; butyryl-CoA dehydrogenase [EC:1.3.8.1]	2.5381	0.2811	0.1710	0.2014	0.2100	M<MS<N
K00626: atoB; acetyl-CoA C-acetyltransferase [EC:2.3.1.9]	1.4829	0.4764	0.4043	0.4324	0.3496	M<N<MS
K00634: ptb; phosphate butyryltransferase [EC:2.3.1.19]	0.4023	0.8178	0.8580	0.3973	0.4697	M<MS<N
K00656: pflD; formate C-acetyltransferase [EC:2.3.1.54]	2.9066	0.2338	0.2359	0.1894	0.1914	M<N<MS
K00823: puuE; 4-aminobutyrate aminotransferase [EC:2.6.1.19]	2.4398	0.2953	0.2098	0.2030	0.2203	N<MS<M
K00929: buk; butyrate kinase [EC:2.7.2.7]	3.0703	0.2154	0.1375	0.0802	0.4102	M<MS<N
K01028: scoA; 3-oxoacid CoA-transferase subunit A [EC:2.8.3.5]	1.0199	0.6005	0.4527	0.3535	0.5722	N=M=MS
K01029: scoB; 3-oxoacid CoA-transferase subunit B [EC:2.8.3.5]	0.0650	0.9680	0.4960	0.6595	1.0000	N=M=MS
K01034: atoD; acetate CoA/acetoacetate CoA-transferase alpha subunit [EC:2.8.3.8 2.8.3.9]	1.5933	0.4508	0.4389	0.3797	0.3381	M<N<MS
K01035: atoA, but; acetate CoA/acetoacetate CoA-transferase beta subunit [EC:2.8.3.8 2.8.3.9]	1.7696	0.4128	0.3689	0.4392	0.2826	N<M<MS
K01039: gctA; glutaconate CoA-transferase subunit A [EC:2.8.3.12]	0.1512	0.9272	1.0000	0.4244	0.5875	N=M=MS
K01040: gctB; glutaconate CoA-transferase subunit B [EC:2.8.3.12]	0.4322	0.8057	0.5748	0.4460	0.7677	N<MS<M
K01575: alsD, budA, aldC; acetolactate decarboxylase [EC:4.1.1.5]	0.9098	0.6345	0.4409	0.3601	0.6289	N<MS<M
K01580: gadB, gadA; glutamate decarboxylase [EC:4.1.1.15]	5.7675	0.0559	0.0192	0.0325	0.4756	M<MS<N
K01615: gcdA; glutaconyl-CoA decarboxylase subunit alpha [EC:7.2.4.5]	0.7932	0.6726	0.3115	0.5843	0.4958	N<MS<M
K01640: hmgL; hydroxymethylglutaryl-CoA lyase [EC:4.1.3.4]	5.3306	0.0696	0.0425	0.0320	0.3272	MS=M<N
K01641: hydroxymethylglutaryl-CoA synthase [EC:2.3.3.10]	0.0086	0.9957	0.4976	0.7181	1.0000	M<MS<N
K01652: ilvB, ilvG, ilvI; acetolactate synthase I/II/III large subunit [EC:2.2.1.6]	1.5614	0.4581	0.4643	0.3705	0.3172	M<N<MS
K01653: ilvH, ilvN; acetolactate synthase I/III small subunit [EC:2.2.1.6]	6.3961	0.0408	0.1532	0.3520	0.0181	M<N<MS
K01692: paaF, echA; enoyl-CoA hydratase [EC:4.2.1.17]	0.4496	0.7987	0.5174	0.4895	0.7713	N=M=MS

K01715: crt; enoyl-CoA hydratase [EC:4.2.1.17]	3.2407	0.1978	0.3173	0.3501	0.1084	M<N<MS
K01782: fadJ; 3-hydroxyacyl-CoA dehydrogenase / enoyl-CoA hydratase / 3-hydroxybutyryl-CoA epimerase [EC:1.1.1.35 4.2.1.17 5.1.2.3]	2.2434	0.3257	0.2676	0.2620	0.1926	MS=N<M
K01825: fadB; 3-hydroxyacyl-CoA dehydrogenase / enoyl-CoA hydratase / 3-hydroxybutyryl-CoA epimerase / enoyl-CoA isomerase [EC:1.1.1.35 4.2.1.17 5.1.2.3 5.3.3.8]	3.0256	0.2203	0.1497	0.1759	0.1831	N=M=MS
K03366: butA, budC; meso-butanediol dehydrogenase / (S S)-butanediol dehydrogenase / diacetyl reductase [EC:1.1.1.- 1.1.1.76 1.1.1.304]	6.8778	0.0321	0.2368	0.0487	0.0312	N<M<MS
K03737: por, nifJ; pyruvate-ferredoxin/flavodoxin oxidoreductase [EC:1.2.7.1 1.2.7.-]	1.2001	0.5488	0.4103	0.3043	0.3216	MS<M<N
K04072: adhE; acetaldehyde dehydrogenase / alcohol dehydrogenase [EC:1.2.1.10 1.1.1.1]	3.5991	0.1654	0.1986	0.4634	0.0965	M<N<MS
K04073: mhpF; acetaldehyde dehydrogenase [EC:1.2.1.10]	1.2349	0.5393	0.4146	0.3591	0.2630	N<MS<M
K07246: ttuC, dmlA; tartrate dehydrogenase/decarboxylase / D-malate dehydrogenase [EC:1.1.1.93 4.1.1.73 1.1.1.83]	4.7787	0.0917	0.0623	0.3025	0.0849	MS<N<M
K07250: gabT; 4-aminobutyrate aminotransferase / (S)-3-amino-2-methylpropionate transaminase / 5-aminovalerate transaminase [EC:2.6.1.19 2.6.1.22 2.6.1.48]	1.8292	0.4007	0.2680	0.3249	0.2915	N<M<MS
K08318: yihU; 4-hydroxybutyrate dehydrogenase / sulfolactaldehyde 3-reductase [EC:1.1.1.61 1.1.1.373]	2.6052	0.2718	0.1687	0.1165	0.3616	N<MS<M
K08324: sad; succinate-semialdehyde dehydrogenase [EC:1.2.1.16 1.2.1.24]	4.3709	0.1124	0.1688	0.0840	0.1375	N<M<MS
K11258: ilvM; acetolactate synthase II small subunit [EC:2.2.1.6]	4.9226	0.0853	0.0596	0.1399	0.0864	N<MS<M
K14534: abfD; 4-hydroxybutyryl-CoA dehydratase / vinylacetyl-CoA-Delta-isomerase [EC:4.2.1.120 5.3.3.3]	20.9982	0.0000	0.0002	0.0000	0.1484	MS=M<N
K17829: ccrA; crotonyl-CoA reductase [EC:1.3.1.86]	1.8750	0.3916	0.2933	0.5000	0.2809	N=M=MS
K17865: croR; 3-hydroxybutyryl-CoA dehydratase [EC:4.2.1.55]	7.3636	0.0252	0.0095	0.0134	0.5000	N=M=MS
K18118: aarC, cat1; succinyl-CoA:acetate CoA-transferase [EC:2.8.3.18]	0.1771	0.9153	0.5865	1.0000	0.4276	M<N<MS
K18120: 4hbD, abfH; 4-hydroxybutyrate dehydrogenase [EC:1.1.1.61]	0.6741	0.7139	0.7210	0.3873	0.3800	N=M=MS
K18122: cat2, abfT; 4-hydroxybutyrate CoA-transferase [EC:2.8.3.-]	4.9872	0.0826	0.4556	0.1034	0.0672	N=M=MS

Gene family	25-36 months					
	Kruskal-Wallis test		Adjusted *P* value (Dunn's Post-hoc test)			Enrichment
	R-squared	*P* value	Non-AD vs. Mild	Non-AD vs. Moderate to Severe	Mild vs. Moderate to Severe	
K00004: butB; (R R)-butanediol dehydrogenase / meso-butanediol dehydrogenase / diacetyl reductase [EC:1.1.1.4 1.1.1.- 1.1.1.303]	1.6049	0.4482	0.2259	0.3215	0.3227	N=M=MS
K00019: bdh; 3-hydroxybutyrate dehydrogenase [EC:1.1.1.30]	0.1384	0.9331	1.0000	0.4749	0.5737	N=M=MS
K00074: paaH, hbd, fadB, mmgB; 3-hydroxybutyryl-CoA dehydrogenase [EC:1.1.1.157]	0.9441	0.6237	0.5939	0.3748	0.3744	N<MS<M
K00100: bdhAB; butanol dehydrogenase [EC:1.1.1.-]	1.3380	0.5122	0.2254	0.4079	0.3963	M<MS<N
K00135: gabD; succinate-semialdehyde dehydrogenase / glutarate-semialdehyde dehydrogenase [EC:1.2.1.16 1.2.1.79 1.2.1.20]	1.6196	0.4449	0.2529	0.2852	0.3348	MS<N<M
K00169: porA; pyruvate ferredoxin oxidoreductase alpha subunit [EC:1.2.7.1]	8.0314	0.0180	0.4363	0.0083	0.0588	MS<N<M
K00170: porB; pyruvate ferredoxin oxidoreductase beta subunit [EC:1.2.7.1]	12.5514	0.0019	0.0131	0.0268	0.0006	MS<N<M
K00171: porD; pyruvate ferredoxin oxidoreductase delta subunit [EC:1.2.7.1]	5.8089	0.0548	0.3515	0.0247	0.1461	MS<M<N
K00172: porC, porG; pyruvate ferredoxin oxidoreductase gamma subunit [EC:1.2.7.1]	5.0826	0.0788	0.2447	0.0365	0.2723	MS<N<M
K00174: korA, oorA, oforA; 2-oxoglutarate/2-oxoacid ferredoxin oxidoreductase subunit alpha [EC:1.2.7.3 1.2.7.11]	8.6554	0.0132	0.0092	0.1978	0.0056	M<N<MS
K00175: korB, oorB, oforB; 2-oxoglutarate/2-oxoacid ferredoxin oxidoreductase subunit beta [EC:1.2.7.3 1.2.7.11]	5.8264	0.0543	0.0240	0.2203	0.0675	M<MS<N
K00209: fabV, ter; enoyl-[acyl-carrier protein] reductase / trans-2-enoyl-CoA reductase (NAD+) [EC:1.3.1.9 1.3.1.44]	1.3247	0.5156	0.4447	0.3925	0.3906	M<MS<N
K00239: sdhA, frdA; succinate dehydrogenase / fumarate reductase flavoprotein subunit [EC:1.3.5.1 1.3.5.4]	2.1254	0.3455	0.4238	0.2238	0.3227	N<M<MS
K00240: sdhB, frdB; succinate dehydrogenase / fumarate reductase iron-sulfur subunit [EC:1.3.5.1 1.3.5.4]	2.8402	0.2417	0.2620	0.1342	0.1955	M<N<MS
K00241: sdhC, frdC; succinate dehydrogenase / fumarate reductase cytochrome b subunit	3.8380	0.1468	0.0884	0.4931	0.0592	M<MS<N
K00242: sdhD, frdD; succinate dehydrogenase / fumarate reductase membrane anchor subunit	0.5478	0.7604	0.7314	0.4721	0.3679	MS=N<M
K00244: frdA; fumarate reductase flavoprotein subunit [EC:1.3.5.4]	1.7636	0.4140	0.2869	0.4573	0.1927	N<MS<M
K00245: frdB; fumarate reductase iron-sulfur subunit [EC:1.3.5.4]	0.7520	0.6866	0.3463	0.3996	0.5890	MS<N<M
K00246: frdC; fumarate reductase subunit C	0.2681	0.8745	0.6252	0.9137	0.4375	M<MS<N
K00247: frdD; fumarate reductase subunit D	0.4960	0.7803	0.7618	0.5157	0.3687	MS<N<M
K00248: bcd; butyryl-CoA dehydrogenase [EC:1.3.8.1]	0.0064	0.9968	0.7205	0.4806	1.0000	M<MS<N

K00626: atoB; acetyl-CoA C-acetyltransferase [EC:2.3.1.9]	0.6641	0.7174	0.4708	0.6328	0.4710	M<N<MS
K00634: ptb; phosphate butyryltransferase [EC:2.3.1.19]	11.8757	0.0026	0.0009	0.2768	0.0043	M<N<MS
K00656: pflD; formate C-acetyltransferase [EC:2.3.1.54]	5.0451	0.0803	0.0852	0..0759	0.2634	N<MS<M
K00823: puuE; 4-aminobutyrate aminotransferase [EC:2.6.1.19]	3.6971	0.1575	0.1319	0.1654	0.0912	MS<N<M
K00929: buk; butyrate kinase [EC:2.7.2.7]	8.0420	0.0179	0.0073	0.1484	0.0446	M<MS<N
K01028: scoA; 3-oxoacid CoA-transferase subunit A [EC:2.8.3.5]	1.8468	0.3972	0.2365	0.3796	0.4436	N=M=MS
K01029: scoB; 3-oxoacid CoA-transferase subunit B [EC:2.8.3.5]	0.4515	0.7979	0.7550	0.4545	0.4420	N=M=MS
K01034: atoD; acetate CoA/acetoacetate CoA-transferase alpha subunit [EC:2.8.3.8 2.8.3.9]	0.5670	0.7532	0.3672	0.4555	0.7117	MS<N<M
K01035: atoA, but; acetate CoA/acetoacetate CoA-transferase beta subunit [EC:2.8.3.8 2.8.3.9]	5.1569	0.0759	0.3534	0.0356	0.1705	MS<M<N
K01039: gctA; glutaconate CoA-transferase subunit A [EC:2.8.3.12]	0.4073	0.8158	0.3527	0.5170	0.7988	N=M=MS
K01040: gctB; glutaconate CoA-transferase subunit B [EC:2.8.3.12]	0.3609	0.8349	0.4626	0.4236	0.8279	MS<N<M
K01575: alsD, budA, aldC; acetolactate decarboxylase [EC:4.1.1.5]	4.8994	0.0863	0.0513	0.4689	0.0333	MS<N<M
K01580: gadB, gadA; glutamate decarboxylase [EC:4.1.1.15]	5.7059	0.0577	0.0354	0.1401	0.1183	M<MS<N
K01615: gcdA; glutaconyl-CoA decarboxylase subunit alpha [EC:7.2.4.5]	5.9183	0.0519	0.4395	0.0256	0.1003	N<M<MS
K01640: hmgL; hydroxymethylglutaryl-CoA lyase [EC:4.1.3.4]	3.3884	0.1837	0.2742	0.0995	0.3593	N<M<MS
K01641: hydroxymethylglutaryl-CoA synthase [EC:2.3.3.10]	5.2090	0.0739	0.4551	0.0384	0.1145	MS<M<N
K01652: ilvB, ilvG, ilvI; acetolactate synthase I/II/III large subunit [EC:2.2.1.6]	0.2748	0.8716	1.0000	0.5169	0.4512	N<MS<M
K01653: ilvH, ilvN; acetolactate synthase I/III small subunit [EC:2.2.1.6]	1.0586	0.5890	0.4196	0.5343	0.3083	MS<N<M
K01692: paaF, echA; enoyl-CoA hydratase [EC:4.2.1.17]	1.3849	0.5003	0.4134	0.3590	0.3015	N=M=MS
K01715: crt; enoyl-CoA hydratase [EC:4.2.1.17]	0.2830	0.8680	0.4960	0.9082	0.5470	M<N<MS
K01782: fadJ; 3-hydroxyacyl-CoA dehydrogenase / enoyl-CoA hydratase / 3-hydroxybutyryl-CoA epimerase [EC:1.1.1.35 4.2.1.17 5.1.2.3]	1.1957	0.5500	0.3713	0.4751	0.4769	N=M=MS
K01825: fadB; 3-hydroxyacyl-CoA dehydrogenase / enoyl-CoA hydratase / 3-hydroxybutyryl-CoA epimerase / enoyl-CoA isomerase [EC:1.1.1.35 4.2.1.17 5.1.2.3 5.3.3.8]	0.5438	0.7619	0.3383	0.4731	0.7112	N=M=MS
K03366: butA, budC; meso-butanediol dehydrogenase / (S S)-butanediol dehydrogenase / diacetyl reductase [EC:1.1.1.- 1.1.1.76 1.1.1.304]	4.1184	0.1276	0.2534	0.0693	0.1150	MS<N<M
K03737: por, nifJ; pyruvate-ferredoxin/flavodoxin oxidoreductase [EC:1.2.7.1 1.2.7.-]	1.8348	0.3996	0.2853	0.3320	0.4878	MS<N<M
K04072: adhE; acetaldehyde dehydrogenase / alcohol dehydrogenase [EC:1.2.1.10 1.1.1.1]	7.3661	0.0251	0.0167	0.0852	0.1044	N<MS<M
K04073: mhpF; acetaldehyde dehydrogenase [EC:1.2.1.10]	0.1685	0.9192	0.4787	1.0000	0.5672	MS<N<M
K07246: ttuC, dmlA; tartrate dehydrogenase/decarboxylase / D-malate dehydrogenase [EC:1.1.1.93 4.1.1.73 1.1.1.83]	1.0899	0.5799	0.3880	0.4448	0.4869	MS=M<N
K07250: gabT; 4-aminobutyrate aminotransferase / (S)-3-amino-2-methylpropionate transaminase / 5-aminovalerate transaminase [EC:2.6.1.19 2.6.1.22 2.6.1.48]	0.4739	0.7890	0.7809	0.5146	0.3761	M<N<MS
K08318: yihU; 4-hydroxybutyrate dehydrogenase / sulfolactaldehyde 3-reductase [EC:1.1.1.61 1.1.1.373]	1.8532	0.3959	0.4761	0.2910	0.2571	N=M<MS
K08324: sad; succinate-semialdehyde dehydrogenase [EC:1.2.1.16 1.2.1.24]	0.4503	0.7984	0.3793	0.4641	0.7970	MS<N<M
K11258: ilvM; acetolactate synthase II small subunit [EC:2.2.1.6]	0.0899	0.9561	1.0000	0.4626	0.6241	N=M=MS
K14534: abfD; 4-hydroxybutyryl-CoA dehydratase / vinylacetyl-CoA-Delta-isomerase [EC:4.2.1.120 5.3.3.3]	3.4684	0.1765	0.2002	0.1254	0.4349	N=M=MS
K17829: ccrA; crotonyl-CoA reductase [EC:1.3.1.86]	1.5432	0.4623	0.3068	0.4010	0.5000	N=M=MS
K17865: croR; 3-hydroxybutyryl-CoA dehydratase [EC:4.2.1.55]	0.7636	0.6826	0.4211	0.6528	0.5000	N=M=MS
K18118: aarC, cat1; succinyl-CoA:acetate CoA-transferase [EC:2.8.3.18]	9.1675	0.0102	0.2611	0.0105	0.0109	M<N<MS
K18120: 4hbD, abfH; 4-hydroxybutyrate dehydrogenase [EC:1.1.1.61]	0.9301	0.6281	0.6652	0.3342	0.4272	N=M=MS
K18122: cat2, abfT; 4-hydroxybutyrate CoA-transferase [EC:2.8.3.-]	4.5319	0.1037	0.0796	0.1313	0.1964	N=M=MS

Gene family	MaAsLin2 analysis		
		q value	
	Time point	Non-AD vs. Mild	Non-AD vs. Moderate to Severe
K00004: butB; (R R)-butanediol dehydrogenase / meso-butanediol dehydrogenase / diacetyl reductase [EC:1.1.1.4 1.1.1.- 1.1.1.303]	N.S.*	N.S.	N.S.
K00019: bdh; 3-hydroxybutyrate dehydrogenase [EC:1.1.1.30]	N.S.	N.S.	N.S.

K00074: paaH, hbd, fadB, mmgB; 3-hydroxybutyryl-CoA dehydrogenase [EC:1.1.1.157]	N.S.	N.S.	N.S.
K00100: bdhAB; butanol dehydrogenase [EC:1.1.1.-]	N.S.	N.S.	N.S.
K00135: gabD; succinate-semialdehyde dehydrogenase / glutarate-semialdehyde dehydrogenase [EC:1.2.1.16 1.2.1.79 1.2.1.20]	N.S.	N.S.	N.S.
K00169: porA; pyruvate ferredoxin oxidoreductase alpha subunit [EC:1.2.7.1]	N.S.	N.S.	N.S.
K00170: porB; pyruvate ferredoxin oxidoreductase beta subunit [EC:1.2.7.1]	25-36 months	0.0755	N.S.
K00171: porD; pyruvate ferredoxin oxidoreductase delta subunit [EC:1.2.7.1]	N.S.	N.S.	N.S.
K00172: porC, porG; pyruvate ferredoxin oxidoreductase gamma subunit [EC:1.2.7.1]	N.S.	N.S.	N.S.
K00174: korA, oorA, oforA; 2-oxoglutarate/2-oxoacid ferredoxin oxidoreductase subunit alpha [EC:1.2.7.3 1.2.7.11]	25-36 months	0.2128	N.S.
K00175: korB, oorB, oforB; 2-oxoglutarate/2-oxoacid ferredoxin oxidoreductase subunit beta [EC:1.2.7.3 1.2.7.11]	N.S.	N.S.	N.S.
K00209: fabV, ter; enoyl-[acyl-carrier protein] reductase / trans-2-enoyl-CoA reductase (NAD+) [EC:1.3.1.9 1.3.1.44]	N.S.	N.S.	N.S.
K00239: sdhA, frdA; succinate dehydrogenase / fumarate reductase flavoprotein subunit [EC:1.3.5.1 1.3.5.4]	N.S.	N.S.	N.S.
K00240: sdhB, frdB; succinate dehydrogenase / fumarate reductase iron-sulfur subunit [EC:1.3.5.1 1.3.5.4]	7-12 months	0.2002	N.S.
K00241: sdhC, frdC; succinate dehydrogenase / fumarate reductase cytochrome b subunit	N.S.	N.S.	N.S.
K00242: sdhD, frdD; succinate dehydrogenase / fumarate reductase membrane anchor subunit	N.S.	N.S.	N.S.
K00244: frdA; fumarate reductase flavoprotein subunit [EC:1.3.5.4]	N.S.	N.S.	N.S.
K00245: frdB; fumarate reductase iron-sulfur subunit [EC:1.3.5.4]	N.S.	N.S.	N.S.
K00246: frdC; fumarate reductase subunit C	N.S.	N.S.	N.S.
K00247: frdD; fumarate reductase subunit D	N.S.	N.S.	N.S.
K00248: bcd; butyryl-CoA dehydrogenase [EC:1.3.8.1]	N.S.	N.S.	N.S.
K00626: atoB; acetyl-CoA C-acetyltransferase [EC:2.3.1.9]	N.S.	N.S.	N.S.
K00634: ptb; phosphate butyryltransferase [EC:2.3.1.19]	25-36 months	0.0400	N.S.
K00656: pflD; formate C-acetyltransferase [EC:2.3.1.54]	N.S.	N.S.	N.S.
K00823: puuE; 4-aminobutyrate aminotransferase [EC:2.6.1.19]	N.S.	N.S.	N.S.
K00929: buk; butyrate kinase [EC:2.7.2.7]	25-36 months	0.0898	N.S.
K01028: scoA; 3-oxoacid CoA-transferase subunit A [EC:2.8.3.5]	N.S.	N.S.	N.S.
K01029: scoB; 3-oxoacid CoA-transferase subunit B [EC:2.8.3.5]	N.S.	N.S.	N.S.
K01034: atoD; acetate CoA/acetoacetate CoA-transferase alpha subunit [EC:2.8.3.8 2.8.3.9]	N.S.	N.S.	N.S.
K01035: atoA, but; acetate CoA/acetoacetate CoA-transferase beta subunit [EC:2.8.3.8 2.8.3.9]	N.S.	N.S.	N.S.
K01039: gctA; glutaconate CoA-transferase subunit A [EC:2.8.3.12]	N.S.	N.S.	N.S.
K01040: gctB; glutaconate CoA-transferase subunit B [EC:2.8.3.12]	N.S.	N.S.	N.S.
K01575: alsD, budA, aldC; acetolactate decarboxylase [EC:4.1.1.5]	N.S.	N.S.	N.S.
K01580: gadB, gadA; glutamate decarboxylase [EC:4.1.1.15]	13-24 months	N.S.	0.2077
K01615: gcdA; glutaconyl-CoA decarboxylase subunit alpha [EC:7.2.4.5]	N.S.	N.S.	N.S.
K01640: hmgL; hydroxymethylglutaryl-CoA lyase [EC:4.1.3.4]	N.S.	N.S.	N.S.
K01641: hydroxymethylglutaryl-CoA synthase [EC:2.3.3.10]	N.S.	N.S.	N.S.
K01652: ilvB, ilvG, ilvI; acetolactate synthase I/II/III large subunit [EC:2.2.1.6]	N.S.	N.S.	N.S.
K01653: ilvH, ilvN; acetolactate synthase I/III small subunit [EC:2.2.1.6]	N.S.	N.S.	N.S.
K01692: paaF, echA; enoyl-CoA hydratase [EC:4.2.1.17]	N.S.	N.S.	N.S.
K01715: crt; enoyl-CoA hydratase [EC:4.2.1.17]	N.S.	N.S.	N.S.
K01782: fadJ; 3-hydroxyacyl-CoA dehydrogenase / enoyl-CoA hydratase / 3-hydroxybutyryl-CoA epimerase [EC:1.1.1.35 4.2.1.17 5.1.2.3]	N.S.	N.S.	N.S.
K01825: fadB; 3-hydroxyacyl-CoA dehydrogenase / enoyl-CoA hydratase / 3-hydroxybutyryl-CoA epimerase / enoyl-CoA isomerase [EC:1.1.1.35 4.2.1.17 5.1.2.3 5.3.3.8]	N.S.	N.S.	N.S.
K03366: butA, budC; meso-butanediol dehydrogenase / (S S)-butanediol dehydrogenase / diacetyl reductase [EC:1.1.1.- 1.1.1.76 1.1.1.304]	N.S.	N.S.	N.S.
K03737: por, nifJ; pyruvate-ferredoxin/flavodoxin oxidoreductase [EC:1.2.7.1 1.2.7.-]	N.S.	N.S.	N.S.
K04072: adhE; acetaldehyde dehydrogenase / alcohol dehydrogenase [EC:1.2.1.10 1.1.1.1]	25-36 months	0.2128	N.S.

K04073: mhpF; acetaldehyde dehydrogenase [EC:1.2.1.10]	N.S.	N.S.	N.S.
K07246: ttuC, dmlA; tartrate dehydrogenase/decarboxylase / D-malate dehydrogenase [EC:1.1.1.93 4.1.1.73 1.1.1.83]	N.S.	N.S.	N.S.
K07250: gabT; 4-aminobutyrate aminotransferase / (S)-3-amino-2-methylpropionate transaminase / 5-aminovalerate transaminase [EC:2.6.1.19 2.6.1.22 2.6.1.48]	N.S.	N.S.	N.S.
K08318: yihU; 4-hydroxybutyrate dehydrogenase / sulfolactaldehyde 3-reductase [EC:1.1.1.61 1.1.1.373]	N.S.	N.S.	N.S.
K08324: sad; succinate-semialdehyde dehydrogenase [EC:1.2.1.16 1.2.1.24]	N.S.	N.S.	N.S.
K11258: ilvM; acetolactate synthase II small subunit [EC:2.2.1.6]	N.S.	N.S.	N.S.
K14534: abfD; 4-hydroxybutyryl-CoA dehydratase / vinylacetyl-CoA-Delta-isomerase [EC:4.2.1.120 5.3.3.3]	13-24 months	0.1001	0.0298
K17829: ccrA; crotonyl-CoA reductase [EC:1.3.1.86]	N.S.	N.S.	N.S.
K17865: croR; 3-hydroxybutyryl-CoA dehydratase [EC:4.2.1.55]	N.S.	N.S.	N.S.
K18118: aarC, cat1; succinyl-CoA:acetate CoA-transferase [EC:2.8.3.18]	N.S.	N.S.	N.S.
K18120: 4hbD, abfH; 4-hydroxybutyrate dehydrogenase [EC:1.1.1.61]	N.S.	N.S.	N.S.
K18122: cat2, abfT; 4-hydroxybutyrate CoA-transferase [EC:2.8.3.-]	N.S.	N.S.	N.S.

*N.S., Not significant

Table S10. Comparison of gene families involved in propionate metabolism among phenotypic groups at each age. Significantly different gene families were selected using the Dunn's post-hoc test and MaAsLin2 analysis. *P*-value was adjusted using the Benjamini–Hochberg method to calculate the *q* value (false discovery rate adjusted *p*-value). N: Non-AD, M: Mild AD, MS: Moderate to Severe AD.

Gene family	6 months					
	Kruskal-Wallis test		Adjusted *P* value (Dunn's Post-hoc test)			Enrichment
	R-squared	*P* value	Non-AD vs. Mild	Non-AD vs. Moderate to Severe	Mild vs. Moderate to Severe	
K00005: gldA; glycerol dehydrogenase [EC:1.1.1.6]	4.6003	0.1002	0.0969	0.0952	0.3211	MS<M<N
K00016: ldh; L-lactate dehydrogenase [EC:1.1.1.27]	1.8367	0.3992	0.2199	0.2856	0.2635	M<N<MS
K00048: fucO; lactaldehyde reductase [EC:1.1.1.77]	1.5149	0.4689	0.3305	0.2332	0.3318	MS<N<M
K00086: dhaT; 1 3-propanediol dehydrogenase [EC:1.1.1.202]	2.8061	0.2458	0.2232	0.1598	0.1431	MS=M=N
K00140: mmsA, iolA; malonate-semialdehyde dehydrogenase (acetylating) / methylmalonate-semialdehyde dehydrogenase [EC:1.2.1.18 1.2.1.27]	0.8841	0.6427	0.4030	0.5974	0.3945	MS=M=N
K00166: bkdA1; 2-oxoisovalerate dehydrogenase E1 component alpha subunit [EC:1.2.4.4]	2.3086	0.3153	0.1833	0.2600	0.2211	MS=N<M
K00167: bkdA2; 2-oxoisovalerate dehydrogenase E1 component beta subunit [EC:1.2.4.4]	2.1058	0.3489	0.3203	0.2522	0.2748	MS=M=N
K00169: porA; pyruvate ferredoxin oxidoreductase alpha subunit [EC:1.2.7.1]	2.0440	0.3599	0.2372	0.3205	0.3736	MS=M<N
K00170: porB; pyruvate ferredoxin oxidoreductase beta subunit [EC:1.2.7.1]	2.7399	0.2541	0.1745	0.2718	0.3441	MS=M<N
K00171: porD; pyruvate ferredoxin oxidoreductase delta subunit [EC:1.2.7.1]	0.3109	0.8560	0.8674	0.4266	0.5949	MS=M=N
K00172: porC porG; pyruvate ferredoxin oxidoreductase gamma subunit [EC:1.2.7.1]	1.2170	0.5442	0.2753	0.3516	0.4616	MS=M=N
K00248: bcd; butyryl-CoA dehydrogenase [EC:1.3.8.1]	4.0284	0.1334	0.2839	0.0672	0.1410	MS=M<N
K00382: lpd, pdhD; dihydrolipoamide dehydrogenase [EC:1.8.1.4]	2.9130	0.2331	0.1103	0.3100	0.1861	M<N<MS
K00625: pta; phosphate acetyltransferase [EC:2.3.1.8]	1.5008	0.4722	0.3047	0.3409	0.3796	MS<M<N
K00626: atoB; acetyl-CoA C-acetyltransferase [EC:2.3.1.9]	0.2686	0.8743	0.4422	0.4932	0.9280	MS<M<N
K00656: pflD; formate C-acetyltransferase [EC:2.3.1.54]	0.8864	0.6420	0.4026	0.5964	0.3943	MS<M<N
K00823: puuE; 4-aminobutyrate aminotransferase [EC:2.6.1.19]	0.2169	0.8972	0.5726	0.3953	0.9668	N<MS<M
K00925: ackA; acetate kinase [EC:2.7.2.1]	1.5105	0.4699	0.2821	0.2757	0.3484	M<N<MS
K00932: tdcD; propionate kinase [EC:2.7.2.15]	0.3250	0.8500	0.9229	0.4481	0.4645	MS<N<M
K01026: pct; propionate CoA-transferase [EC:2.8.3.1]	0.3557	0.8371	0.8279	0.4227	0.5829	MS=M=N
K01034: atoD; acetate CoA/acetoacetate CoA-transferase alpha subunit [EC:2.8.3.8 2.8.3.9]	1.0380	0.5951	0.2834	0.3953	0.5491	N<MS<M
K01035: atoA; acetate CoA/acetoacetate CoA-transferase beta subunit [EC:2.8.3.8 2.8.3.9]	1.4405	0.4866	0.3775	0.4809	0.2700	N<MS<M
K01659: prpC; 2-methylcitrate synthase [EC:2.3.3.5]	6.3216	0.0424	0.0749	0.0789	0.0180	MS<N<M
K01682: acnB; aconitate hydratase 2 / 2-methylisocitrate dehydratase [EC:4.2.1.3 4.2.1.99]	0.4130	0.8134	0.4292	0.4365	0.8062	MS<N<M
K01692: paaF, echA; enoyl-CoA hydratase [EC:4.2.1.17]	3.0747	0.2149	0.1193	0.3165	0.2686	MS=M<N
K01699: pduC; propanediol dehydratase large subunit [EC:4.2.1.28]	1.8992	0.3869	0.2993	0.2017	0.2550	MS<N<M
K01720: prpD; 2-methylcitrate dehydratase [EC:4.2.1.79]	0.1356	0.9344	0.4277	0.5949	1.0000	M<N<MS
K01734: mgsA; methylglyoxal synthase [EC:4.2.3.3]	4.7852	0.0914	0.1152	0.0733	0.2634	MS<M<N
K01782: fadJ; 3-hydroxyacyl-CoA dehydrogenase / enoyl-CoA hydratase / 3-hydroxybutyryl-CoA epimerase [EC:1.1.1.35 4.2.1.17 5.1.2.3]	6.9865	0.0304	0.2001	0.0125	0.0723	MS<M<N
K01825: fadB; 3-hydroxyacyl-CoA dehydrogenase / enoyl-CoA hydratase / 3-hydroxybutyryl-CoA epimerase / enoyl-CoA isomerase [EC:1.1.1.35 4.2.1.17 5.1.2.3 5.3.3.8]	3.2093	0.2010	0.1860	0.1576	0.2964	MS=M=N
K01847: MUT; methylmalonyl-CoA mutase [EC:5.4.99.2]	2.0668	0.3558	0.3250	0.2576	0.2755	MS<M<N
K01848: mcmA1; methylmalonyl-CoA mutase N-terminal domain [EC:5.4.99.2]	4.1059	0.1284	0.2298	0.0770	0.0701	MS<N<M
K01849: mcmA2; methylmalonyl-CoA mutase C-terminal domain [EC:5.4.99.2]	4.8687	0.0877	0.3481	0.0343	0.0618	MS<N<M
K01895: acs; acetyl-CoA synthetase [EC:6.2.1.1]	0.1958	0.9067	0.5631	1.0000	0.4644	MS<M<N
K01902: sucD; succinyl-CoA synthetase alpha subunit [EC:6.2.1.5]	1.9872	0.3702	0.2489	0.4580	0.2514	M<N<MS
K01903: sucC; succinyl-CoA synthetase beta subunit [EC:6.2.1.5]	1.5151	0.4688	0.2448	0.3152	0.3278	M<N<MS

K01905: acdA; acetate---CoA ligase (ADP-forming) subunit alpha [EC:6.2.1.13]	0.7097	0.7013	0.7159	0.4013	0.5000	MS=M=N
K01908: prpE; propionyl-CoA synthetase [EC:6.2.1.17]	0.0566	0.9721	0.6458	0.4561	1.0000	MS=M=N
K01961: accC; acetyl-CoA carboxylase biotin carboxylase subunit [EC:6.4.1.2 6.3.4.14]	0.7725	0.6796	0.4738	0.6099	0.3289	MS<N<M
K01962: accA; acetyl-CoA carboxylase carboxyl transferase subunit alpha [EC:6.4.1.2 2.1.3.15]	0.4854	0.7845	0.4693	0.3613	0.7433	M<MS<N
K01963: accD; acetyl-CoA carboxylase carboxyl transferase subunit beta [EC:6.4.1.2 2.1.3.15]	0.0093	0.9954	0.4930	0.6980	1.0000	M<N<MS
K01966: pccB; propionyl-CoA carboxylase beta chain [EC:6.4.1.3 2.1.3.15]	0.8831	0.6430	0.3440	0.5329	0.4622	MS=M=N
K02160: accB, bccP; acetyl-CoA carboxylase biotin carboxyl carrier protein	3.9561	0.1383	0.0741	0.3160	0.2133	M<MS<N
K03417: prpB; methylisocitrate lyase [EC:4.1.3.30]	0.1146	0.9443	0.6410	1.0000	0.4436	MS<N<M
K05606: epi; methylmalonyl-CoA/ethylmalonyl-CoA epimerase [EC:5.1.99.1]	5.7247	0.0571	0.1593	0.0489	0.0267	MS<N<M
K07250: gabT; 4-aminobutyrate aminotransferase / (S)-3-amino-2-methylpropionate transaminase / 5-aminovalerate transaminase [EC:2.6.1.19 2.6.1.22 2.6.1.48]	2.0117	0.3657	0.3079	0.1869	0.2474	MS<N<M
K08325: yqhD; NADP-dependent alcohol dehydrogenase [EC:1.1.-.-]	2.9231	0.2319	0.4080	0.1724	0.0902	MS<N<M
K09788: prpF; 2-methylaconitate isomerase [EC:5.3.3.-]	2.1721	0.3375	0.4836	0.2273	0.1666	MS<M<N
K11263: bccA, pccA; acetyl-CoA/propionyl-CoA carboxylase biotin carboxylase biotin carboxyl carrier protein [EC:6.4.1.2 6.4.1.3 6.3.4.14]	1.9471	0.3777	0.2561	0.2304	0.2463	N<M<MS
K11264: scpB, mmcD; methylmalonyl-CoA decarboxylase [EC:4.1.1.-]	0.1454	0.9299	0.4891	1.0000	0.5529	M<N<MS
K11381: bkdA; 2-oxoisovalerate dehydrogenase E1 component [EC:1.2.4.4]	0.7097	0.7013	0.7159	0.4013	0.5000	MS=M=N
K13788: pta; phosphate acetyltransferase [EC:2.3.1.8]	1.0027	0.6057	0.4038	0.2859	0.5130	N<MS<M
K13919: pduD; propanediol dehydratase medium subunit [EC:4.2.1.28]	2.5147	0.2844	0.3021	0.1621	0.4576	MS=M<N
K13920: pduE; propanediol dehydratase small subunit [EC:4.2.1.28]	0.3587	0.8358	0.8249	0.4525	0.5440	MS<M<N
K13921: pduQ; 1-propanol dehydrogenase	3.7852	0.1507	0.1514	0.1207	0.2898	MS=M<N
K13922: pduP; propionaldehyde dehydrogenase [EC:1.2.1.87]	0.6343	0.7282	0.3960	0.7910	0.4717	MS=M=N
K13923: pduL; phosphate propanoyltransferase [EC:2.3.1.222]	0.2598	0.8782	0.9663	0.4644	0.5005	MS<N<M
K15024: putative phosphotransacetylase [EC:2.3.1.8]	3.4563	0.1776	0.0989	0.2298	0.3376	MS=M=N
K18369: adh2; alcohol dehydrogenase [EC:1.1.1.-]	0.0933	0.9544	0.6569	1.0000	0.4453	N<MS<M
K18471: ydjG; methylglyoxal reductase [EC:1.1.1.-]	0.0892	0.9564	0.4700	0.5962	1.0000	M<N<MS

Gene family	7-12 months					
	Kruskal-Wallis test		Adjusted *P* value (Dunn's Post-hoc test)			Enrichment
	R-squared	*P* value	Non-AD vs. Mild	Non-AD vs. Moderate to Severe	Mild vs. Moderate to Severe	
K00005: gldA; glycerol dehydrogenase [EC:1.1.1.6]	0.6300	0.7298	0.4111	0.5460	0.6415	N<MS<M
K00016: ldh; L-lactate dehydrogenase [EC:1.1.1.27]	6.1539	0.0461	0.1725	0.2814	0.0220	MS<N<M
K00048: fucO; lactaldehyde reductase [EC:1.1.1.77]	7.3083	0.0259	0.3392	0.0262	0.0245	MS<M<N
K00086: dhaT; 1 3-propanediol dehydrogenase [EC:1.1.1.202]	0.0847	0.9585	0.6379	1.0000	0.4402	N=M=MS
K00140: mmsA, iolA; malonate-semialdehyde dehydrogenase (acetylating) / methylmalonate-semialdehyde dehydrogenase [EC:1.2.1.18 1.2.1.27]	5.5350	0.0628	0.3857	0.1302	0.0317	N=M=MS
K00166: bkdA1; 2-oxoisovalerate dehydrogenase E1 component alpha subunit [EC:1.2.4.4]	0.4883	0.7834	0.7567	0.3620	0.5125	N=M=MS
K00167: bkdA2; 2-oxoisovalerate dehydrogenase E1 component beta subunit [EC:1.2.4.4]	5.3488	0.0689	0.0346	0.1245	0.1290	N=M=MS
K00169: porA; pyruvate ferredoxin oxidoreductase alpha subunit [EC:1.2.7.1]	4.9861	0.0827	0.2004	0.3126	0.0428	N<MS<M
K00170: porB; pyruvate ferredoxin oxidoreductase beta subunit [EC:1.2.7.1]	3.7589	0.1527	0.3228	0.2699	0.0804	MS<N<M
K00171: porD; pyruvate ferredoxin oxidoreductase delta subunit [EC:1.2.7.1]	1.3945	0.4980	0.3602	0.4654	0.3566	N=M=MS
K00172: porC porG; pyruvate ferredoxin oxidoreductase gamma subunit [EC:1.2.7.1]	3.2819	0.1938	0.2648	0.1610	0.1186	MS<M<N
K00248: bcd; butyryl-CoA dehydrogenase [EC:1.3.8.1]	8.1677	0.0168	0.3023	0.0975	0.0070	MS<N<M
K00382: lpd, pdhD; dihydrolipoamide dehydrogenase [EC:1.8.1.4]	5.0997	0.0781	0.1110	0.4594	0.0531	MS<N<M
K00625: pta; phosphate acetyltransferase [EC:2.3.1.8]	2.4355	0.2959	0.3263	0.3743	0.1782	MS<M<N

K00626: atoB; acetyl-CoA C-acetyltransferase [EC:2.3.1.9]	4.5623	0.1022	0.1472	0.0636	0.1142	MS<M<N
K00656: pflD; formate C-acetyltransferase [EC:2.3.1.54]	10.3860	0.0056	0.4603	0.0185	0.0038	MS<N<M
K00823: puuE; 4-aminobutyrate aminotransferase [EC:2.6.1.19]	3.0529	0.2173	0.1006	0.1302	0.3530	M<MS<N
K00925: ackA; acetate kinase [EC:2.7.2.1]	7.2531	0.0266	0.1888	0.2150	0.0106	MS<M<N
K00932: tdcD; propionate kinase [EC:2.7.2.15]	2.2818	0.3195	0.2108	0.1424	0.4387	M<MS<N
K01026: pct; propionate CoA-transferase [EC:2.8.3.1]	1.2733	0.5291	0.3732	0.4011	0.2612	N=M=MS
K01034: atoD; acetate CoA/acetoacetate CoA-transferase alpha subunit [EC:2.8.3.8 2.8.3.9]	0.5919	0.7438	0.5161	0.4417	0.6800	M<N<MS
K01035: atoA; acetate CoA/acetoacetate CoA-transferase beta subunit [EC:2.8.3.8 2.8.3.9]	0.6280	0.7305	0.4729	0.4721	0.6617	N<M<MS
K01659: prpC; 2-methylcitrate synthase [EC:2.3.3.5]	3.7254	0.1553	0.1465	0.4962	0.1155	M<MS<N
K01682: acnB; aconitate hydratase 2 / 2-methylisocitrate dehydratase [EC:4.2.1.3 4.2.1.99]	0.2292	0.8917	0.4890	0.5719	0.9662	MS<M<N
K01692: paaF, echA; enoyl-CoA hydratase [EC:4.2.1.17]	5.0314	0.0808	0.0373	0.0749	0.2337	MS=M<N
K01699: pduC; propanediol dehydratase large subunit [EC:4.2.1.28]	0.6198	0.7335	0.3853	0.6543	0.4170	N<MS<M
K01720: prpD; 2-methylcitrate dehydratase [EC:4.2.1.79]	3.8469	0.1461	0.3174	0.2687	0.0764	M<N<MS
K01734: mgsA; methylglyoxal synthase [EC:4.2.3.3]	0.4128	0.8135	0.4595	0.5431	0.7893	M<N<MS
K01782: fadJ; 3-hydroxyacyl-CoA dehydrogenase / enoyl-CoA hydratase / 3-hydroxybutyryl-CoA epimerase [EC:1.1.1.35 4.2.1.17 5.1.2.3]	4.4828	0.1063	0.0566	0.1557	0.1481	M<MS<N
K01825: fadB; 3-hydroxyacyl-CoA dehydrogenase / enoyl-CoA hydratase / 3-hydroxybutyryl-CoA epimerase / enoyl-CoA isomerase [EC:1.1.1.35 4.2.1.17 5.1.2.3 5.3.3.8]	4.6820	0.0962	0.0614	0.1515	0.1396	N=M=MS
K01847: MUT; methylmalonyl-CoA mutase [EC:5.4.99.2]	0.7693	0.6807	0.4679	0.4488	0.5060	MS<N<M
K01848: mcmA1; methylmalonyl-CoA mutase N-terminal domain [EC:5.4.99.2]	0.8288	0.6607	0.4634	0.4402	0.5618	M<N<MS
K01849: mcmA2; methylmalonyl-CoA mutase C-terminal domain [EC:5.4.99.2]	2.7427	0.2538	0.1505	0.4071	0.2408	N<MS<M
K01895: acs; acetyl-CoA synthetase [EC:6.2.1.1]	1.9811	0.3714	0.2710	0.4804	0.2874	N<MS<M
K01902: sucD; succinyl-CoA synthetase alpha subunit [EC:6.2.1.5]	1.0923	0.5792	0.3180	0.4459	0.5585	MS<N<M
K01903: sucC; succinyl-CoA synthetase beta subunit [EC:6.2.1.5]	5.3376	0.0693	0.0880	0.4962	0.0525	MS<N<M
K01905: acdA; acetate---CoA ligase (ADP-forming) subunit alpha [EC:6.2.1.13]	4.1148	0.1278	0.0464	0.0837	0.4373	N=M=MS
K01908: prpE; propionyl-CoA synthetase [EC:6.2.1.17]	0.3709	0.8307	0.4277	0.8553	0.4885	MS=M<N
K01961: accC; acetyl-CoA carboxylase biotin carboxylase subunit [EC:6.4.1.2 6.3.4.14]	1.0708	0.5854	0.4545	0.3659	0.3370	N<M<MS
K01962: accA; acetyl-CoA carboxylase carboxyl transferase subunit alpha [EC:6.4.1.2 2.1.3.15]	2.0975	0.3504	0.4313	0.2042	0.2860	MS<M<N
K01963: accD; acetyl-CoA carboxylase carboxyl transferase subunit beta [EC:6.4.1.2 2.1.3.15]	2.2492	0.3248	0.4492	0.2706	0.2175	MS<M<N
K01966: pccB; propionyl-CoA carboxylase beta chain [EC:6.4.1.3 2.1.3.15]	1.7936	0.4079	0.2557	0.2712	0.2774	N=M<MS
K02160: accB, bccP; acetyl-CoA carboxylase biotin carboxyl carrier protein	1.9423	0.3786	0.4526	0.2291	0.3014	N<MS<M
K03417: prpB; methylisocitrate lyase [EC:4.1.3.30]	1.6385	0.4408	0.4378	0.3866	0.2296	M<N<MS
K05606: epi; methylmalonyl-CoA/ethylmalonyl-CoA epimerase [EC:5.1.99.1]	0.2792	0.8697	0.4665	0.5789	0.9032	MS<N<M
K07250: gabT; 4-aminobutyrate aminotransferase / (S)-3-amino-2-methylpropionate transaminase / 5-aminovalerate transaminase [EC:2.6.1.19 2.6.1.22 2.6.1.48]	3.0193	0.2210	0.3403	0.1118	0.2123	N<M<MS
K08325: yqhD; NADP-dependent alcohol dehydrogenase [EC:1.1.-.-]	1.1316	0.5679	0.4397	0.3788	0.3129	N<M<MS
K09788: prpF; 2-methylaconitate isomerase [EC:5.3.3.-]	6.9120	0.0316	0.0167	0.0147	0.4365	N<M<MS
K11263: bccA, pccA; acetyl-CoA/propionyl-CoA carboxylase biotin carboxylase biotin carboxyl carrier protein [EC:6.4.1.2 6.4.1.3 6.3.4.14]	5.0858	0.0786	0.1868	0.3238	0.0413	MS<N<M
K11264: scpB, mmcD; methylmalonyl-CoA decarboxylase [EC:4.1.1.-]	9.1029	0.0106	0.0044	0.0084	0.3200	MS<M<N
K11381: bkdA; 2-oxoisovalerate dehydrogenase E1 component [EC:1.2.4.4]	0.9840	0.6114	0.2745	0.5188	0.4602	N=M=MS
K13788: pta; phosphate acetyltransferase [EC:2.3.1.8]	3.8537	0.1456	0.2848	0.2974	0.0746	MS<M<N
K13919: pduD; propanediol dehydratase medium subunit [EC:4.2.1.28]	0.5262	0.7687	0.3456	0.7233	0.4898	N=M=MS
K13920: pduE; propanediol dehydratase small subunit [EC:4.2.1.28]	0.0251	0.9875	0.7143	0.4781	1.0000	MS<M<N
K13921: pduQ; 1-propanol dehydrogenase	0.3053	0.8584	0.8714	0.5218	0.4214	N=M=MS
K13922: pduP; propionaldehyde dehydrogenase [EC:1.2.1.87]	2.1073	0.3487	0.2215	0.1911	0.3589	N=M=MS
K13923: pduL; phosphate propanoyltransferase [EC:2.3.1.222]	0.6541	0.7211	0.4522	0.4865	0.6778	M<MS<N
K15024: putative phosphotransacetylase [EC:2.3.1.8]	0.7273	0.6951	0.4191	0.5125	0.5930	N=M=MS

K18369: adh2; alcohol dehydrogenase [EC:1.1.1.-]	1.5516	0.4603	0.4438	0.2607	0.3885	MS<M<N
K18471: ydjG; methylglyoxal reductase [EC:1.1.1.-]	5.4522	0.0655	0.0702	0.0294	0.1566	MS<M<N

Gene family	13-24 months					
	Kruskal-Wallis test		Adjusted *P* value (Dunn's Post-hoc test)			Enrichment
	R-squared	*P* value	Non-AD vs. Mild	Non-AD vs. Moderate to Severe	Mild vs. Moderate to Severe	
K00005: gldA; glycerol dehydrogenase [EC:1.1.1.6]	1.5690	0.4564	0.3277	0.3330	0.2752	N<MS<M
K00016: ldh; L-lactate dehydrogenase [EC:1.1.1.27]	3.9718	0.1373	0.1794	0.0814	0.1549	N<M<MS
K00048: fucO; lactaldehyde reductase [EC:1.1.1.77]	0.3856	0.8246	0.8025	0.4807	0.3939	N<MS<M
K00086: dhaT; 1 3-propanediol dehydrogenase [EC:1.1.1.202]	3.1514	0.2069	0.1396	0.3865	0.1613	N=M=MS
K00140: mmsA, iolA; malonate-semialdehyde dehydrogenase (acetylating) / methylmalonate-semialdehyde dehydrogenase [EC:1.2.1.18 1.2.1.27]	2.9358	0.2304	0.3509	0.1341	0.2492	N=M=MS
K00166: bkdA1; 2-oxoisovalerate dehydrogenase E1 component alpha subunit [EC:1.2.4.4]	2.9240	0.2318	0.1469	0.3791	0.1864	N=M=MS
K00167: bkdA2; 2-oxoisovalerate dehydrogenase E1 component beta subunit [EC:1.2.4.4]	1.8886	0.3889	0.1543	0.2791	0.4981	N=M=MS
K00169: porA; pyruvate ferredoxin oxidoreductase alpha subunit [EC:1.2.7.1]	2.8178	0.2444	0.1858	0.4448	0.1733	M<N<MS
K00170: porB; pyruvate ferredoxin oxidoreductase beta subunit [EC:1.2.7.1]	2.3049	0.3159	0.3300	0.4280	0.1970	MS<N<M
K00171: porD; pyruvate ferredoxin oxidoreductase delta subunit [EC:1.2.7.1]	0.9501	0.6219	0.4357	0.4710	0.5088	MS=M<N
K00172: porC porG; pyruvate ferredoxin oxidoreductase gamma subunit [EC:1.2.7.1]	3.8220	0.1479	0.1157	0.1963	0.1076	M<MS<N
K00248: bcd; butyryl-CoA dehydrogenase [EC:1.3.8.1]	2.5381	0.2811	0.1710	0.2014	0.2100	M<MS<N
K00382: lpd, pdhD; dihydrolipoamide dehydrogenase [EC:1.8.1.4]	0.3298	0.8480	0.9374	0.4237	0.4791	M<MS<N
K00625: pta; phosphate acetyltransferase [EC:2.3.1.8]	3.3342	0.1888	0.1856	0.2793	0.0986	M<MS<N
K00626: atoB; acetyl-CoA C-acetyltransferase [EC:2.3.1.9]	1.4829	0.4764	0.4043	0.4324	0.3496	M<N<MS
K00656: pflD; formate C-acetyltransferase [EC:2.3.1.54]	2.9066	0.2338	0.2359	0.1894	0.1914	M<N<MS
K00823: puuE; 4-aminobutyrate aminotransferase [EC:2.6.1.19]	2.4398	0.2953	0.2098	0.2030	0.2203	N<MS<M
K00925: ackA; acetate kinase [EC:2.7.2.1]	1.8560	0.3954	0.4298	0.3678	0.2598	M<MS<N
K00932: tdcD; propionate kinase [EC:2.7.2.15]	0.4738	0.7891	0.4508	0.7372	0.4184	N=M=MS
K01026: pct; propionate CoA-transferase [EC:2.8.3.1]	0.6958	0.7062	0.6067	0.3781	0.3816	MS<N<M
K01034: atoD; acetate CoA/acetoacetate CoA-transferase alpha subunit [EC:2.8.3.8 2.8.3.9]	1.5933	0.4508	0.4389	0.3797	0.3381	M<N<MS
K01035: atoA; acetate CoA/acetoacetate CoA-transferase beta subunit [EC:2.8.3.8 2.8.3.9]	1.7696	0.4128	0.3689	0.4392	0.2826	N<M<MS
K01659: prpC; 2-methylcitrate synthase [EC:2.3.3.5]	2.3860	0.3033	0.1840	0.1842	0.3161	N<MS<M
K01682: acnB; aconitate hydratase 2 / 2-methylisocitrate dehydratase [EC:4.2.1.3 4.2.1.99]	2.1906	0.3344	0.1404	0.2211	0.4608	N=M=MS
K01692: paaF, echA; enoyl-CoA hydratase [EC:4.2.1.17]	0.4496	0.7987	0.5174	0.4895	0.7713	N=M=MS
K01699: pduC; propanediol dehydratase large subunit [EC:4.2.1.28]	2.3347	0.3112	0.2031	0.1921	0.2944	N<M<MS
K01720: prpD; 2-methylcitrate dehydratase [EC:4.2.1.79]	4.0366	0.1329	0.0474	0.0809	0.4890	N<MS<M
K01734: mgsA; methylglyoxal synthase [EC:4.2.3.3]	2.2759	0.3205	0.2292	0.2080	0.2362	M<MS<N
K01782: fadJ; 3-hydroxyacyl-CoA dehydrogenase / enoyl-CoA hydratase / 3-hydroxybutyryl-CoA epimerase [EC:1.1.1.35 4.2.1.17 5.1.2.3]	2.2434	0.3257	0.2676	0.2620	0.1926	MS=N<M
K01825: fadB; 3-hydroxyacyl-CoA dehydrogenase / enoyl-CoA hydratase / 3-hydroxybutyryl-CoA epimerase / enoyl-CoA isomerase [EC:1.1.1.35 4.2.1.17 5.1.2.3 5.3.3.8]	3.0256	0.2203	0.1497	0.1759	0.1831	N=M=MS
K01847: MUT; methylmalonyl-CoA mutase [EC:5.4.99.2]	10.6078	0.0050	0.0461	0.0033	0.0430	MS<M<N
K01848: mcmA1; methylmalonyl-CoA mutase N-terminal domain [EC:5.4.99.2]	0.6608	0.7186	0.3363	0.6577	0.4887	N<M<MS
K01849: mcmA2; methylmalonyl-CoA mutase C-terminal domain [EC:5.4.99.2]	0.3335	0.8464	0.8712	0.3727	0.5321	N<M<MS
K01895: acs; acetyl-CoA synthetase [EC:6.2.1.1]	0.3872	0.8240	0.4881	0.8026	0.4151	MS<M<N
K01902: sucD; succinyl-CoA synthetase alpha subunit [EC:6.2.1.5]	2.9042	0.2341	0.3312	0.3670	0.1376	M<N<MS
K01903: sucC; succinyl-CoA synthetase beta subunit [EC:6.2.1.5]	0.9356	0.6264	0.4488	0.4628	0.5270	M<MS<N
K01905: acdA; acetate---CoA ligase (ADP-forming) subunit alpha [EC:6.2.1.13]	1.8326	0.4000	0.2819	0.1659	0.4093	N=M=MS

K01908: prpE; propionyl-CoA synthetase [EC:6.2.1.17]	2.3717	0.3055	0.1902	0.1412	0.3385	N=M=MS
K01961: accC; acetyl-CoA carboxylase biotin carboxylase subunit [EC:6.4.1.2 6.3.4.14]	0.5028	0.7777	0.4862	0.5078	0.7593	N<M<MS
K01962: accA; acetyl-CoA carboxylase carboxyl transferase subunit alpha [EC:6.4.1.2 2.1.3.15]	6.2976	0.0429	0.0970	0.4721	0.0223	M<N<MS
K01963: accD; acetyl-CoA carboxylase carboxyl transferase subunit beta [EC:6.4.1.2 2.1.3.15]	5.7326	0.0569	0.1082	0.4799	0.0306	M<N<MS
K01966: pccB; propionyl-CoA carboxylase beta chain [EC:6.4.1.3 2.1.3.15]	2.3334	0.3114	0.1532	0.1921	0.3890	MS<M<N
K02160: accB, bccP; acetyl-CoA carboxylase biotin carboxyl carrier protein	6.5491	0.0378	0.4426	0.1026	0.0242	M<N<MS
K03417: prpB; methylisocitrate lyase [EC:4.1.3.30]	2.4761	0.2900	0.2007	0.1066	0.4401	N<MS<M
K05606: epi; methylmalonyl-CoA/ethylmalonyl-CoA epimerase [EC:5.1.99.1]	2.7528	0.2525	0.2948	0.2408	0.1639	MS<M<N
K07250: gabT; 4-aminobutyrate aminotransferase / (S)-3-amino-2-methylpropionate transaminase / 5-aminovalerate transaminase [EC:2.6.1.19 2.6.1.22 2.6.1.48]	1.8292	0.4007	0.2680	0.3249	0.2915	N<M<MS
K08325: yqhD; NADP-dependent alcohol dehydrogenase [EC:1.1.-.-]	0.5442	0.7618	0.6945	0.4021	0.4128	M<N<MS
K09788: prpF; 2-methylaconitate isomerase [EC:5.3.3.-]	0.1141	0.9446	1.0000	0.5813	0.4616	N=M=MS
K11263: bccA, pccA; acetyl-CoA/propionyl-CoA carboxylase biotin carboxylase biotin carboxyl carrier protein [EC:6.4.1.2 6.4.1.3 6.3.4.14]	3.1899	0.2029	0.2199	0.1272	0.1804	N<M<MS
K11264: scpB, mmcD; methylmalonyl-CoA decarboxylase [EC:4.1.1.-]	5.4568	0.0653	0.0608	0.1860	0.0495	MS=N<M
K11381: bkdA; 2-oxoisovalerate dehydrogenase E1 component [EC:1.2.4.4]	0.5671	0.7531	0.3434	0.7141	0.5025	N=M=MS
K13788: pta; phosphate acetyltransferase [EC:2.3.1.8]	3.0874	0.2136	0.2831	0.2054	0.1449	N<M<MS
K13919: pduD; propanediol dehydratase medium subunit [EC:4.2.1.28]	0.8372	0.6580	0.5449	0.3897	0.3254	N=M=MS
K13920: pduE; propanediol dehydratase small subunit [EC:4.2.1.28]	4.3860	0.1116	0.0662	0.1138	0.1392	N=M=MS
K13921: pduQ; 1-propanol dehydrogenase	5.4846	0.0644	0.0382	0.0965	0.0955	N=M=MS
K13922: pduP; propionaldehyde dehydrogenase [EC:1.2.1.87]	0.0016	0.9992	1.0000	0.7296	0.4956	N=M=MS
K13923: pduL; phosphate propanoyltransferase [EC:2.3.1.222]	3.7934	0.1501	0.0819	0.0675	0.3201	N=M=MS
K15024: putative phosphotransacetylase [EC:2.3.1.8]	0.8426	0.6562	0.3119	0.5779	0.4520	N=M=MS
K18369: adh2; alcohol dehydrogenase [EC:1.1.1.-]	0.5596	0.7559	0.3928	0.6870	0.4566	M<N<MS
K18471: ydjG; methylglyoxal reductase [EC:1.1.1.-]	5.1935	0.0745	0.0485	0.2728	0.0761	M<MS<N

Gene family	25-36 months					
	Kruskal-Wallis test		Adjusted *P* value (Dunn's Post-hoc test)			Enrichment
	R-squared	*P* value	Non-AD vs. Mild	Non-AD vs. Moderate to Severe	Mild vs. Moderate to Severe	
K00005: gldA; glycerol dehydrogenase [EC:1.1.1.6]	0.0547	0.9730	0.4801	1.0000	0.6816	N<MS<M
K00016: ldh; L-lactate dehydrogenase [EC:1.1.1.27]	2.0658	0.3560	0.2026	0.2717	0.2262	MS<M<N
K00048: fucO; lactaldehyde reductase [EC:1.1.1.77]	0.2004	0.9047	0.4448	0.5304	1.0000	M<N<MS
K00086: dhaT; 1 3-propanediol dehydrogenase [EC:1.1.1.202]	1.5432	0.4623	0.3068	0.4010	0.5000	N=M=MS
K00140: mmsA, iolA; malonate-semialdehyde dehydrogenase (acetylating) / methylmalonate-semialdehyde dehydrogenase [EC:1.2.1.18 1.2.1.27]	0.6749	0.7136	0.7610	0.3920	0.4313	N=M=MS
K00166: bkdA1; 2-oxoisovalerate dehydrogenase E1 component alpha subunit [EC:1.2.4.4]	2.2231	0.3291	0.4984	0.2248	0.2403	N=M=MS
K00167: bkdA2; 2-oxoisovalerate dehydrogenase E1 component beta subunit [EC:1.2.4.4]	0.3454	0.8414	0.5148	0.3786	0.8373	N=M=MS
K00169: porA; pyruvate ferredoxin oxidoreductase alpha subunit [EC:1.2.7.1]	8.0314	0.0180	0.4363	0.0083	0.0588	MS<N<M
K00170: porB; pyruvate ferredoxin oxidoreductase beta subunit [EC:1.2.7.1]	12.5514	0.0019	0.0131	0.0268	0.0006	MS<N<M
K00171: porD; pyruvate ferredoxin oxidoreductase delta subunit [EC:1.2.7.1]	5.8089	0.0548	0.3515	0.0247	0.1461	MS<M<N
K00172: porC porG; pyruvate ferredoxin oxidoreductase gamma subunit [EC:1.2.7.1]	5.0826	0.0788	0.2447	0.0365	0.2723	MS<N<M
K00248: bcd; butyryl-CoA dehydrogenase [EC:1.3.8.1]	0.0064	0.9968	0.7205	0.4806	1.0000	M<MS<N
K00382: lpd, pdhD; dihydrolipoamide dehydrogenase [EC:1.8.1.4]	2.3186	0.3137	0.1162	0.4364	0.2316	M<N<MS
K00625: pta; phosphate acetyltransferase [EC:2.3.1.8]	0.4481	0.7993	0.3522	0.4973	0.7736	M<N<MS
K00626: atoB; acetyl-CoA C-acetyltransferase [EC:2.3.1.9]	0.6641	0.7174	0.4708	0.6328	0.4710	M<N<MS
K00656: pflD; formate C-acetyltransferase [EC:2.3.1.54]	5.0451	0.0803	0.0852	0.0759	0.2634	N<MS<M

K00823: puuE; 4-aminobutyrate aminotransferase [EC:2.6.1.19]	3.6971	0.1575	0.1319	0.1654	0.0912	MS<N<M
K00925: ackA; acetate kinase [EC:2.7.2.1]	0.0405	0.9799	1.0000	0.6570	0.4828	N<MS<M
K00932: tdcD; propionate kinase [EC:2.7.2.15]	1.1674	0.5578	0.4985	0.3582	0.3418	N=M=MS
K01026: pct; propionate CoA-transferase [EC:2.8.3.1]	4.1393	0.1262	0.1441	0.1202	0.0663	MS<N<M
K01034: atoD; acetate CoA/acetoacetate CoA-transferase alpha subunit [EC:2.8.3.8 2.8.3.9]	0.5670	0.7532	0.3672	0.4555	0.7117	MS<N<M
K01035: atoA; acetate CoA/acetoacetate CoA-transferase beta subunit [EC:2.8.3.8 2.8.3.9]	5.1569	0.0759	0.3534	0.0356	0.1705	MS<M<N
K01659: prpC; 2-methylcitrate synthase [EC:2.3.3.5]	0.2031	0.9034	0.6126	0.9973	0.4666	M<MS<N
K01682: acnB; aconitate hydratase 2 / 2-methylisocitrate dehydratase [EC:4.2.1.3 4.2.1.99]	2.1050	0.3491	0.2259	0.4293	0.1710	N=M=MS
K01692: paaF, echA; enoyl-CoA hydratase [EC:4.2.1.17]	1.3849	0.5003	0.4134	0.3590	0.3015	N=M=MS
K01699: pduC; propanediol dehydratase large subunit [EC:4.2.1.28]	1.0321	0.5969	0.4850	0.4827	0.3885	N=M=MS
K01720: prpD; 2-methylcitrate dehydratase [EC:4.2.1.79]	0.1904	0.9092	1.0000	0.5499	0.4642	M<MS<N
K01734: mgsA; methylglyoxal synthase [EC:4.2.3.3]	3.7271	0.1551	0.1324	0.1622	0.0901	M<N<MS
K01782: fadJ; 3-hydroxyacyl-CoA dehydrogenase / enoyl-CoA hydratase / 3-hydroxybutyryl-CoA epimerase [EC:1.1.1.35 4.2.1.17 5.1.2.3]	1.1957	0.5500	0.3713	0.4751	0.4769	N=M=MS
K01825: fadB; 3-hydroxyacyl-CoA dehydrogenase / enoyl-CoA hydratase / 3-hydroxybutyryl-CoA epimerase / enoyl-CoA isomerase [EC:1.1.1.35 4.2.1.17 5.1.2.3 5.3.3.8]	0.5438	0.7619	0.3383	0.4731	0.7112	N=M=MS
K01847: MUT; methylmalonyl-CoA mutase [EC:5.4.99.2]	0.9309	0.6278	0.4470	0.5073	0.4443	M<N<MS
K01848: mcmA1; methylmalonyl-CoA mutase N-terminal domain [EC:5.4.99.2]	3.5059	0.1733	0.1871	0.1293	0.1178	M<N<MS
K01849: mcmA2; methylmalonyl-CoA mutase C-terminal domain [EC:5.4.99.2]	8.0588	0.0178	0.4659	0.0098	0.0384	M<N<MS
K01895: acs; acetyl-CoA synthetase [EC:6.2.1.1]	1.7582	0.4152	0.3322	0.2037	0.3710	MS<M<N
K01902: sucD; succinyl-CoA synthetase alpha subunit [EC:6.2.1.5]	0.5446	0.7616	0.4451	0.6925	0.5287	MS<M<N
K01903: sucC; succinyl-CoA synthetase beta subunit [EC:6.2.1.5]	4.8478	0.0886	0.3483	0.0423	0.1880	MS<M<N
K01905: acdA; acetate---CoA ligase (ADP-forming) subunit alpha [EC:6.2.1.13]	3.8014	0.1495	0.0966	0.2127	0.1657	N=M=MS
K01908: prpE; propionyl-CoA synthetase [EC:6.2.1.17]	1.9751	0.3725	0.2128	0.2733	0.2400	N=M=MS
K01961: accC; acetyl-CoA carboxylase biotin carboxylase subunit [EC:6.4.1.2 6.3.4.14]	2.5129	0.2847	0.1707	0.3822	0.1571	M<N<MS
K01962: accA; acetyl-CoA carboxylase carboxyl transferase subunit alpha [EC:6.4.1.2 2.1.3.15]	0.0761	0.9627	0.4970	1.0000	0.6443	N<M<MS
K01963: accD; acetyl-CoA carboxylase carboxyl transferase subunit beta [EC:6.4.1.2 2.1.3.15]	0.1019	0.9503	0.4797	1.0000	0.6479	M<N<MS
K01966: pccB; propionyl-CoA carboxylase beta chain [EC:6.4.1.3 2.1.3.15]	10.5526	0.0051	0.0271	0.0050	0.4667	MS<M<N
K02160: accB, bccP; acetyl-CoA carboxylase biotin carboxyl carrier protein	1.5003	0.4723	0.3210	0.2423	0.3378	MS<N<M
K03417: prpB; methylisocitrate lyase [EC:4.1.3.30]	0.3481	0.8403	0.4232	0.8345	0.6162	MS<M<N
K05606: epi; methylmalonyl-CoA/ethylmalonyl-CoA epimerase [EC:5.1.99.1]	3.5188	0.1722	0.0977	0.2104	0.1875	M<MS<N
K07250: gabT; 4-aminobutyrate aminotransferase / (S)-3-amino-2-methylpropionate transaminase / 5-aminovalerate transaminase [EC:2.6.1.19 2.6.1.22 2.6.1.48]	0.4739	0.7890	0.7809	0.5146	0.3761	M<N<MS
K08325: yqhD; NADP-dependent alcohol dehydrogenase [EC:1.1.-.-]	2.6625	0.2642	0.1619	0.4433	0.1240	M<N<MS
K09788: prpF; 2-methylaconitate isomerase [EC:5.3.3.-]	2.0562	0.3577	0.3939	0.2945	0.1916	N=M=MS
K11263: bccA, pccA; acetyl-CoA/propionyl-CoA carboxylase biotin carboxylase biotin carboxyl carrier protein [EC:6.4.1.2 6.4.1.3 6.3.4.14]	2.9798	0.2254	0.1625	0.1983	0.1270	MS<N<M
K11264: scpB, mmcD; methylmalonyl-CoA decarboxylase [EC:4.1.1.-]	0.9367	0.6260	0.4277	0.5495	0.4615	MS=M<N
K11381: bkdA; 2-oxoisovalerate dehydrogenase E1 component [EC:1.2.4.4]	3.7984	0.1497	0.2589	0.0881	0.3345	N=M=MS
K13788: pta; phosphate acetyltransferase [EC:2.3.1.8]	4.5538	0.1026	0.0570	0.1553	0.1668	N<MS<M
K13919: pduD; propanediol dehydratase medium subunit [EC:4.2.1.28]	2.2671	0.3219	0.2455	0.1967	0.2036	N=M=MS
K13920: pduE; propanediol dehydratase small subunit [EC:4.2.1.28]	0.6294	0.7300	0.4302	0.6418	0.5264	N=M=MS
K13921: pduQ; 1-propanol dehydrogenase	3.5443	0.1700	0.2145	0.1108	0.1264	N=M=MS
K13922: pduP; propionaldehyde dehydrogenase [EC:1.2.1.87]	0.2917	0.8643	0.4669	0.8868	0.5774	N=M=MS
K13923: pduL; phosphate propanoyltransferase [EC:2.3.1.222]	5.7657	0.0560	0.0209	0.3569	0.0355	N=M=MS
K15024: putative phosphotransacetylase [EC:2.3.1.8]	0.4303	0.8064	0.4331	0.7677	0.5758	N=M=MS
K18369: adh2; alcohol dehydrogenase [EC:1.1.1.-]	2.9123	0.2331	0.1748	0.2201	0.2333	N<MS<M
K18471: ydjG; methylglyoxal reductase [EC:1.1.1.-]	1.5466	0.4615	0.3185	0.2369	0.3279	M<N<MS

Gene family	MaAsLin2 analysis		
	Time point	*q* value	
		Non-AD vs. Mild	Non-AD vs. Moderate to Severe
K00005: gldA; glycerol dehydrogenase [EC:1.1.1.6]	N.S.*	N.S.	N.S.
K00016: ldh; L-lactate dehydrogenase [EC:1.1.1.27]	N.S.	N.S.	N.S.
K00048: fucO; lactaldehyde reductase [EC:1.1.1.77]	N.S.	N.S.	N.S.
K00086: dhaT; 1 3-propanediol dehydrogenase [EC:1.1.1.202]	N.S.	N.S.	N.S.
K00140: mmsA, iolA; malonate-semialdehyde dehydrogenase (acetylating) / methylmalonate-semialdehyde dehydrogenase [EC:1.2.1.18 1.2.1.27]	N.S.	N.S.	N.S.
K00166: bkdA1; 2-oxoisovalerate dehydrogenase E1 component alpha subunit [EC:1.2.4.4]	N.S.	N.S.	N.S.
K00167: bkdA2; 2-oxoisovalerate dehydrogenase E1 component beta subunit [EC:1.2.4.4]	N.S.	N.S.	N.S.
K00169: porA; pyruvate ferredoxin oxidoreductase alpha subunit [EC:1.2.7.1]	25-36 months	N.S.	0.1449
K00170: porB; pyruvate ferredoxin oxidoreductase beta subunit [EC:1.2.7.1]	25-36 months	0.1288	N.S.
K00171: porD; pyruvate ferredoxin oxidoreductase delta subunit [EC:1.2.7.1]	N.S.	N.S.	N.S.
K00172: porC porG; pyruvate ferredoxin oxidoreductase gamma subunit [EC:1.2.7.1]	N.S.	N.S.	N.S.
K00248: bcd; butyryl-CoA dehydrogenase [EC:1.3.8.1]	N.S.	N.S.	N.S.
K00382: lpd, pdhD; dihydrolipoamide dehydrogenase [EC:1.8.1.4]	N.S.	N.S.	N.S.
K00625: pta; phosphate acetyltransferase [EC:2.3.1.8]	N.S.	N.S.	N.S.
K00626: atoB; acetyl-CoA C-acetyltransferase [EC:2.3.1.9]	N.S.	N.S.	N.S.
K00656: pflD; formate C-acetyltransferase [EC:2.3.1.54]	N.S.	N.S.	N.S.
K00823: puuE; 4-aminobutyrate aminotransferase [EC:2.6.1.19]	N.S.	N.S.	N.S.
K00925: ackA; acetate kinase [EC:2.7.2.1]	N.S.	N.S.	N.S.
K00932: tdcD; propionate kinase [EC:2.7.2.15]	N.S.	N.S.	N.S.
K01026: pct; propionate CoA-transferase [EC:2.8.3.1]	N.S.	N.S.	N.S.
K01034: atoD; acetate CoA/acetoacetate CoA-transferase alpha subunit [EC:2.8.3.8 2.8.3.9]	N.S.	N.S.	N.S.
K01035: atoA; acetate CoA/acetoacetate CoA-transferase beta subunit [EC:2.8.3.8 2.8.3.9]	N.S.	N.S.	N.S.
K01659: prpC; 2-methylcitrate synthase [EC:2.3.3.5]	N.S.	N.S.	N.S.
K01682: acnB; aconitate hydratase 2 / 2-methylisocitrate dehydratase [EC:4.2.1.3 4.2.1.99]	N.S.	N.S.	N.S.
K01692: paaF, echA; enoyl-CoA hydratase [EC:4.2.1.17]	N.S.	N.S.	N.S.
K01699: pduC; propanediol dehydratase large subunit [EC:4.2.1.28]	N.S.	N.S.	N.S.
K01720: prpD; 2-methylcitrate dehydratase [EC:4.2.1.79]	N.S.	N.S.	N.S.
K01734: mgsA; methylglyoxal synthase [EC:4.2.3.3]	N.S.	N.S.	N.S.
K01782: fadJ; 3-hydroxyacyl-CoA dehydrogenase / enoyl-CoA hydratase / 3-hydroxybutyryl-CoA epimerase [EC:1.1.1.35 4.2.1.17 5.1.2.3]	N.S.	N.S.	N.S.
K01825: fadB; 3-hydroxyacyl-CoA dehydrogenase / enoyl-CoA hydratase / 3-hydroxybutyryl-CoA epimerase / enoyl-CoA isomerase [EC:1.1.1.35 4.2.1.17 5.1.2.3 5.3.3.8]	N.S.	N.S.	N.S.
K01847: MUT; methylmalonyl-CoA mutase [EC:5.4.99.2]	13-24 months	N.S.	0.0293
K01848: mcmA1; methylmalonyl-CoA mutase N-terminal domain [EC:5.4.99.2]	N.S.	N.S.	N.S.
K01849: mcmA2; methylmalonyl-CoA mutase C-terminal domain [EC:5.4.99.2]	N.S.	N.S.	N.S.
K01895: acs; acetyl-CoA synthetase [EC:6.2.1.1]	N.S.	N.S.	N.S.
K01902: sucD; succinyl-CoA synthetase alpha subunit [EC:6.2.1.5]	N.S.	N.S.	N.S.
K01903: sucC; succinyl-CoA synthetase beta subunit [EC:6.2.1.5]	N.S.	N.S.	N.S.
K01905: acdA; acetate---CoA ligase (ADP-forming) subunit alpha [EC:6.2.1.13]	N.S.	N.S.	N.S.
K01908: prpE; propionyl-CoA synthetase [EC:6.2.1.17]	N.S.	N.S.	N.S.
K01961: accC; acetyl-CoA carboxylase biotin carboxylase subunit [EC:6.4.1.2 6.3.4.14]	N.S.	N.S.	N.S.

K01962: accA; acetyl-CoA carboxylase carboxyl transferase subunit alpha [EC:6.4.1.2 2.1.3.15]	N.S.	N.S.	N.S.
K01963: accD; acetyl-CoA carboxylase carboxyl transferase subunit beta [EC:6.4.1.2 2.1.3.15]	N.S.	N.S.	N.S.
K01966: pccB; propionyl-CoA carboxylase beta chain [EC:6.4.1.3 2.1.3.15]	25-36 months	N.S.	0.1449
K02160: accB, bccP; acetyl-CoA carboxylase biotin carboxyl carrier protein	N.S.	N.S.	N.S.
K03417: prpB; methylisocitrate lyase [EC:4.1.3.30]	N.S.	N.S.	N.S.
K05606: epi; methylmalonyl-CoA/ethylmalonyl-CoA epimerase [EC:5.1.99.1]	N.S.	N.S.	N.S.
K07250: gabT; 4-aminobutyrate aminotransferase / (S)-3-amino-2-methylpropionate transaminase / 5-aminovalerate transaminase [EC:2.6.1.19 2.6.1.22 2.6.1.48]	N.S.	N.S.	N.S.
K08325: yqhD; NADP-dependent alcohol dehydrogenase [EC:1.1.-.-]	N.S.	N.S.	N.S.
K09788: prpF; 2-methylaconitate isomerase [EC:5.3.3.-]	N.S.	N.S.	N.S.
K11263: bccA, pccA; acetyl-CoA/propionyl-CoA carboxylase biotin carboxylase biotin carboxyl carrier protein [EC:6.4.1.2 6.4.1.3 6.3.4.14]	N.S.	N.S.	N.S.
K11264: scpB, mmcD; methylmalonyl-CoA decarboxylase [EC:4.1.1.-]	N.S.	N.S.	N.S.
K11381: bkdA; 2-oxoisovalerate dehydrogenase E1 component [EC:1.2.4.4]	N.S.	N.S.	N.S.
K13788: pta; phosphate acetyltransferase [EC:2.3.1.8]	N.S.	N.S.	N.S.
K13919: pduD; propanediol dehydratase medium subunit [EC:4.2.1.28]	N.S.	N.S.	N.S.
K13920: pduE; propanediol dehydratase small subunit [EC:4.2.1.28]	N.S.	N.S.	N.S.
K13921: pduQ; 1-propanol dehydrogenase	N.S.	N.S.	N.S.
K13922: pduP; propionaldehyde dehydrogenase [EC:1.2.1.87]	N.S.	N.S.	N.S.
K13923: pduL; phosphate propanoyltransferase [EC:2.3.1.222]	N.S.	N.S.	N.S.
K15024: putative phosphotransacetylase [EC:2.3.1.8]	N.S.	N.S.	N.S.
K18369: adh2; alcohol dehydrogenase [EC:1.1.1.-]	N.S.	N.S.	N.S.
K18471: ydjG; methylglyoxal reductase [EC:1.1.1.-]	N.S.	N.S.	N.S.

*N.S., Not significant

Table S11. Comparison of the shifted KEGG Orthology (KO) according to age among phenotypic groups. Significantly changed categories according to age were selected using MaAsLin2 (q < 0.05) and the random forest model (the lowest cross-validation error). KO features of the gut microbiome at 6 months were used as a reference for MaAsLin2 analysis. Kruskal–Wallis and Dunn's post-hoc tests were performed to calculate the significant differences between groups. *P*-value was adjusted using the Benjamini–Hochberg method. N: Non-AD, M: Mild AD, MS: Moderate to Severe AD.

KEGG Orthology	MaAsLin2 analysis					
	Time point	Coefficient	N	N not 0	*P* value	*q* value
ko00140: Steroid hormone biosynthesis	25-36 months	0.00148	112	72	2.93E-14	5.70E-12
ko00998: Biosynthesis of various secondary metabolites - part 2	25-36 months	0.00282	112	107	4.43E-14	7.47E-12
ko00625: Chloroalkane and chloroalkene degradation	25-36 months	-0.00141	112	112	1.93E-14	4.07E-12
ko04919: Thyroid hormone signaling pathway	25-36 months	0.00237	112	112	2.78E-20	7.03E-17
ko99975: Protein processing	25-36 months	-0.00097	112	112	6.88E-20	7.90E-17
ko03013: RNA transport	25-36 months	0.00141	112	112	1.07E-14	2.45E-12
ko00121: Secondary bile acid biosynthesis	25-36 months	0.00151	112	112	6.32E-15	1.60E-12
ko04152: AMPK signaling pathway	25-36 months	0.00210	112	112	1.20E-19	7.90E-17
ko00525: Acarbose and validamycin biosynthesis	25-36 months	0.00154	112	112	3.16E-17	1.33E-14
ko00430: Taurine and hypotaurine metabolism	25-36 months	0.00043	112	112	1.71E-09	7.00E-08
ko00523: Polyketide sugar unit biosynthesis	25-36 months	0.00190	112	112	1.25E-19	7.90E-17
ko99993: Cell motility	25-36 months	-0.00251	112	100	7.43E-17	2.68E-14
ko01051: Biosynthesis of ansamycins	25-36 months	0.00215	112	112	5.04E-19	2.55E-16
ko99999: Others - unclassified metabolism	25-36 months	-0.00133	112	112	1.54E-11	1.25E-09
ko04011: MAPK signaling pathway - yeast	25-36 months	0.00074	112	104	1.13E-04	1.44E-03
ko99973: Transcription	25-36 months	-0.00190	112	112	2.37E-13	2.60E-11
ko00460: Cyanoamino acid metabolism	25-36 months	0.00068	112	112	4.15E-10	2.10E-08
ko00053: Ascorbate and aldarate metabolism	25-36 months	-0.00097	112	112	1.46E-09	6.59E-08
ko00120: Primary bile acid biosynthesis	25-36 months	0.00177	112	111	3.93E-12	3.82E-10

KEGG Orthology	6 months						7-12 months					
	Kruskal-Wallis test		Adjusted *P* value (Dunn's Post-hoc test)			Enrichment	Kruskal-Wallis test		Adjusted *P* value (Dunn's Post-hoc test)			Enrichment
	R-squared	*P* value	Non-AD vs. Mild	Non-AD vs. Moderate to Severe	Mild vs. Moderate to Severe		R-squared	*P* value	Non-AD vs. Mild	Non-AD vs. Moderate to Severe	Mild vs. Moderate to Severe	
ko00140: Steroid hormone biosynthesis	2.9024	0.2343	0.1598	0.2112	0.1486	N=M=MS	3.2847	0.1935	0.4479	0.1411	0.1416	N=M<MS
ko00998: Biosynthesis of various secondary metabolites - part 2	8.0073	0.0182	0.0928	0.0285	0.0072	MS<N<M	0.0919	0.9551	0.4635	0.6740	1.0000	N<M<MS
ko00625: Chloroalkane and chloroalkene degradation	1.5600	0.4584	0.2627	0.2877	0.3446	M<MS<N	1.5450	0.4619	0.2857	0.3664	0.2884	MS<M<N
ko04919: Thyroid hormone signaling pathway	5.3969	0.0673	0.4655	0.0396	0.0337	MS<N<M	0.4767	0.7879	0.7585	0.3566	0.5251	M<MS<N
ko99975: Protein processing	3.0914	0.2132	0.2794	0.1100	0.1301	M<N<MS	0.7792	0.6773	0.3288	0.5870	0.4384	MS<M<N
ko03013: RNA transport	2.7911	0.2477	0.1864	0.1922	0.1519	MS<N<M	1.7835	0.4099	0.2485	0.4606	0.3586	M<N<MS
ko00121: Secondary bile acid biosynthesis	2.3812	0.3040	0.2420	0.1869	0.1889	N<MS<M	1.2178	0.5440	0.2354	0.4422	0.4570	M<MS<N
ko04152: AMPK signaling pathway	5.1534	0.0760	0.3828	0.0539	0.0296	MS<N<M	1.1360	0.5667	0.5144	0.3550	0.3344	M<MS<N
ko00525: Acarbose and validamycin biosynthesis	6.0703	0.0581	0.4891	0.0264	0.0273	MS<M<N	0.8742	0.6459	0.3787	0.5252	0.3410	MS<M<N
ko00430: Taurine and hypotaurine metabolism	3.3652	0.1859	0.3624	0.1008	0.1377	MS<M<N	2.0675	0.3557	0.3885	0.1845	0.3187	MS<N<M
ko00523: Polyketide sugar unit biosynthesis	7.3509	0.0253	0.2883	0.0102	0.0394	M<MS<N	1.1405	0.5654	0.2697	0.4456	0.3946	MS<M<N

ko99993: Cell motility	0.9650	0.6172	0.4414	0.5518	0.2767	MS<N<M	5.3575	0.0686	0.4554	0.0754	0.0465	M<N<MS
ko01051: Biosynthesis of ansamycins	8.7967	0.0123	0.1987	0.0086	0.0065	N<MS<M	0.4682	0.7913	0.4890	0.4944	0.7667	MS<M<N
ko99999: Others - unclassified metabolism	2.4368	0.2957	0.2053	0.2136	0.1778	M<MS<N	0.7922	0.6730	0.7016	0.4251	0.3543	MS<N<M
ko04011: MAPK signaling pathway - yeast	1.6230	0.4442	0.2430	0.4521	0.4401	M<MS<N	7.5256	0.0232	0.0672	0.0102	0.0662	N<M<MS
ko99973: Transcription	0.6270	0.7309	0.3836	0.3950	0.6544	M<MS<N	0.0698	0.9657	0.4410	1.0000	0.6546	N<M<MS
ko00460: Cyanoamino acid metabolism	3.6285	0.1630	0.1535	0.1477	0.0902	N<MS<M	0.3307	0.8476	0.8532	0.5345	0.3996	M<MS<N
ko00053: Ascorbate and aldarate metabolism	0.2675	0.8748	0.3849	0.5524	0.9078	M<N<MS	2.2380	0.3266	0.2438	0.2394	0.2251	MS<M<N
ko00120: Primary bile acid biosynthesis	2.9744	0.2260	0.1937	0.1671	0.1273	N<MS<M	0.8687	0.6477	0.3477	0.5850	0.3735	M<MS<N

KEGG Orthology	13-24 months months						25-36 months months					
	Kruskal-Wallis test		Adjusted *P* value (Dunn's Post-hoc test)			Enrichment	Kruskal-Wallis test		Adjusted *P* value (Dunn's Post-hoc test)			Enrichment
	R-squared	*P* value	Non-AD vs. Mild	Non-AD vs. Moderate to Severe	Mild vs. Moderate to Severe		R-squared	*P* value	Non-AD vs. Mild	Non-AD vs. Moderate to Severe	Mild vs. Moderate to Severe	
ko00140: Steroid hormone biosynthesis	3.8452	0.1462	0.0570	0.0849	0.4722	MS<M<N	9.7321	0.0077	0.0040	0.0571	0.0681	M<MS<N
ko00998: Biosynthesis of various secondary metabolites - part 2	2.7517	0.2526	0.1534	0.2051	0.1826	M<MS<N	9.3562	0.0093	0.0509	0.0283	0.0049	M<N<MS
ko00625: Chloroalkane and chloroalkene degradation	6.4119	0.0405	0.0196	0.0191	0.4066	N<M<MS	8.4919	0.0143	0.0101	0.1955	0.0061	MS<N<M
ko04919: Thyroid hormone signaling pathway	0.1207	0.9414	1.0000	0.6205	0.4200	MS<M<N	0.9381	0.6256	0.3028	0.3822	0.5283	M<N<MS
ko99975: Protein processing	2.4445	0.2946	0.4663	0.2225	0.2414	N<M<MS	4.5853	0.1010	0.0488	0.3318	0.0703	MS<N<M
ko03013: RNA transport	2.5966	0.2730	0.1827	0.1776	0.2300	M<MS<N	2.9191	0.2323	0.0949	0.3646	0.1485	M<N<MS
ko00121: Secondary bile acid biosynthesis	0.2862	0.8667	0.4017	0.9355	0.5519	M<N<MS	1.2768	0.5281	0.4305	0.3925	0.2931	MS<N<M
ko04152: AMPK signaling pathway	0.1051	0.9488	1.0000	0.4400	0.6102	MS<M<N	1.0741	0.5845	0.2934	0.3567	0.4814	M<N<MS
ko00525: Acarbose and validamycin biosynthesis	0.2042	0.9029	0.5697	0.4880	1.0000	M<N<MS	0.0892	0.9564	0.6357	1.0000	0.4967	M<N<MS
ko00430: Taurine and hypotaurine metabolism	0.0447	0.9779	0.6582	1.0000	0.4711	MS<N<M	0.4891	0.7831	0.7452	0.3690	0.5181	M<MS<N
ko00523: Polyketide sugar unit biosynthesis	1.2147	0.5448	0.4330	0.4361	0.4113	M<N<MS	0.2324	0.8903	0.4854	0.9735	0.5466	MS<N<M
ko99993: Cell motility	3.3607	0.1863	0.1318	0.1810	0.1487	N<MS<M	0.9228	0.6304	0.4386	0.5710	0.3420	MS<N<M
ko01051: Biosynthesis of ansamycins	0.1762	0.9156	1.0000	0.5410	0.4534	MS<M=N	1.0455	0.5929	0.3234	0.3329	0.5133	M<N<MS
ko99999: Others - unclassified metabolism	2.4532	0.2933	0.1929	0.1162	0.4017	N<M<MS	0.1666	0.9201	0.4099	0.5910	1.0000	N<M<MS
ko04011: MAPK signaling pathway - yeast	2.2049	0.3321	0.2827	0.2264	0.2267	M<MS<N	14.0595	0.0009	0.0956	0.0003	0.1307	MS<M<N
ko99973: Transcription	8.1031	0.0174	0.0062	0.0096	0.4777	N<M<MS	2.3553	0.3080	0.1570	0.2979	0.1910	MS<N<M
ko00460: Cyanoamino acid metabolism	0.7000	0.7047	0.4169	0.7535	0.3809	N<M<MS	0.5144	0.7732	0.5763	0.7187	0.4150	MS<N<M
ko00053: Ascorbate and aldarate metabolism	3.9628	0.1379	0.0896	0.0698	0.2988	N<M<MS	2.2211	0.3294	0.3072	0.1651	0.2861	MS<N<M
ko00120: Primary bile acid biosynthesis	0.2261	0.8931	0.4940	0.9741	0.4953	M<MS<N	2.5176	0.2840	0.1689	0.3446	0.1752	N<MS<M

Table S12. Primer list for gene expression of mice colon.

Target gene	Primers	Sequence (5′-3′)
β-actin	β-actin_forward	TTCGTTGCCGGTCCACA
	β-actin_reverse	ACCAGCGCAGCGATATCG
Pparg	Pparg_forward	CGGTTTCAGAAGTGCCTTG
	Pparg_reverse	GGTTCAGCTGGTCGATATCAC
Gpr109a	Gpr109a_forward	ATGGCGAGGCATATCTGTGTAGCA
	Gpr109a_reverse	TCCTGCCTGAGCAGAACAAGATGA
Gpr41	Gpr41_forward	GGGGTCGATACAAGAGT
	Gpr41_reverse	CTGGCGGAGCTACGTGCT
Gpr43	Gpr43_forward	CCACTGTATGGAGTGATCGCTG
	Gpr43_reverse	GGGTGAAGTTCTAGCAGGT
Foxp3	Foxp3_forward	TGCGACCCCCTTTCACCT
	Foxp3_reverse	AGTGTCCTCTGCCTCTCC
Il10	Il10_forward	CCTCAGGATGCGGCTGAG
	Il10_reverse	GCTCCACTGCCTTGCTCTTATT
Tnfa	Tnfa_forward	ACGGCATGGATCTCAAAGAC
	Tnfa_reverse	GTGGGTGAGGAGCACGTAGT
Il1b	Il1b_forward	GCAACTGTTCCTGAACTCAAC
	Il1b_reverse	ATCTTTTGGGGTCCGTCAACT
Il6	Il6_forward	TAGTCCTTCCTACCCCAATTTCC
	Il6_reverse	TTGGTCCTTAGCCACTCCTTC
